# The loss and recovery of vertebrate vision examined in microplates

**DOI:** 10.1371/journal.pone.0183414

**Published:** 2017-08-17

**Authors:** Robert J. Thorn, Danielle E. Clift, Oladele Ojo, Ruth M. Colwill, Robbert Creton

**Affiliations:** 1 Department of Molecular Biology, Cell Biology and Biochemistry, Brown University, Providence, Rhode Island, United States of America; 2 Department of Cognitive, Linguistic and Psychological Sciences, Brown University, Providence, Rhode Island, United States of America; National Institutes of Health, UNITED STATES

## Abstract

Regenerative medicine offers potentially ground-breaking treatments of blindness and low vision. However, as new methodologies are developed, a critical question will need to be addressed: how do we monitor *in vivo* for functional success? In the present study, we developed novel behavioral assays to examine vision in a vertebrate model system. In the assays, zebrafish larvae are imaged in multiwell or multilane plates while various red, green, blue, yellow or cyan objects are presented to the larvae on a computer screen. The assays were used to examine a loss of vision at 4 or 5 days post-fertilization and a gradual recovery of vision in subsequent days. The developed assays are the first to measure the loss and recovery of vertebrate vision in microplates and provide an efficient platform to evaluate novel treatments of visual impairment.

## Introduction

Visual impairment has been estimated to affect 285 million people worldwide; 246 million people have low vision and 39 million people are blind [1]. While visual impairment is generally irreversible, it may be possible to treat blindness and low vision using novel methodologies in regenerative medicine. Phase I and Phase II clinical trials are in progress using stem cell-based therapies to treat retinal disease [2]. In addition, comparative studies in vertebrate model systems have provided a better understanding of the signaling pathways that regulate regenerative neurogenesis and these signaling pathways may be used to stimulate endogenous regeneration of the visual system [3,4]. However, as novel methodologies are developed, a critical question will need to be addressed: how do we monitor *in vivo* for functional success?

The analysis of visually-guided behaviors in zebrafish larvae provides an effective approach to examine visual function. The development, anatomy and physiology of the visual system is highly conserved in vertebrate species and zebrafish larvae have a cone-dominated retina for full-color vision [5,6]. Hundreds of embryos can be collected from the bottom of a tank on a daily basis. The larvae hatch from their chorion around 3 days post-fertilization (dpf) and have a functional visual system at 5 dpf [6]. By analyzing visually-guided behaviors in zebrafish larvae, it is possible to detect functional defects, even when the visual system appears normal by morphological criteria. For example, specific visual defects have been identified by measuring the optokinetic response (OKR) and optomotor response (OMR). In the OKR assay, zebrafish larvae are immobilized inside a cylindrical drum with rotating black and white stripes. The eyes of the larvae follow the stripes, a response that gradually develops between 3–4 dpf and can be reliably measured at 4–5 dpf [7,8]. OKR assays have been used in zebrafish mutagenesis screens to identify a broad range of genes important for vision [7,9,10,11]. In OMR assays, zebrafish larvae are placed in elongated swimming tracks and are then presented with moving black and white or colored stripes [11,12,13]. The larvae swim in the same direction as the moving stripes, a response that can be reliably measured at 6–7 dpf [11,13]. Similar to OKR assays, OMR assays have been used in mutagenesis screens to identify a wide variety of genes important for vision [10,11]. The behavior of zebrafish larvae can be examined in microplates with commercially available imaging systems, providing unique opportunities for high-throughput applications. Imaging systems such as the ZebraBox from ViewPoint and DanioVision from Noldus are equipped with infrared lights for imaging zebrafish larvae and white lights for studying light-dependent locomotor responses [14,15,16,17]. However, these microplate imaging systems are not equipped for a display of more complex visual stimuli.

In the current study, we present several novel assays for measuring visually-guided behaviors in microplates. In the assays, zebrafish larvae are imaged in multiwell or multilane plates while moving objects are presented to the larvae on a computer screen. The developed assays were used for measuring a loss and recovery of visual function in zebrafish larvae. The developed assays are the first to measure the loss and recovery of vertebrate vision in microplates and provide an efficient platform to evaluate novel treatments of visual impairment.

## Materials and methods

### Ethics statement

Zebrafish larvae were anesthetized with Tricaine (also known as MS-222) during the UV exposure at 5 days post-fertilization. The use of zebrafish in our studies is in compliance with federal (PHS, USDA) and international (AAALAC) guidelines and has been approved by Brown University's Institutional Animal Care and Use Committee (IACUC).

### Zebrafish

Adult wild type zebrafish (*Danio rerio*) were originally obtained from Carolina Biological and have been maintained at Brown University as a genetically diverse outbred strain. Zebrafish spawn in the morning when kept on a 14 hr light, 10 hr dark cycle in a mixed male and female population. A few tanks with adult fish will produce hundreds of embryos on a daily basis. Zebrafish embryos from 0–3 days post-fertilization (dpf) and zebrafish larvae from 3–7 dpf were grown at 28.5°C on a 12 hour light / 12 hour dark cycle in egg water, containing 60 mg/l sea salt (Instant Ocean) and 0.25 mg/l methylene blue in deionized water. The embryos and larvae were grown in 2L culture trays and were assigned randomly to different experimental groups prior to experimental manipulation or imaging. The sex of embryos and larvae cannot be determined at these early stages, since zebrafish use elusive polygenic factors for sex determination and both males and females have juvenile ovaries between 2.5 and 4 weeks of development [18]. Zebrafish larvae were imaged at 4–7 dpf when the larvae use nutrients that are available in their yolk sac and display a range of locomotor behaviors. The larvae are approximately 4 mm long during this period. The larvae are euthanized using an overdose of tricaine (0.04% w/v, pH7). After 20 minutes, the euthanized larvae are transferred to a container with bleach (sodium hypochlorite 6.15%, diluted 1:5 in egg water).

### UV illumination

Visual defects were induced by UV illumination using a protocol adapted from Meyers et al. 2012 [19]. In short, 5 dpf zebrafish larvae were anesthetized with 0.012% tricaine (also known as MS-222) pH7 and transferred to a GeneTools light box containing a cooled LED array, which emits 900 mW of 365 nm light. After 5 min UV illumination, larvae were washed in egg water and were allowed to recover from the anesthesia for 2 hours prior to the analysis of visually-guided behaviors. As controls, larvae were left untreated or were exposed to 0.012% tricaine, without UV-illumination, washed in egg water and were again allowed to recover from the anesthesia for 2 hours prior to the analysis of visually-guided behaviors. The protocol for UV illumination was modified for the rotating-cross assays. We increased the period of UV illumination to 5½ minutes to further reduce visual responses at 5 dpf. In addition, we reduced the tricaine concentration to 0.00025%, since preliminary experiments revealed effects of higher tricaine concentrations in the behavioral assays.

### Morpholino injections

To specifically suppress photoreceptor function, Pde6c signaling was knocked down by morpholino injection. A *pde6c* morpholino designed by GeneTools (GCTATCCTTGTCTGCCATGTTTGAA) targets the translational start site of both *pde6c* transcripts in zebrafish (ENSDART0000016224.7 and ENSDART00000169073.1). The GeneTools standard control morpholino (CCTCTTACCTCAGTTACAATTTATA) was injected as a negative control. The morpholinos were dissolved in ultrapure water at a 1mM stock concentration and stored in the dark at room temperature. Prior to injection, the morpholinos were diluted into an injection solution containing 0.6 mM morpholino and 0.5 mM fluorescein dextran (MW 10,000) in ultrapure water. Zebrafish embryos were injected at the 1 to 4 cell stage with 1–2 nl of the control or pde6c morpholino. After injection, embryos were raised in a 50 ml Petri dish at 28.5°C. All embryos were screened at 1 dpf for fluorescein dextran fluorescence using a NightSea fluorescence system attached to a dissection scope.

### Western blots

Embryos were deyolked in batches as outlined in Link et al. [20]. Briefly, 15–20 larvae were placed in a 1.5ml Eppendorf tube. Egg water was removed and 1 ml deyolk buffer (55 mM NaCl, 1.8 mM KCl, 1.25 mM NaHCO_3_) was added. Larvae were pipetted through a p200 pipette tip to disrupt the yolk, and then agitated at 1100 RPM for 5 minutes. They were then centrifuged at 300g for 30 seconds. The larvae were then washed twice by removing the deyolk buffer, adding 1mL wash buffer (110 mM NaCl, 3.5 mM KCl, 2.7 mM CaCl2, 10 mM Tris-Cl pH8.5), agitating at 1100 RPM for for 2 minutes, and centrifuging at 300g for 1 minute. After washes, all liquid was removed, and 4 ul 1x SDS buffer (5% 2-Mercapto Ethanol, 2% SDS, 5% glycerol, 0.05 mM Tris pH 6.8, 0.017% Bromophenol Blue in water) per larva was added. The samples were homogenized and 6 larval equivalents of protein isolate was assayed. The blots were labeled with a rabbit-anti-pde6c polyclonal antibody (Abcam ab198744) and a mouse-anti-alpha tubulin monoclonal antibody (Sigma T6199). A HRP-conjugated goat-anti-rabbit polyclonal antibody (Abcam ab6721) and a HRP-conjugated goat-anti-mouse polyclonal antibody (Abcam ab97265) were used as secondary antibodies. The HRP label was imaged using a SuperSignal (TM) West Pico PLUS Chemiluminsecent Substrate (Thermo Scientific 34579) and an Azure c600 imaging system (Azure Biosystems).

### The zebrafish imaging system

Visually-guided behaviors were recorded using a zebrafish imaging system described previously [21,22], with minor modifications. The imaging system is housed in a 180 × 40 × 40 cm cabinet. The top shelf of the cabinet holds an 18 megapixel Canon EOS Rebel T6 digital camera with an EF-S 55–250 mm f/4.0–5.6 IS zoom lens. The camera is connected to a continuous power supply (Canon ACK-E10 AC Adapter) and is controlled by a laptop computer using Canon's Remote Capture software, which is included with the camera. The software is set to interval mode to acquire high-resolution images every 6 sec. The bottom shelf of the cabinet holds a second laptop (Acer Aspire 5517) with a 15.6 inch LCD screen, which is used to provide visual stimuli to the larvae. The 15.6 inch LCD screen has a 1366 × 768 pixel resolution and a brightness of 220 cd/m^2^. To avoid moiré patterns in the images, a plastic diffuser (Pendaflex 52345) is placed on the LCD screen. The plates with the larvae are placed on top of the diffuser approximately 10 minutes prior to imaging and the LCD screen warms the plates to 26°C (4°C above ambient). The zebrafish imaging system can be duplicated on a limited budget and can image 4 microplates simultaneously, while retaining sufficient resolution to identify the location and orientation of zebrafish larvae. Brown University currently has 8 zebrafish imaging systems set up in two laboratories, with a combined capacity of imaging 32 microplates simultaneously. The scalable capacity of the system makes it possible to examine subtle visual defects in more detail or initiate medium- to high-throughput applications. The imaging system is unique in the display of complex visual stimuli in microplates.

### Assays for visually-guided behaviors in 5-lane plates

The 5-lane plates were created as described previously [22,23]. Briefly, a one-well plate (ThermoFisher Scientific, Cat. No. 267060) is filled with 50 ml liquid agarose (0.8% agarose in deionized water at 70–80°C). A custom-designed 5-lane mold is placed on top of the agarose, which gels as it cools down to room temperature. After removing the mold, the plate has 5 lanes that are each 70 mm long × 18 mm wide with 60° sloping edges to reach a 66 mm × 14 mm bottom at a 3.5 mm depth. The optics of the plate is optimal when each lane is filled precisely to the rim with egg water. Zebrafish larvae are transferred to the plates 20 min prior to imaging. In the imaging system, visual stimuli are shown to the larvae as PowerPoint presentations. Our previous studies have shown that 5–7 dpf larvae avoid a red bar that moves up and down in the upper half of a 5-lane plate [22,23]. The current study introduces the following new assays for 5-lane plates. 1) The bar-dots assay: in this assay larvae are imaged for 15 min on a white background, 15 min with a moving red bar in the upper half of a lane, and 15 min with 0.5 mm red dots moving up in a lane (S1 File). 2) The two-bar assay: in this assay larvae are imaged for 15 min on a white background, 15 min with a moving red bar in the upper half of a lane, and 15 min with a moving red bar in the lower half of a lane (S2 File). 3) The 4x repeated two-bar assay: in this assay larvae are imaged for 20 min on a white background, 10 min with a moving red bar in the upper half of a lane, and 10 min with a moving red bar in the lower half of a lane. The two-bar sequence is shown to the larvae four times in a row (S3 File). 4) The bar-dots RGBY assay: in this assay larvae are imaged for 10 min on a white background, 10 min with a moving bar in the upper half of a lane, and 10 min with 1 mm dots moving up in a lane. The blank-bar-dots sequence is shown to the larvae in red, green, blue and yellow (S4 File).

### Assays for visually-guided behaviors in 6-well plates

The 6-well plates were created as described previously [21]. In summary, the wells of a 6-well plate are filled with 5 ml liquid agarose (0.8% agarose in deionized water at 70–80°C). After the agarose solidifies, a plastic vial is used to stamp a 27 mm diameter × 5 mm deep hole in the agarose. The optics of the plate is optimal when each well is filled with egg water precisely to the rim of the agarose. Zebrafish larvae are transferred to the wells 20 min prior to imaging. The current study introduces the following new assays for 6-well plates. 1) Rotating spectral cross assay: larvae are imaged for 20 min on a light background, 10 min with a cross that rotates in a clockwise direction, and 10 min with a cross that rotates in a counter-clockwise direction. The pair of rotating crosses are shown to the larvae in red, green, blue, yellow, and cyan and rotate at 90°/5 sec which corresponds to 3 rounds per minute. The background is set to a light gray for optimal color separation during the image analysis (S5 File). 2) The 4x repeated red cross assay: larvae are imaged for 20 min on a light gray background, 10 min with a red cross that rotates in a clockwise direction, and 10 min with a red cross that rotates in a counter-clockwise direction. The two-cross sequence is shown to the larvae four times in a row and crosses rotate at 3 rounds per minute (S6 File).

### Image analysis

Acquired images were analyzed in ImageJ using a custom-developed macro. This macro (version 26bc) can analyze four microplates, with multiple treatment groups and changing visual stimuli over time. The software asks the user to enter information about the wells and the periods with different visual stimuli. It opens the first image, splits the color channels, and selects a channel in which the visual stimuli and background have similar intensities. It then subtracts the background, applies an auto-threshold for individual wells, carries out a particle analysis on individual wells, and logs various parameters of the larvae in a ‘Results’ file. This process is automatically repeated for all subsequent images in a series. The Results file is then sorted based on well number and imported into a MS Excel template. This template compares the larval centroids with the center of the lane to determine if a larva is located ‘up’ or ‘down’ in the lane. The template also compares the larval centroids with the larval center of the ‘bounding box’ to determine the larval orientation in specific quadrants of the well. For example, a larva is considered to have a clockwise orientation if it faces up (±45°) or right (±45°) in the top-left quarter of a well. The ImageJ macro (S7 File) and MS Excel template (S8 File) are available in the supplementary information and future updates will be posted on Brown University’s zebrafish website: https://www.brown.edu/research/projects/zebrafish/ The automated image analysis contributes to an unbiased approach in studying visually-guided behavior. For example, effects of observer bias and observer fatigue can be avoided.

### Statistical analysis

The obtained results were averaged in MS Excel. To assure that all data points are independent, even in wells with multiple larvae, we analyzed the data on a per well basis (n = number of wells). Within each well, we consider all ‘larval measurements’ equally. For example, a well with 5 larvae imaged for 10 minutes at 10 frames per minute, will provide 500 larval measurements, which are averaged as a whole. The averaging over a 10 minute period reduces variability between wells and makes it possible to obtain reliable data with a relatively low number of larvae. However, it is possible to analyze the data at shorter intervals, which could provide additional information on changes within the 10 minute period. The percentage of larval measurements in the upper half of the lane (% up) and the percentage of larval measurements with a clockwise orientation (% cw) have a normal distribution. Differences in % up and % cw were tested for significance using a two-tailed t-test with unequal variance. The calculated p-values were adjusted with a Bonferroni correction for multiple comparisons. Differences in behavior were considered significant when p<0.01. When differences in behavior were significant at a 95% confidence limit (p<0.05), the results were described in the text, but not indicated with an asterisk in the graphs. The conservative Bonferroni correction and stringent p-value help to avoid type I errors (false positives), which is important in assay development. For repeated measures over time, we used a one-way repeated measures analysis of variance (ANOVA) with stimulus acting as the independent variable and %up or %cw as dependent variables, with a Greenhouse-Geisser correction when sphericity was violated. All ANOVAs were carried out using IBM SPSS Statistics 24.

### Code availability

The ImageJ macro and MS Excel template for automated analyses of behavior are included in the supplementary information.

### Data availability

The original imaging data is available upon request. The PowerPoint files with visual stimuli are included in the supplementary information.

## Results

### Automated analysis of behavior

**Fig 1:**
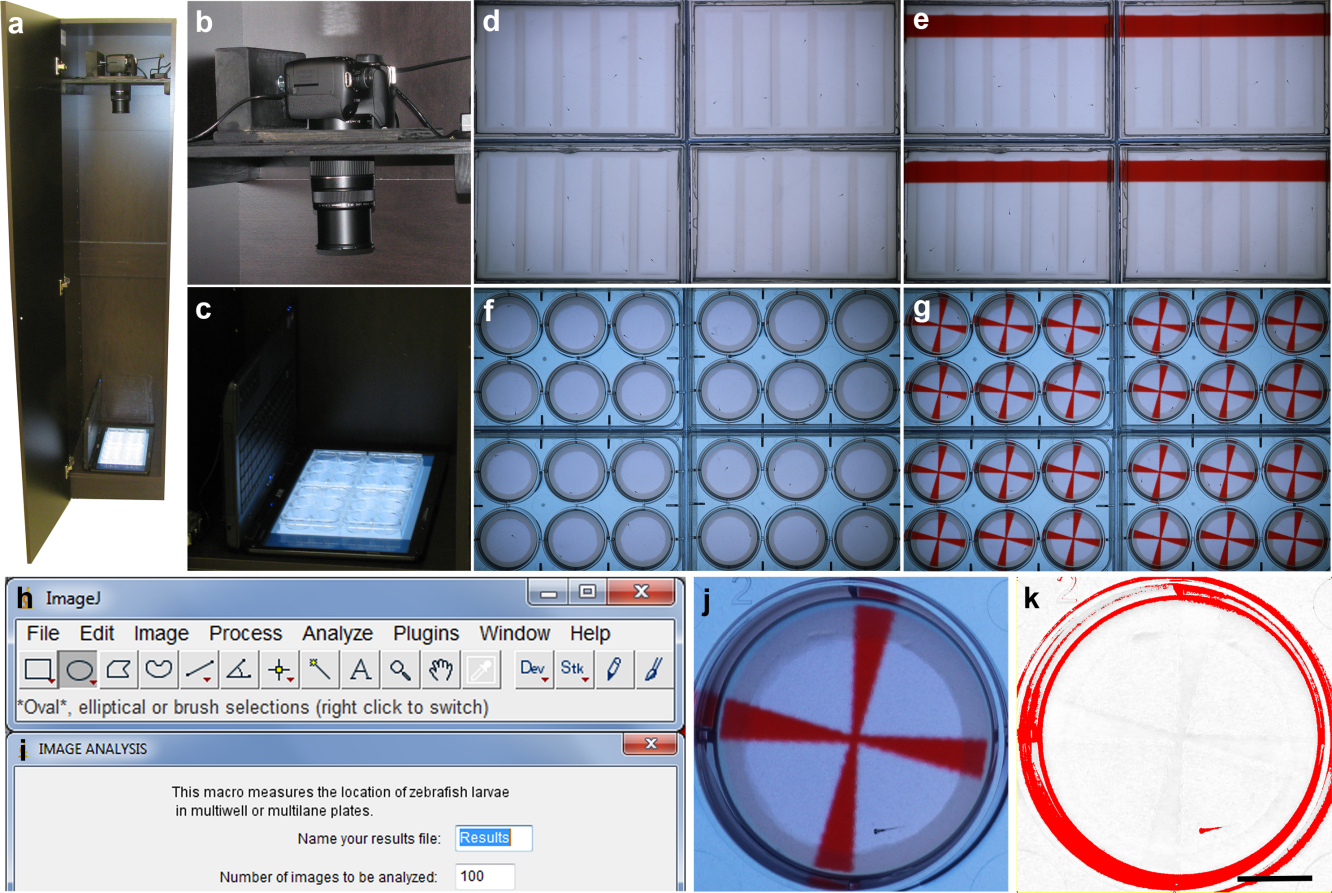
Imaging system for automated analyses of behavior. a) Imaging cabinet. b) Canon EOS Rebel T6 camera for acquisition of 18 megapixel color images. c) Four microplates with zebrafish larvae on the screen of a laptop. Visual stimuli are shown to the larvae using PowerPoint presentations. d) Four 5-lane plates without visual stimuli. e) Four 5-lane plates with a moving red bar. f) Four 6-well plates without visual stimuli. g) Four 6-well plates with a rotating red cross. h) The acquired images are analyzed in ImageJ. i) An ImageJ macro was developed for the automated analysis of large imaging files. The macro opens the first image, splits the color channels, selects a channel in which the visual stimuli and background have similar intensities, subtracts the background, applies a threshold, carries out a particle analysis, logs the measured coordinates, and automatically repeats this process for subsequent images in the series. j) Well with an agarose ring, a red cross and a 5 day-old zebrafish larva. k) The same well in the red channel after background subtraction and a threshold for dark objects. The acquired images showing four plates have sufficient resolution for measuring the location and the orientation of individual larvae. Scale bar = 1 cm.

Visually-guided behaviors were measured using a custom-built imaging system for automated analyses of behavior [21,22]. This imaging system is easy to use, can be built on a limited budget and makes use of open-source software for image analysis (Fig 1). Images are acquired using an 18 megapixel Canon camera and visual stimuli are shown to zebrafish larvae as PowerPoint presentations on a computer screen. In the current study, we developed a new ImageJ macro and MS Excel template for automated analyses of microplates with changing visual stimuli over time. The macro includes dialog boxes and algorithms for the identification of experimental groups and periods with different visual stimuli. The imaging system and software make it possible to measure the location and orientation of larvae in a set of four microplates, while moving objects with different colors are presented to the larvae.

### Assays for visually-guided behaviors in 5-lane plates

**Fig 2:**
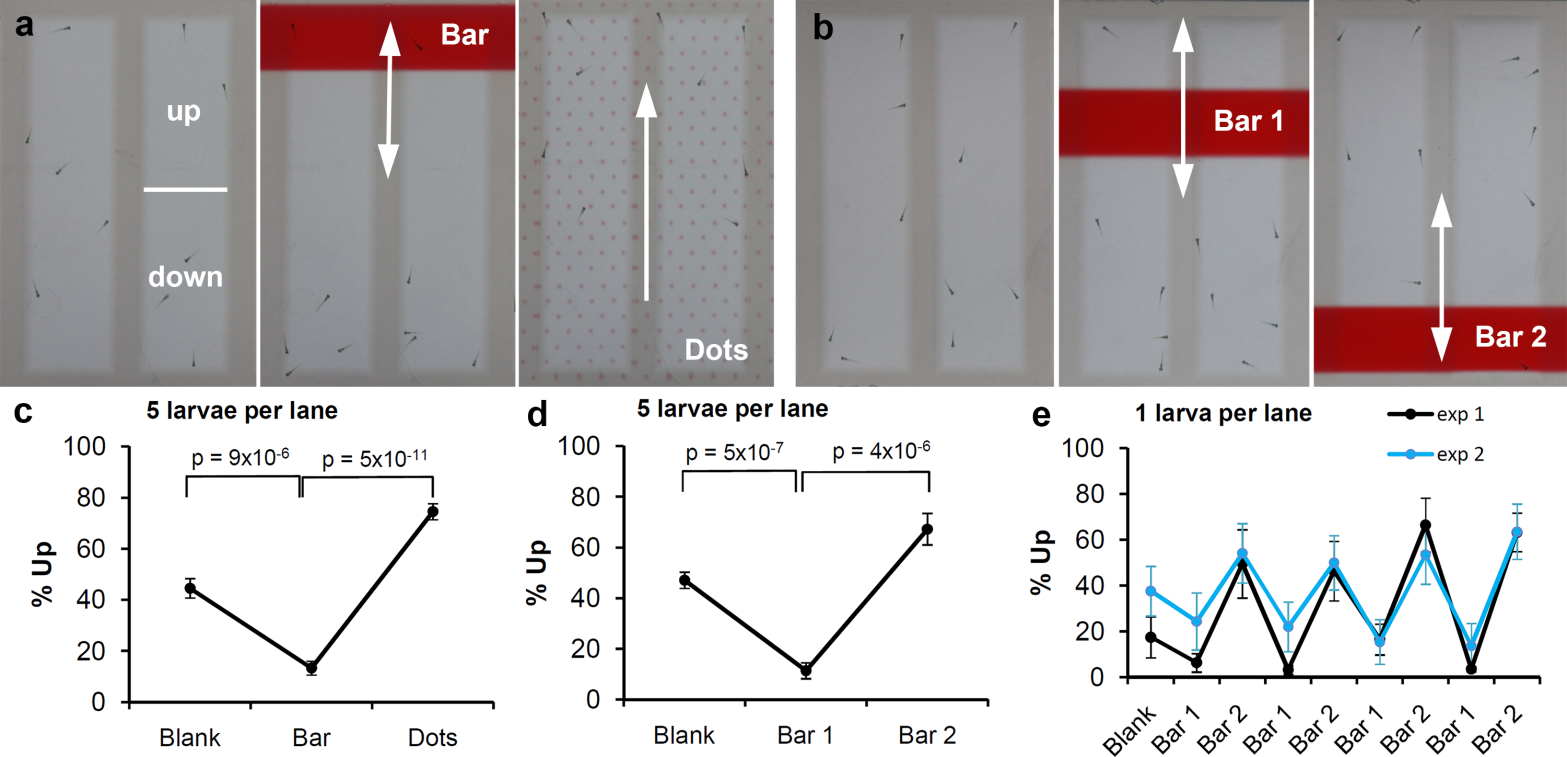
Visual stimuli in 5-lane plates. a) In the bar-dots assay, larvae are imaged for 15 min without visual stimuli, 15 min with a moving red bar and then 15 min with moving red dots. b) In the two-bar assay, larvae are imaged for 15 min without visual stimuli, 15 min with a moving red bar and then 15 min with a moving red bar in the opposite half of the plate. c) Measurements of larval location in the bar-dots assay. d) Measurements of larval location in the two-bar assay. e) Measurements of larval location using 1 larva per lane and a 4x repeated two-bar stimulus (10 min per bar). Differences in larval location were tested for significance using a two-tailed t-test with unequal variance and a Bonferroni correction for multiple comparisons when only pairwise comparisons were considered (c,d). A repeated measures ANOVA was performed to test the effect of stimulus on the %up for 4x repeated two-bar stimulus study. When sphericity was violated, a Greenhouse-Geisser correction was used (e). To assure that the data points are independent, even when using 5 larvae per lane, the data was analyzed on a per-lane basis (n = 10 lanes). All larvae were imaged at 5 dpf. % up = percentage of larval measurements in the upper half of a lane. The arrows indicate the movement of the visual stimuli.

Several new assays were developed to examine vision of 5 day-old zebrafish larvae in 5-lane plates (Fig 2). These assays expand on previous studies, which showed that zebrafish larvae avoid a moving red bar [22,23]. We first developed a ‘bar-dots assay’, which combines a red bar that moves up and down in the upper half of the plate with an array of red dots that move continuously up in the plate (Fig 2A). We found that the larvae avoid the area with the moving bar and swim in the same direction as the moving red dots (Fig 2C). The responses to the two visual stimuli differ significantly and this difference may be used as a robust measure of vision (p = 5x10^-11^, n = 10 lanes). In a second assay, called the ‘two-bar assay’, we showed a moving red bar in the upper half of the plate followed by a moving red bar in the lower half of the plate (Fig 2B). In this assay, larvae avoid the areas with the moving bars (Fig 2D) and larval locations differ significantly in response to the first and second bar (p = 4x10^-6^, n = 10 lanes). The bar-dots and two-bar assays described above were carried out using two 5-lane plates with 5 larvae per lane (50 larvae total). To examine if reliable results can be obtained with a lower number of larvae, we imaged two 5-lane plates with one larva per lane (10 larvae total). The same experiment was carried out twice without averaging the results, which allowed for an evaluation of the assay with only 10 larvae. Visual responses were examined in a ‘4x repeated two-bar assay’, which includes a moving red bar in the upper half of the plate (bar 1) and a moving red bar in the lower half of the plate (bar 2), shown to the larvae four times in a row (Fig 2E). We tested the effect of stimulus on %up in experiment 1 and experiment 2 by performing a repeated measures ANOVA. We found that in both cases the stimulus had a significant effect on % up at a critical value of p = 0.05 with a Greenhouse-Geisser correction for sphericity (experiment 1: F(2.955,26.594) = 11.667, p = 4.9 x 10^−5^ and experiment 2: F(3.655,32.893) = 3.622, p = 0.017). The measurements with 1 larva per lane are not as robust as the measurements with 5 larvae per lane, which may be expected since the experiments with 1 larva per lane contain 5x fewer observations than the experiments with 5 larvae per lane. Group effects with 5 larvae per lane are unlikely, since robust shoaling behaviors are only observed later in development [24,25]. S1 Table provides a summary of the assays at 5 dpf. In addition, we examined if the 4x repeated two-bar assay could be used to detect visual responses in younger larvae, at 4 dpf. In this experiment, two 5-lane plates were imaged using 5 larvae per lane and significant differences were observed when comparing the average of all bar 1 and bar 2 periods (p = 4x10^-8^, n = 10 lanes). Overall, the results in the 5-lane plates suggest that robust measurements of vision can be obtained at 4 and 5 dpf.

### Assays for visually-guided behaviors in 6-well plates

**Fig 3:**
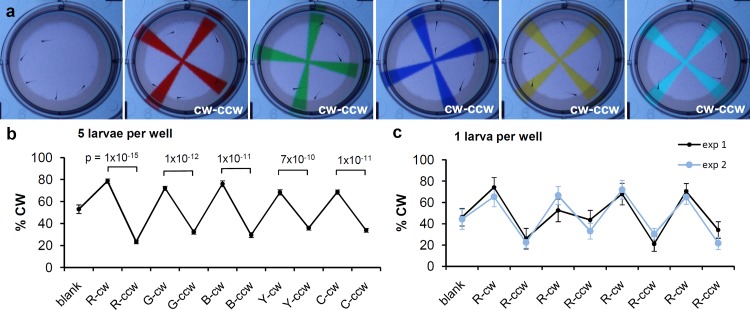
Visual stimuli in 6-well plates. a) View of a single well in a 6-well plate. b) Rotating cross assay using 5 larvae per well. c) Rotating red cross assay using 1 larva per well. In each color, the cross first rotates 10 minutes clockwise (cw) and then 10 minutes counter-clockwise (ccw). R, G, B, Y, C = red, green, blue, yellow, cyan. % CW = the percentage of measurements in which larvae display a clockwise orientation. Differences in the response to cw and ccw visual stimuli were tested for significance using a two-tailed t-test with unequal variance (n = 12 wells) and a Bonferroni correction for multiple comparisons when only pairwise comparisons were considered (a,b) A repeated measures ANOVA was performed to test the effect of stimulus on the % CW for 4x repeated two-bar stimulus study. When sphericity was violated, a Greenhouse-Geisser correction was used (c).

Novel ‘rotating cross’ assays were developed to examine vision of zebrafish larvae in 6-well plates (Fig 3). As a visual stimulus, we created a cross, which first rotates in a clockwise direction and then in a counter-clockwise direction. The rotating cross was colored red, green, blue, yellow, or cyan (Fig 3A) and is shown on a light gray background for optimal color separation during the image analysis. We found that 5 day-old zebrafish larvae display a clockwise orientation when the cross rotates clockwise and display a counter-clockwise orientation when the cross rotates counter-clockwise (Fig 3B). This change in larval orientation is robust in all colors (e.g. p = 1x10^-15^ in red, p = 1x10^-12^ in green, p = 1x10^-11^ in blue). To examine if reliable results can be obtained with a lower number of larvae, we imaged two 6-well plates with one larva per well (12 larvae total). The same experiment was carried out twice without averaging the results, which allowed for an evaluation of the assay with only 12 larvae. In these experiments, we showed a red cross rotating clockwise followed by a red cross rotating counter-clockwise and repeated this sequence four times (Fig 3C). We tested the effect of stimulus on clockwise orientation in experiment 1 and experiment 2 by performing a repeated measures ANOVA. We found that in both cases the stimulus had a significant effect on the percent clockwise at a critical value of p = 0.05 with a Greenhouse-Geisser correction for sphericity in experiment 2 (experiment 1: F(8,88) = 4.843, p = 5.4 x 10^−5^ and experiment 2: F(4.258,46.833) = 8.480, p = 2.2 x 10^−5^). S1 Table provides a summary of all assays at 5 dpf, using 10–60 larvae per experimental group. In general, the table indicates that one needs 12 larvae per experimental group to measure significant visual responses. However, the assays become substantially more sensitive using 50–60 larvae per experimental group. We also examined if a 4x repeated rotating red cross assay could be used to detect visual responses in younger larvae, at 4 dpf. In this experiment, two 6-well plates were imaged using 5 larvae per well and significant differences were observed when comparing the average of all clockwise and counter-clockwise periods (p = 4x10^-11^, n = 12 wells). Since the larvae are located only a few millimeters away from the computer screen, the larvae will view the visual stimuli differently than a distant observer. For example, an approaching arm of the rotating cross and an approaching bar in the multilane plates may look similar from the viewpoint of a larva. The main differences between the assays are the measurement of larval location (multilane plates) vs. larval orientation (6-well plates) and the shape of the swimming area. In multilane plates, larvae typically swim away from the moving bar until they reach a corner at the end of the lane. In contrast, larvae in 6-well plates do not have an endpoint, as they can continue to swim around in the well. Based on the results in 6-well plates, we conclude that robust measurements of vision can be obtained with the rotating cross assay using either 5 larvae per well or using 1 larva per well with repeated visual stimuli.

### UV-induced visual defects examined in 5-lane plates

**Fig 4:**
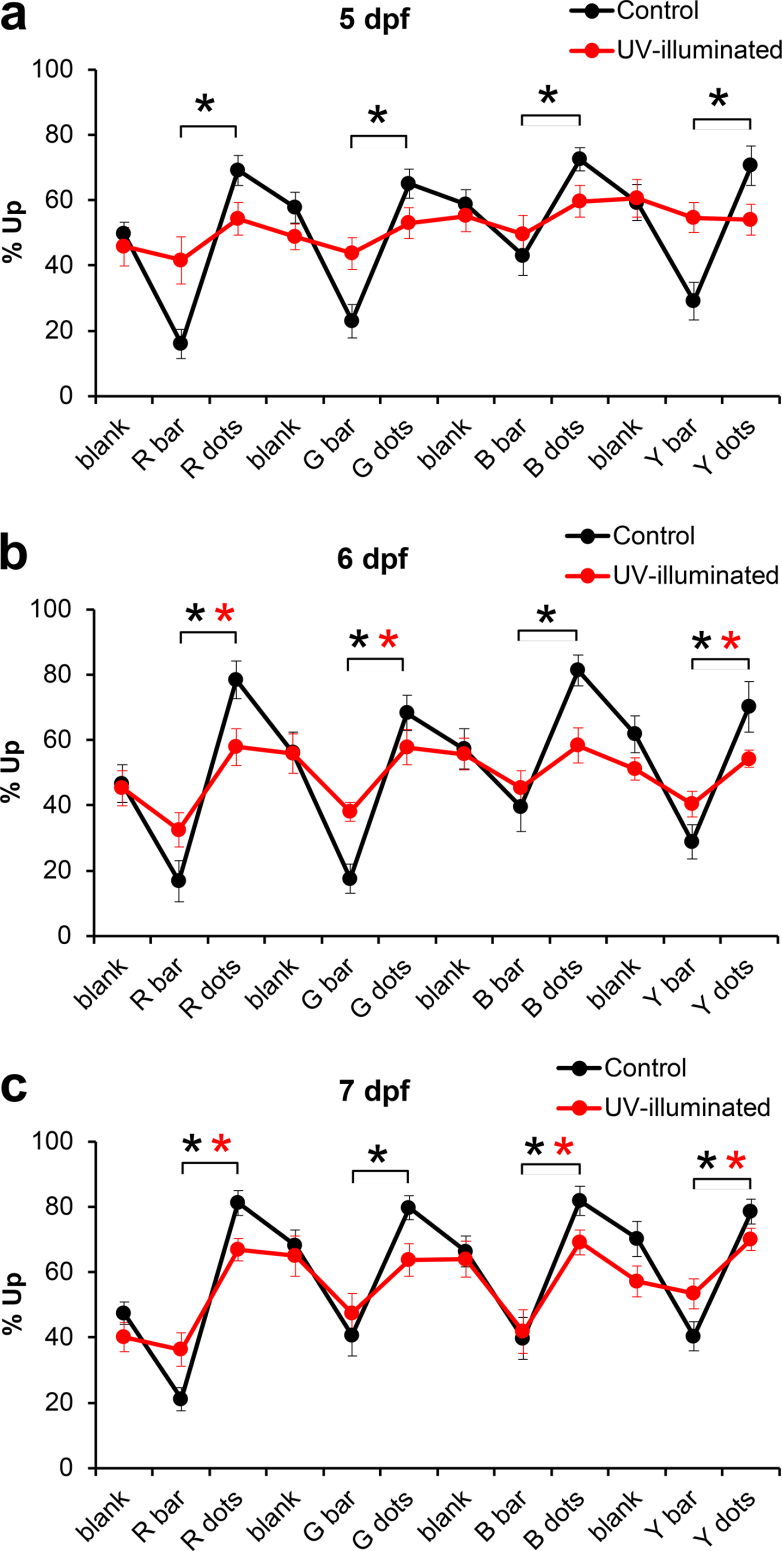
Effects of UV illumination examined in 5-lane plates. a) UV-illuminated larvae display a strongly reduced visual response at 5 dpf. b) Partial recovery of the response to visual stimuli at 6 dpf. c) Near complete recovery of the response to visual stimuli at 7 dpf. R, G, B, Y = red, green, blue, yellow. Black * = p<0.01, bar vs. dots in control larvae (exposed to tricaine without UV illumination). Red * = p<0.01 bar vs. dots in UV-illuminated larvae (two-tailed t-test with unequal variance, n = 10 lanes). The 5-lane plates contained 5 larvae per lane.

The 5-lane plates were used to examine visual defects and the recovery from these defects. Visual defects were induced at 5 dpf by UV illumination, using a protocol adapted from Meyers et al. (2012) [19]. The larvae were anesthetized with tricaine during a 5 min UV-illumination period and tricaine-treated larvae, without UV illumination, were used as controls. Larvae from both groups were washed in egg water and allowed to recover from the anesthetic for 2 hours prior to the behavioral assays. Visual defects were first examined in 5-lane plates, with 5 larvae per lane, using a modified bar-dots assay carried out in red, green, blue and yellow (Fig 4). The tricaine-treated control larvae displayed clear visual responses in red, green, blue and yellow at 5, 6 and 7 days post-fertilization (dpf). In each of these cases, we confirmed that the response to the bar was significantly different from the response to the dots (p<0.01, n = 10 lanes). UV-illuminated larvae did not respond to visual stimuli in any color at 5 dpf (Fig 4A), consistent with prior studies showing a destruction of photoreceptor cells by high-intensity light [19]. At 6 dpf, the UV-illuminated larvae showed a gradual recovery of vision, i.e. the larvae displayed a significant visual response in red, green and yellow (Fig 4B). These responses are lower than the responses observed in the controls (p<0.01 in all colors), indicating that the recovery of vision is incomplete at 6 dpf. At 7 dpf, the UV-illuminated larvae show a significant visual response in red, blue and yellow (Fig 4C). The visual response in red, green and yellow is suppressed in the UV-illuminated larvae compared to the tricaine-treated controls (p<0.01, p<0.05, p<0.01, respectively) indicating that the recovery of vision still incomplete at 7 dpf.

### UV-induced visual defects examined in 6-well plates

**Fig 5:**
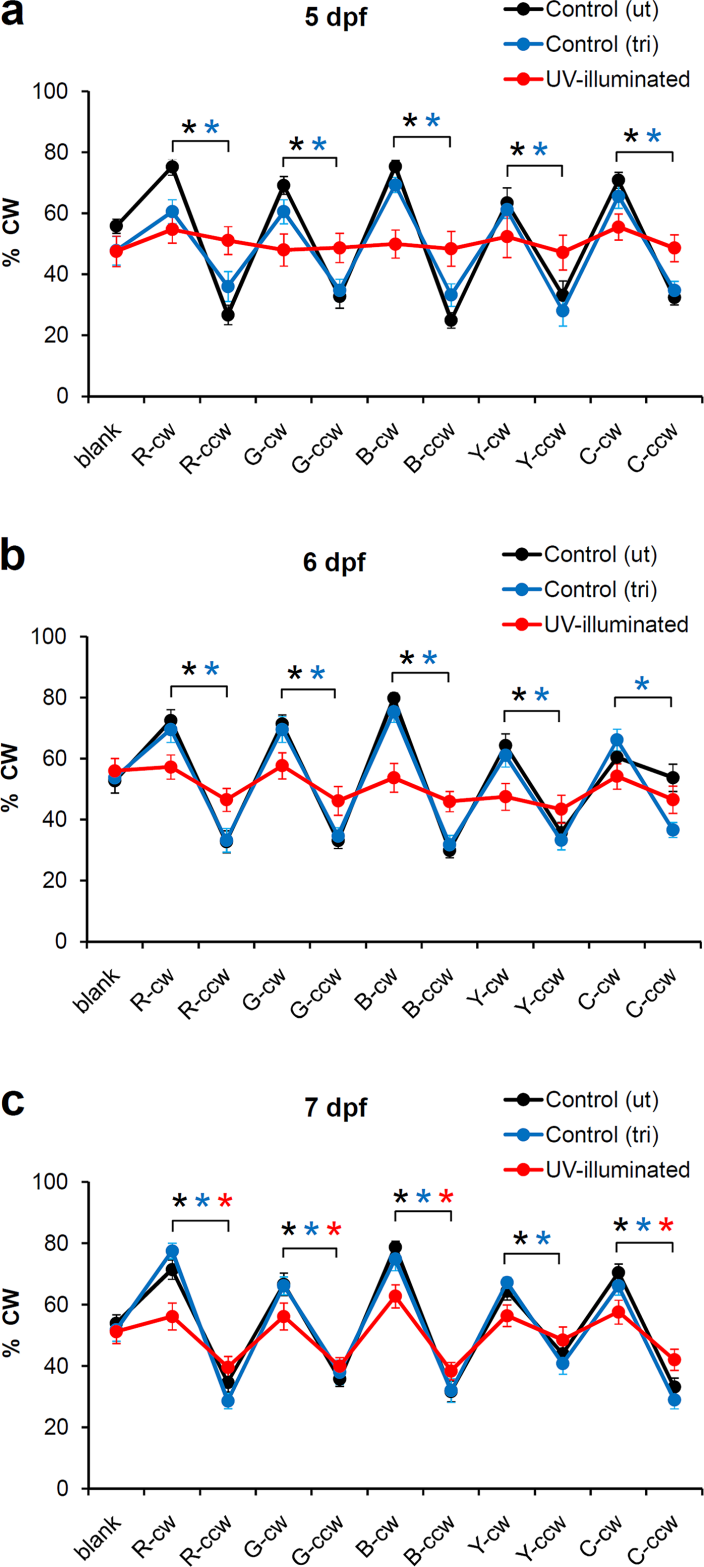
Effects of UV illumination examined in 6-well plates. a) UV-illuminated larvae do not show a significant response to visual stimuli at 5 dpf. b) Similarly, UV-illuminated larvae do not show a significant response to visual stimuli at 6 dpf. c) Near complete recovery of vision at 7 dpf. Black * = p<0.01, cw vs. ccw in unexposed control larvae. Blue * = p<0.01, cw vs. ccw in tricaine-exposed control larvae. Red * = p<0.01, cw vs. ccw in UV-illuminated larvae (two-tailed t-test with unequal variance, n = 12 wells per experimental group). R,G,B,Y,C = rotating cross in red, green, blue, yellow and cyan. cw = clockwise rotation of cross. ccw = counter-clockwise rotation of cross. The 6-well plates contained 5 larvae per well.

We used the rotating-cross assay in 6-well plates to examine UV-induced visual defects and the recovery from these defects. The rotating cross assay was carried out with 5 larvae per well as shown in Fig 3B. Untreated and tricaine-treated control larvae showed a robust response to all colors at 5, 6, and 7 dpf (Fig 5). In each color, the response to the cross rotating clockwise was significantly different from the response to the cross rotating counter-clockwise (p<0.01, n = 12 wells). UV-illuminated larvae did not display a significant response to the visual stimuli in any color at 5 dpf (Fig 5A). To examine if UV illumination affects motor performance, we analyzed the average swim speed during the first 20 minutes of the experiment without visual stimuli. We found that UV illumination does not have a significant effect on swim speed (6 mm/min in the tricaine controls (N = 11 wells, 55 larvae, SEM = 1.82) vs. 5 mm/min in the UV exposure group (N = 10 wells, 50 larvae, SEM = 0.81), p = 0.53, two tailed t-test). Thus, the UV-treatment affects the response to visual stimuli, but does not affect swim speed in 5 dpf larvae. UV-treated larvae did not display a significant response to visual stimuli at 6 dpf. However, a partial recovery of vision was observed at 7 dpf. The 7 dpf larvae displayed a significant visual response in red, green, blue and cyan, but not in yellow (Fig 5C). The response in red, blue, yellow and cyan was reduced in the UV-illuminated larvae as compared to the tricaine-treated controls (p<0.01, p<0.05, p<0.05, p<0.01, respectively), indicating that the recovery of vision still incomplete at 7 dpf. Based on these results, we conclude that the rotating cross assay is an efficient tool to examine UV-induced visual defects and the gradual recovery from these defects.

### Visual defects induced by pde6c knockdown

**Fig 6:**
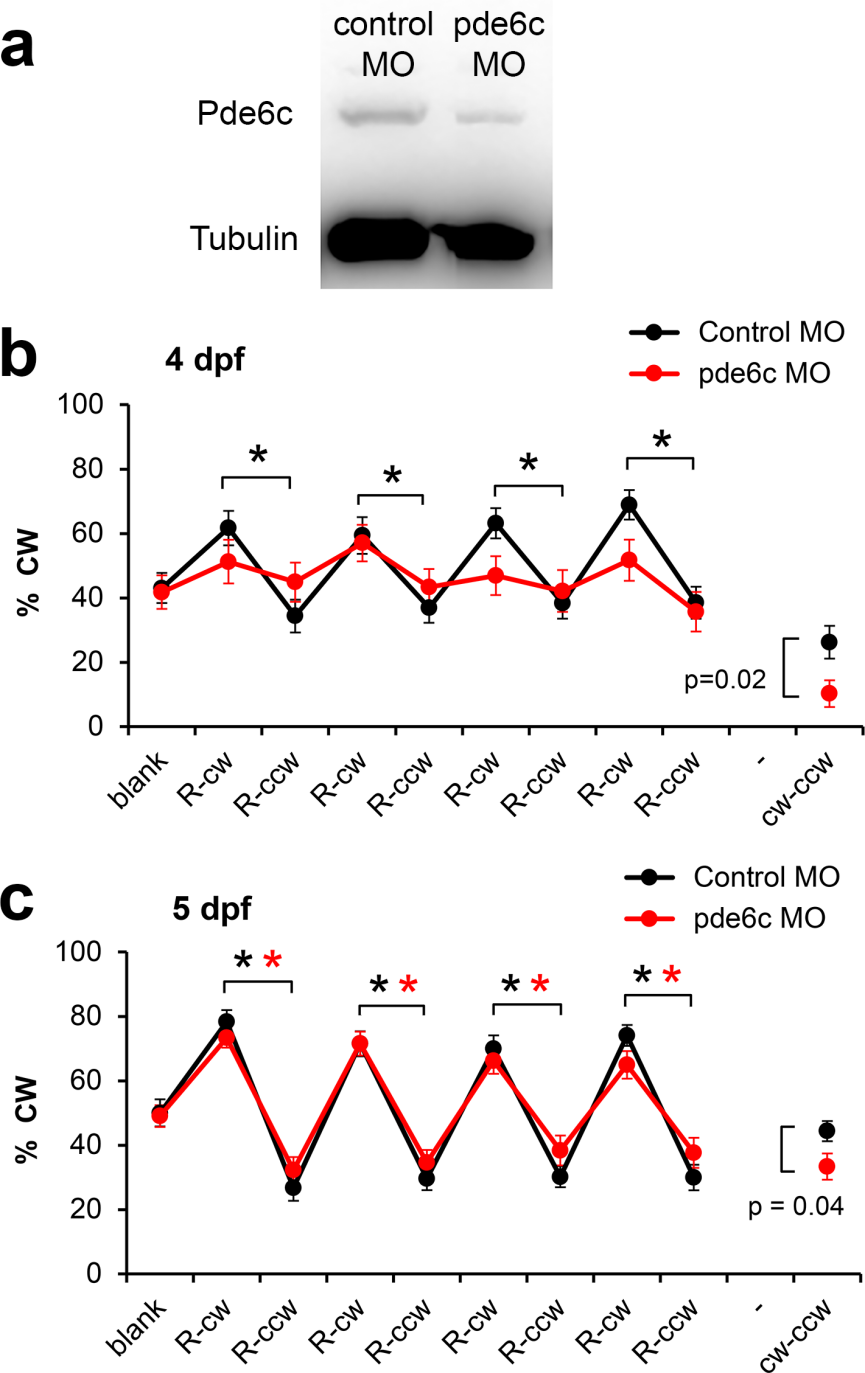
Visual defects after pde6c knockdown. a) Effects of pde6c knockdown at 4 dpf. Pde6c protein levels were suppressed by pde6c morpholino oligonucleotides (MO) as shown by Western blotting. b) The 4 dpf control larvae displayed a significant visual response to the 4x repeated rotating red cross. In contrast, the pde6c morpholino-injected larvae did not show a significant response to the visual stimuli. c) Effects of pde6c knockdown at 5 dpf. The control larvae and pde6c larvae display a robust response to the rotating red cross at 5 dpf. Black * = p<0.01, cw vs. ccw in control larvae. Red * = p<0.01, cw vs. ccw in pde6c larvae (two-tailed t-test with unequal variance for pairwise comparisons, n = 35 wells in the control group, n = 33 wells in the pde6c group). R = red cross, cw = clockwise rotation of cross, ccw = counter-clockwise rotation of cross. The average response to clockwise stimuli minus the average response to counter-clockwise stimuli is shown as a separate point in the graph (cw-ccw). These averages are significantly different between the control and pde6c groups at a 95% confidence limit (p<0.05). The 6-well plates contained 1 larva per well.

A specific defect in visual function was induced by morpholino-mediated knockdown of the phosphodiesterase Pde6c, a key signal transduction protein in retinal cone cells [26]. Previous studies have shown that low morpholino concentrations are effective in the zebrafish retina at 4 dpf, but not at 5 dpf, which allows one to examine a loss and recovery of visual function [27]. We examined Pde6c protein levels in 4 dpf larvae by Western blotting (Fig 6A). We found Pde6c protein levels are reduced 49% in pde6c morpholino-injected larvae as compared to control morpholino-injected larvae (ratiometric measurements with Tubulin protein levels in the denominator). These results indicate that the pde6c morpholino is effective in knocking down Pde6c levels. Visual defects were examined using 1 larva per well and the 4x repeated rotating red cross assay, starting at 4 dpf. Larvae injected with control morpholinos displayed a robust response to each of the four pairs of rotating red crosses at 4 dpf [F(4.468,151.914) = 8.007, p = 2.7x10^-6^, with Greenhouse-Geisser correction for sphericity violation] and 5 dpf [F(5.787,196.756) = 37.813, p = 1.1x10^-29^, with Greenhouse-Geisser correction for sphericity violation] (Fig 6). In each pair of crosses, the response to the cross rotating clockwise was significantly different from the response to the cross rotating counter-clockwise (p<0.01). The pde6c morpholino-injected larvae did not display a significant visual response at 4 dpf [F(4.983,159.454) = 1.665, p = 0.146, with Greenhouse-Geisser correction for sphericity violation]. To examine if pde6c morpholinos affect motor performance, we analyzed the average swim speed during the first 20 minutes of the experiment without visual stimuli. We found that the pde6c morpholino does not have a significant effect on swim speed (17 mm/min with the control morpholino (N = 36 wells, 36 larvae, SEM = 3.00) vs. 18 mm/min with the pde6c morpholino (N = 34 wells, 34 larvae, SEM = 3.64), p = 0.76, two-tailed t-test). Thus, pde6c morpholino-injected larvae display normal swim speeds at 4 dpf, but don’t respond to visual stimuli. At 5 dpf, the pde6c morpholino-injected larvae display a near complete recovery of vision [F(4.925,157.589) = 21.992, p = 1.6x10^-16^]. Based on these results, we conclude that the 4x repeated rotating red cross assay can be used to examine morpholino-induced visual defects and the recovery from these defects.

## Discussion

In the current study, we introduce new software and algorithms for automated analyses of visually-guided behaviors and present novel assays for measuring vertebrate vision in microplates. In addition, we used the developed methodologies to detect visual defects and the gradual recovery from these defects.

The software was written as an open-source ImageJ macro and was designed for automated analyses of microplates with multiple visual stimuli that change over time. With this macro, it is possible to measure the location and orientation of larvae in a large field of view (4 microplates per image), while various red, green, blue, yellow or cyan objects are presented to the larvae. The developed software builds on a previously developed ImageJ macro that we developed for automated analyses of zebrafish behavior in microplates [22,23]. However, the previously developed macro was not suitable for automated analyses of multi-color experiments. Both the previously developed macro and the new macro presented in this study allow for the analysis of microplates while objects are displayed to zebrafish larvae, which is not feasible using any commercially-available imaging system.

The developed assays in 5-lane plates build on our previous studies showing that zebrafish larvae avoid a moving red bar [22,23]. We found that the red bar assay can be substantially improved by including additional visual stimuli. In the ‘bar-dots’ assay, 5 dpf larvae first swim away from the moving bar into the lower half of the lane, and then swim in the same direction as the moving dots into the upper half of the lane. These changes in larval location are robust and provide a reliable measure of vision. Similarly, the two-bar assay and 4x repeated two-bar assay can be used to drive 5 dpf larvae up and down a lane. In the ‘rotating cross’ assay in 6-well plates, larvae display a clockwise orientation when the cross rotates clockwise, and a counter-clockwise orientation when the cross rotates counter-clockwise. In this assay, larvae respond well to all colors tested, including red, green, blue, yellow and cyan on a light background. The rotating cross assay is robust and can be used to evaluate vision when a limited number of larvae are available. The response of zebrafish larvae to moving objects may be used in nature to avoid predators and capture prey, depending on the size of the object [28]. Thus, the larvae may view the moving bar as a looming predator and the moving dots as prey. Zebrafish larvae are also known to maintain a fixed location in a water stream, which is the basis of an orienting behavior in the optomotor response [29]. Similarly, the observed response to the rotating cross may reflect an optomotor-related orienting behavior. The 4x repeated two-bar assay and the 4x repeated rotating cross assays were used to reliably measure visual responses early in development, at 4 dpf. Thus, larvae displayed a visual response to the moving bar and rotating cross at same developmental stage as the optokinetic response, which can be reliably measured at 4 dpf [7,8] and 2–3 days earlier than the optomotor response, which can be reliably measured at 6–7 dpf [11,13].

The developed methodologies were used to measure a loss and recovery of vision. Visual defects were induced at 5 dpf by UV-illumination, using an adapted protocol from Meyers et al. [19]. These UV-illuminated larvae did not display a significant response to visual stimuli in a multicolor bar-dots assay or a multicolor rotating cross assay. However, vision gradually recovers in 1–2 days after UV-illumination. This rapid recovery of vision is consistent with previous studies showing retinal regeneration following light-induced ablation of photoreceptor cells [19] and a rapid recovery of vision after cone photoreceptor ablation using a nitroreductase system [30]. In the rotating cross assay, 7dpf larvae displayed a recovery of visual responses in red, green, blue and cyan, but not in yellow. The suppressed response in yellow could indicate a persistent UV-induced defect in blue photoreceptor cells, i.e. the yellow stimuli and light background are likely indistinguishable without seeing blue. Apart from the UV illumination, we also used *pde6c* morpholinos to temporarily block photoreceptor function.

Pde6c is a signaling protein in cone photoreceptors and mutations in the *pde6c* gene lead to visual defects in both zebrafish and humans [26]. Zebrafish larvae express *pde6c* in cone photoreceptors and homozygous *pde6c* mutant larvae display a rapid degeneration of cone photoreceptors during early larval stages [26,31]. Electroretinograms and optokinetic assays revealed that the *pde6c* mutant larvae are blind, consistent with the idea that early larval vision depends on cone photoreceptors. Rods do not degenerate in *pde6c* mutant fish and an optokinetic response can be observed at 3 weeks post-fertilization in low-light conditions [31]. We chose to use morpholinos, instead of mutant fish lines, based on previous studies showing that morpholino concentrations can be adjusted to affect the zebrafish retina at 4 dpf, but not at 5 dpf [27]. While morpholinos can have off-target effects [32], the *pde6c* morpholinos effectively suppressed Pde6c protein levels, did not affect baseline motor performance, and induced visual defects at 4 dpf consistent with the mutant phenotype. In addition, we found that visual responses recover at 5 dpf, suggesting that *pde6c* morpholinos may be used to examine both the loss and recovery of visual function.

Based on the obtained results, we conclude that the developed assays are efficient in measuring the loss and recovery of visual function. The developed assays are the first to measure the loss and recovery of vertebrate vision in microplates and provide an efficient platform to evaluate novel treatments of visual impairment.

## Supporting information

S1 Table Assays for visually-guided behaviors in 5 day old zebrafish larvae. Behavioral assays were compared using 2 plates per experiment. In experiments with 1 larva per lane or well, we examined 2 plates per experiment (n = 10 lanes or 12 wells), repeated this in a second experiment, and presented the average p-values of the two experiments. P = two-tailed t-test, unequal variance, with a Bonferroni correction for multiple comparisons (2, 2, 3, 1, 3 in experiment 1–5). To maintain independence of measurements in lanes or wells containing multiple larvae, the statistical analyses were carried out on a per-well basis (n = number of wells or lanes). cw = clockwise, ccw = counter-clockwise.
(DOCX)

S1 File Red bar–Red dots powerpoint. (PPTX)

S2 File Red bar–Red bar powerpoint. (PPTX)

S3 File Red bar–Red bar 4x repeated powerpoint. (PPTX)

S4 File Blank–Bar–dots RGBY powerpoint. (PPTX)

S5 File Rotating cross CW-CCW RGBYC powerpoint. (PPTX)

S6 File Rotation red cross CW-CCW 4x repeated powerpoint. (PPTX)

S7 File Zebrafish behavior image analysis ImageJ macro 26bc. (TXT)

S8 File Zebrafish behavior analysis excel template 26b. (XLSB)
