# Supplementary material for: The loss and recovery of vertebrate vision examined in microplates
Source: PLoS One. 2017 Aug 17;12(8):e0183414. doi: 10.1371/journal.pone.0183414 (PMC5560659; doi:10.1371/journal.pone.0183414)
Supplement: S1 Table — Behavioral assays were compared using 2 plates per experiment. In experiments with 1 larva per lane or well, we examined 2 plates per experiment (n = 10 lanes or 12 wells), repeated this in a second experiment, and presented the average p-values of the two experiments. P = two-tailed t-test, unequal variance, with a Bonferroni correction for multiple comparisons (2, 2, 3, 1, 3 in experiment 1–5). To maintain independence of measurements in lanes or wells containing multiple larvae, the statistical analyses were carried out on a per-well basis (n = number of wells or lanes). cw = clockwise, ccw = counter-clockwise. (DOCX) [file pone.0183414.s001.docx]

|  | **Plate** | **#**  **plates** | **Larvae per well** | **Visual stimuli** | **Color** | **Comparison** | **P (n=wells)** | **Total larvae** |
| --- | --- | --- | --- | --- | --- | --- | --- | --- |
| 1. | 5-lane | 2 | 5 | bar, dots | red | blank vs. bar | 9x10^-6^ (10) | 50 |
|  | ˶ | 2 | 5 | ˶ | red | bar vs. dots | 5x10^-11^ (10) | 50 |
| 2. | 5-lane | 2 | 5 | bar 1, bar 2 | red | blank vs. bar | 5x10^-7^ (10) | 50 |
|  | ˶ | 2 | 5 | ˶ | red | bar 1 vs. bar 2 | 4x10^-6^ (10) | 50 |
| 3. | 5-lane | 2 | 1 | bar 1, bar 2 (4x) | red | blank vs. bar | 1 (10) | **10** |
|  | ˶ | 2 | 1 | ˶ | red | bar 1 vs. bar 2 | 0.2 (10) | **10** |
|  | ˶ | 2 | 1 | ˶ | red | bar vs. bar (4x) | 0.0101 (10) | **10** |
| 4. | 6-well | 2 | 5 | cross cw, ccw | red | cw vs. ccw | 1x10^-15^ (12) | 60 |
|  | ˶ | 2 | 5 | ˶ | green | cw vs. ccw | 1x10^-12^ (12) | 60 |
|  | ˶ | 2 | 5 | ˶ | blue | cw vs. ccw | 1x10^-11^ (12) | 60 |
|  | ˶ | 2 | 5 | ˶ | yellow | cw vs. ccw | 7x10^-10^ (12) | 60 |
|  | ˶ | 2 | 5 | ˶ | cyan | cw vs. ccw | 1x10^-11^ (12) | 60 |
| 5. | 6-well | 2 | 1 | cross cw, ccw (4x) | red | blank vs. cw | 0.24 (12) | **12** |
|  | ˶ | 2 | 1 | ˶ | red | cw vs. ccw | 4x10^-3^ (12) | **12** |
|  | ˶ | 2 | 1 | ˶ | red | cw vs. ccw (4x) | 8x10^-4^ (12) | **12** |
