# Supplementary figures and images for: The loss and recovery of vertebrate vision examined in microplates
Source: PLoS One. 2017 Aug 17;12(8):e0183414. doi: 10.1371/journal.pone.0183414 (PMC5560659; doi:10.1371/journal.pone.0183414)

## Slide 1
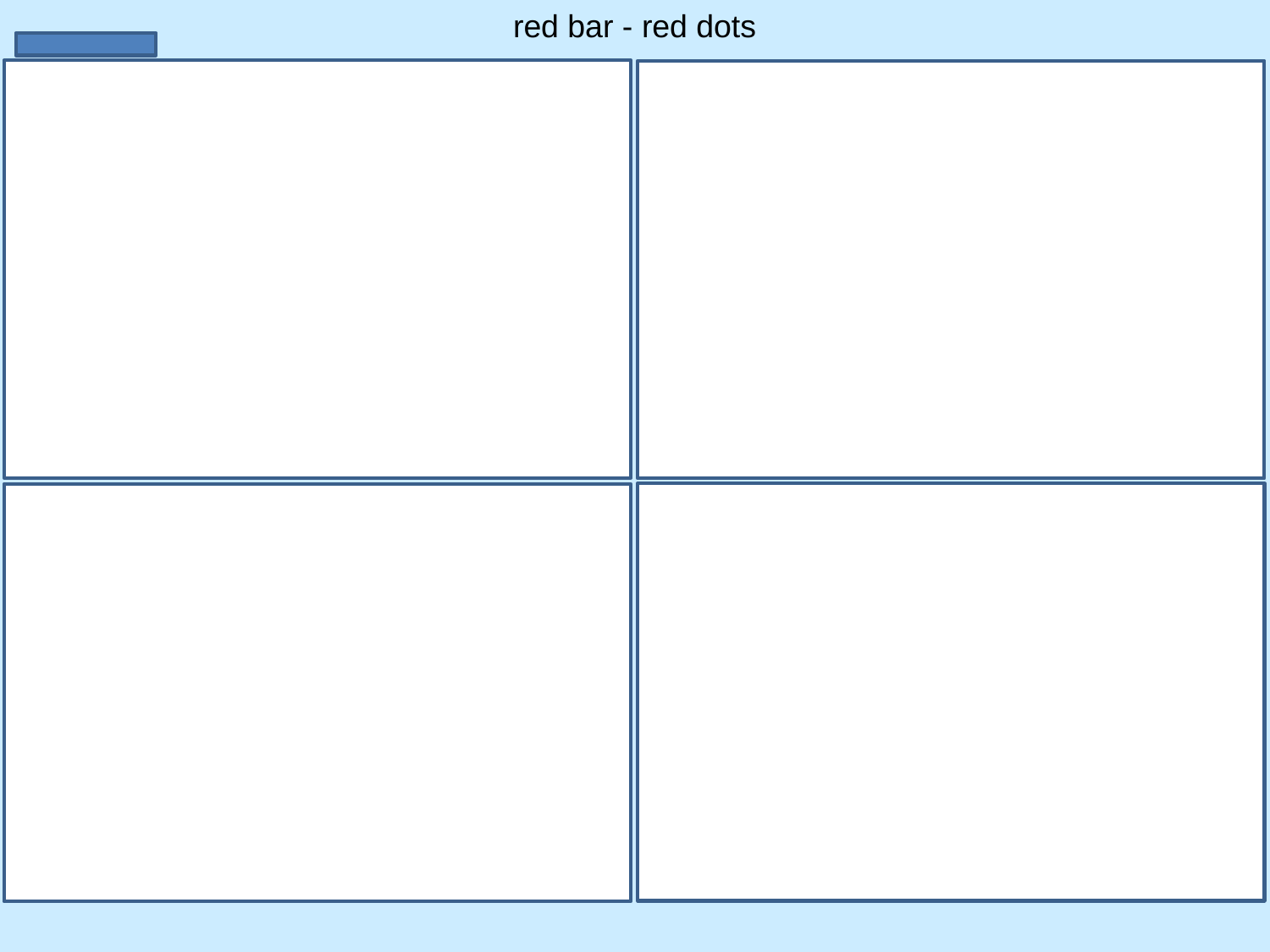

red bar - red dots

## Slide 2
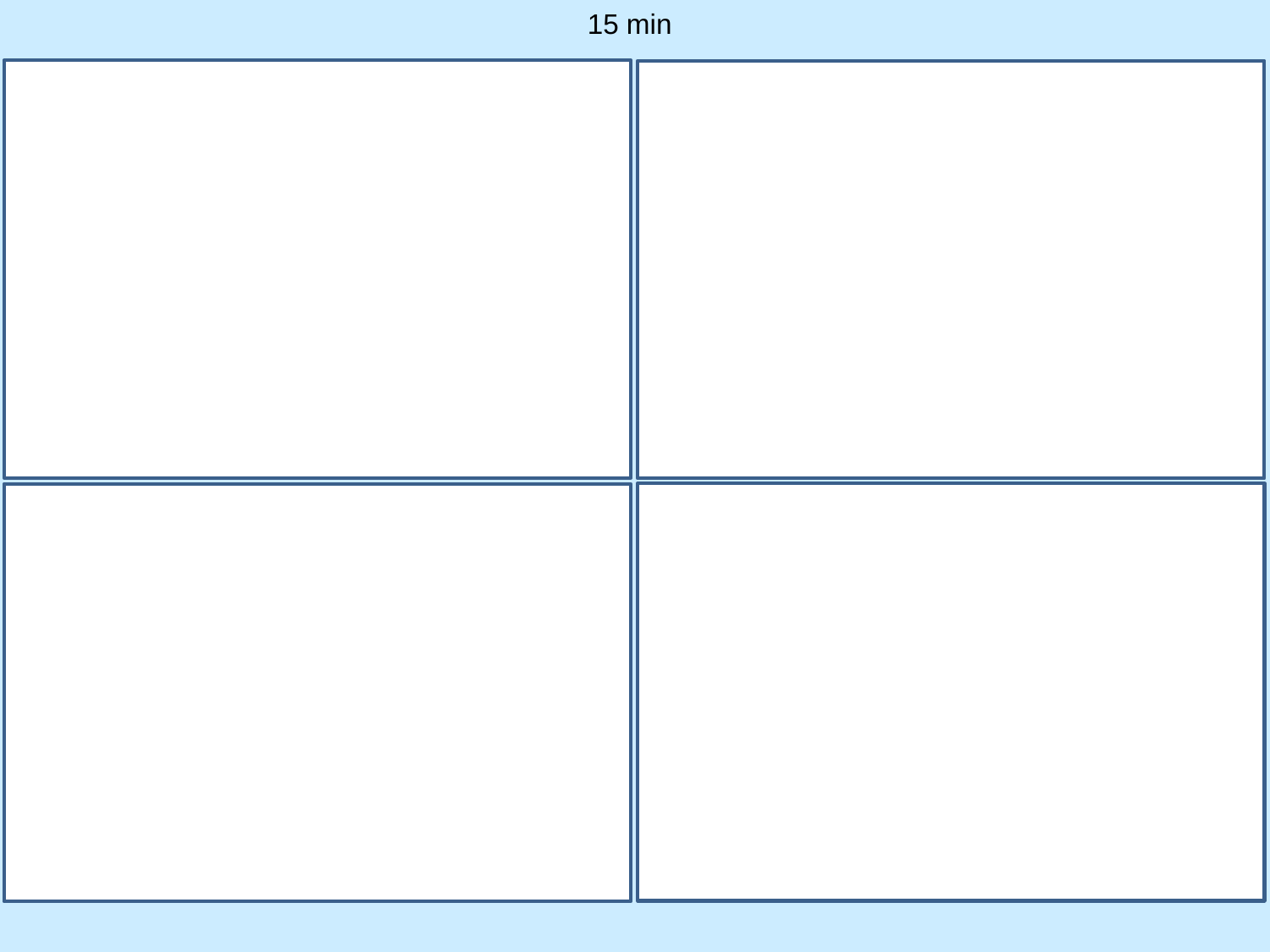

15 min

## Slide 3
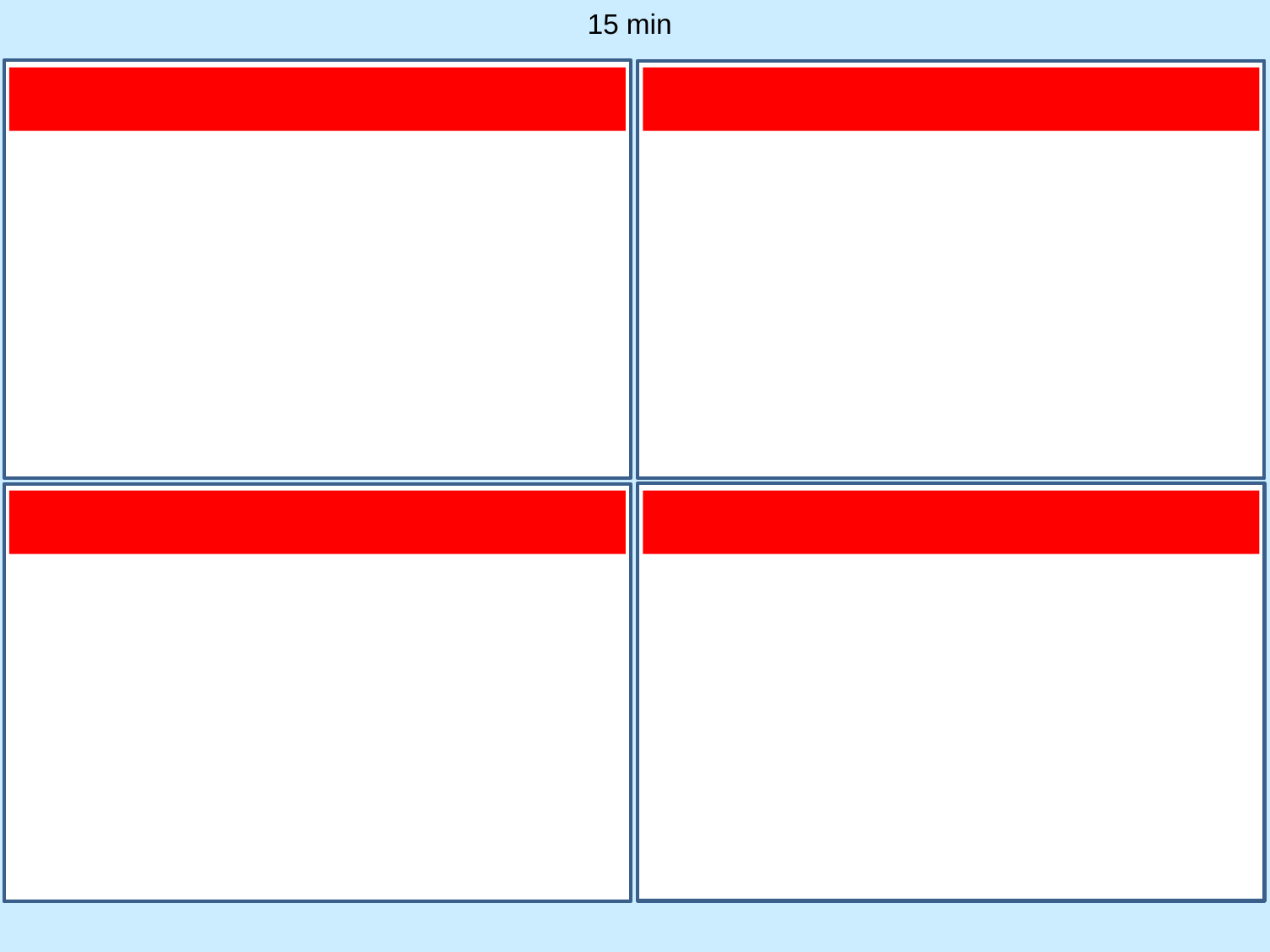

15 min

## Slide 4
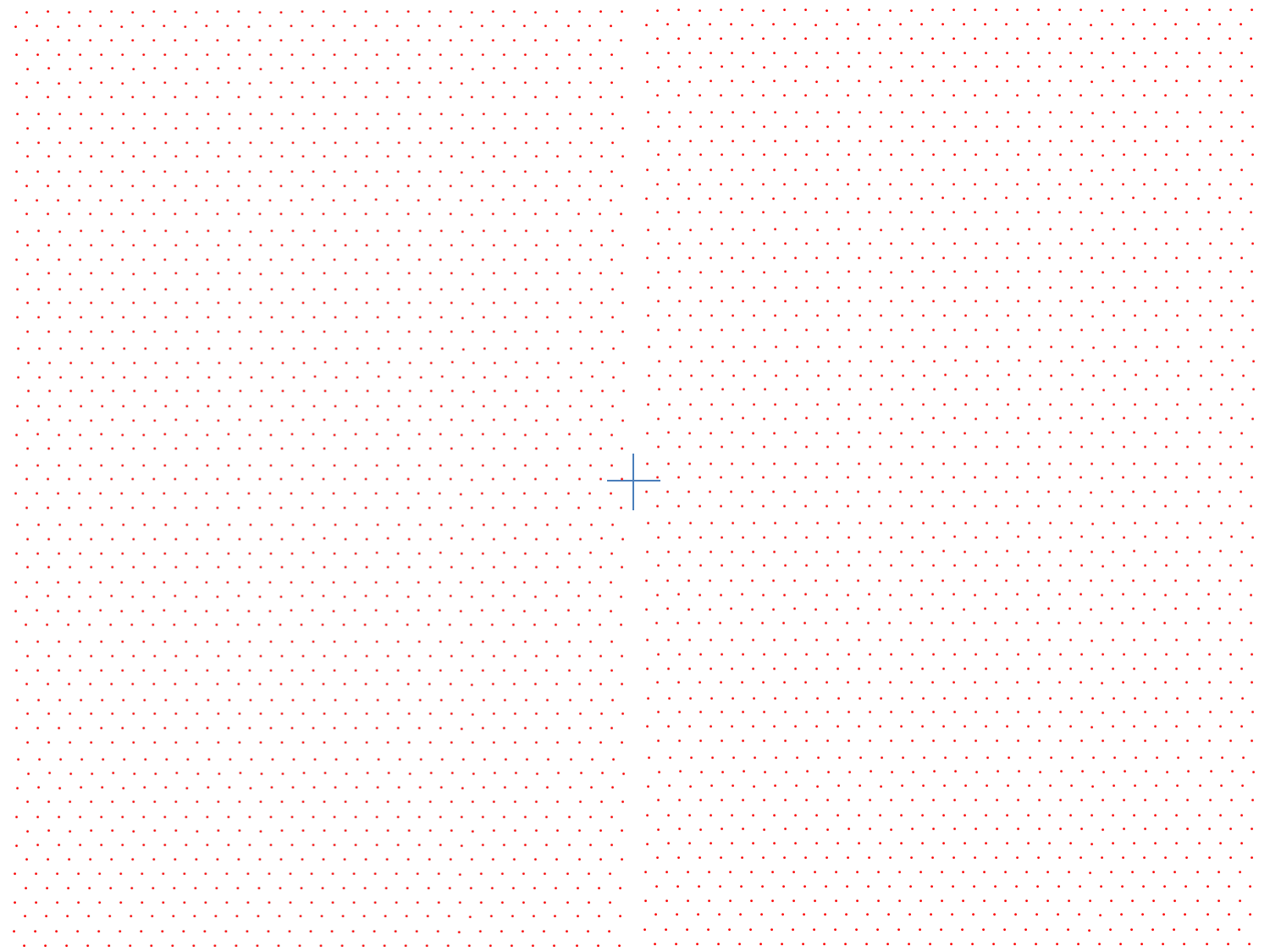

Supplement: S1 File — (PPTX) [file pone.0183414.s002.pptx]

## Slide 1
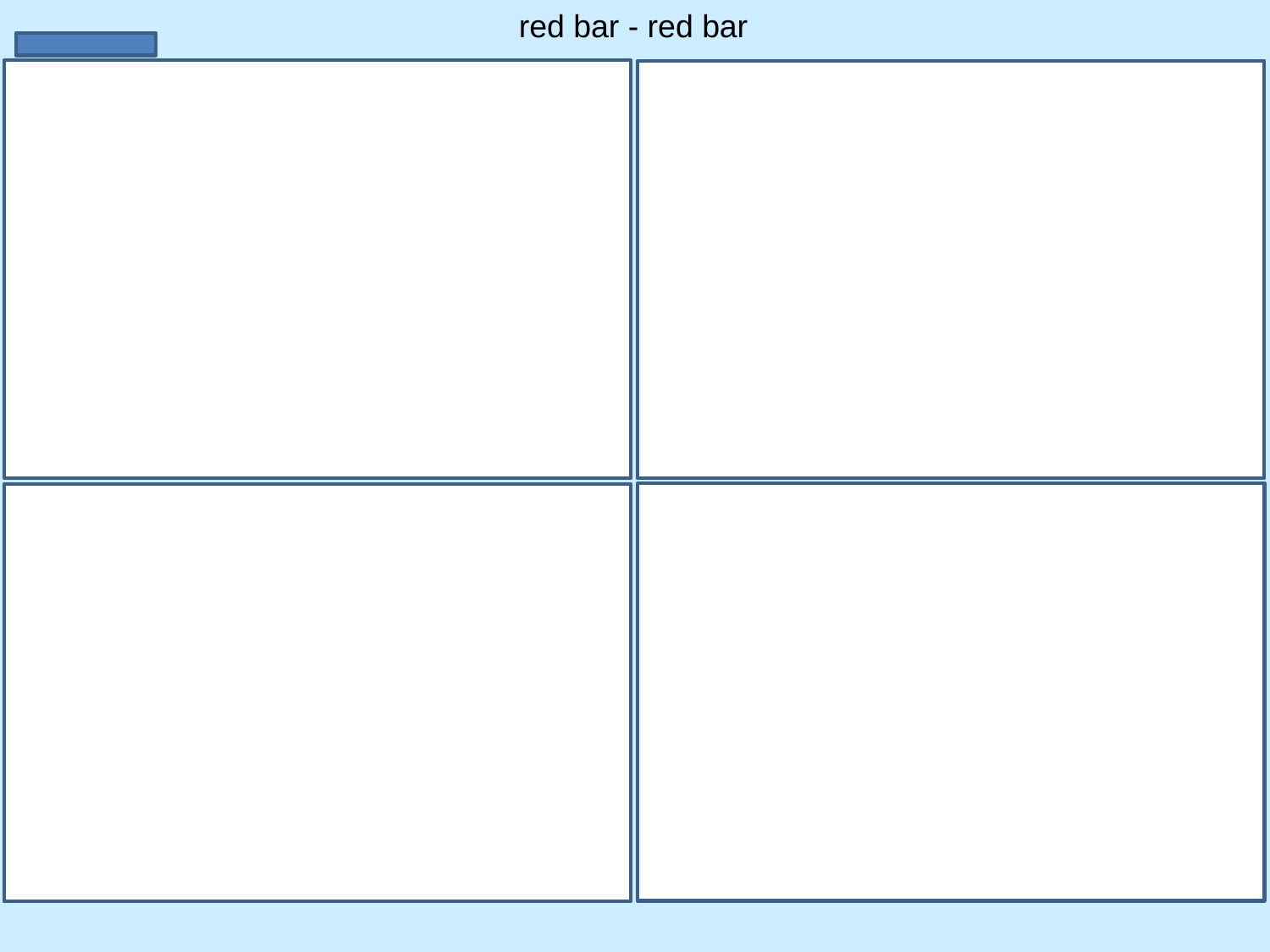

red bar - red bar

## Slide 2
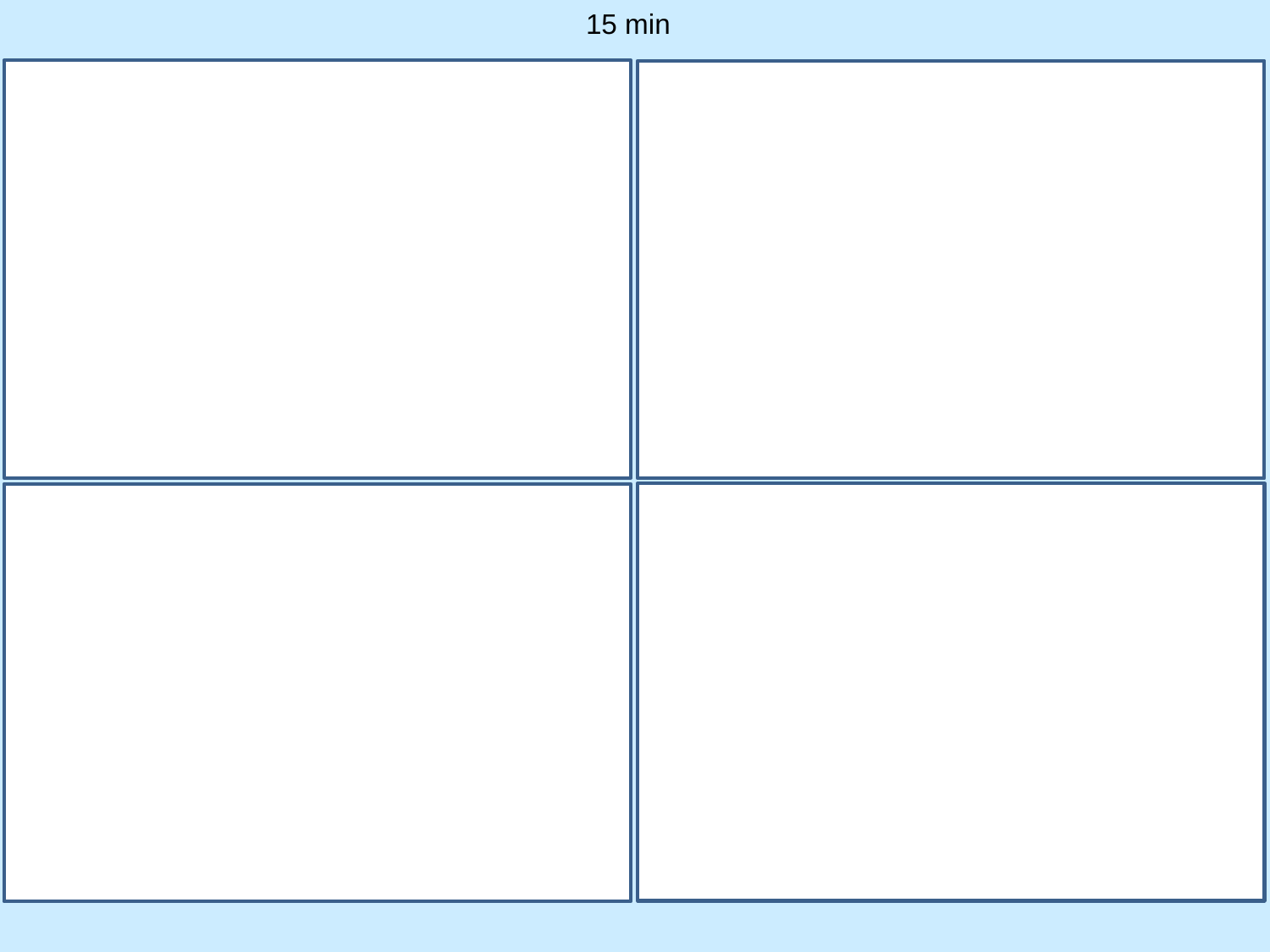

15 min

## Slide 3
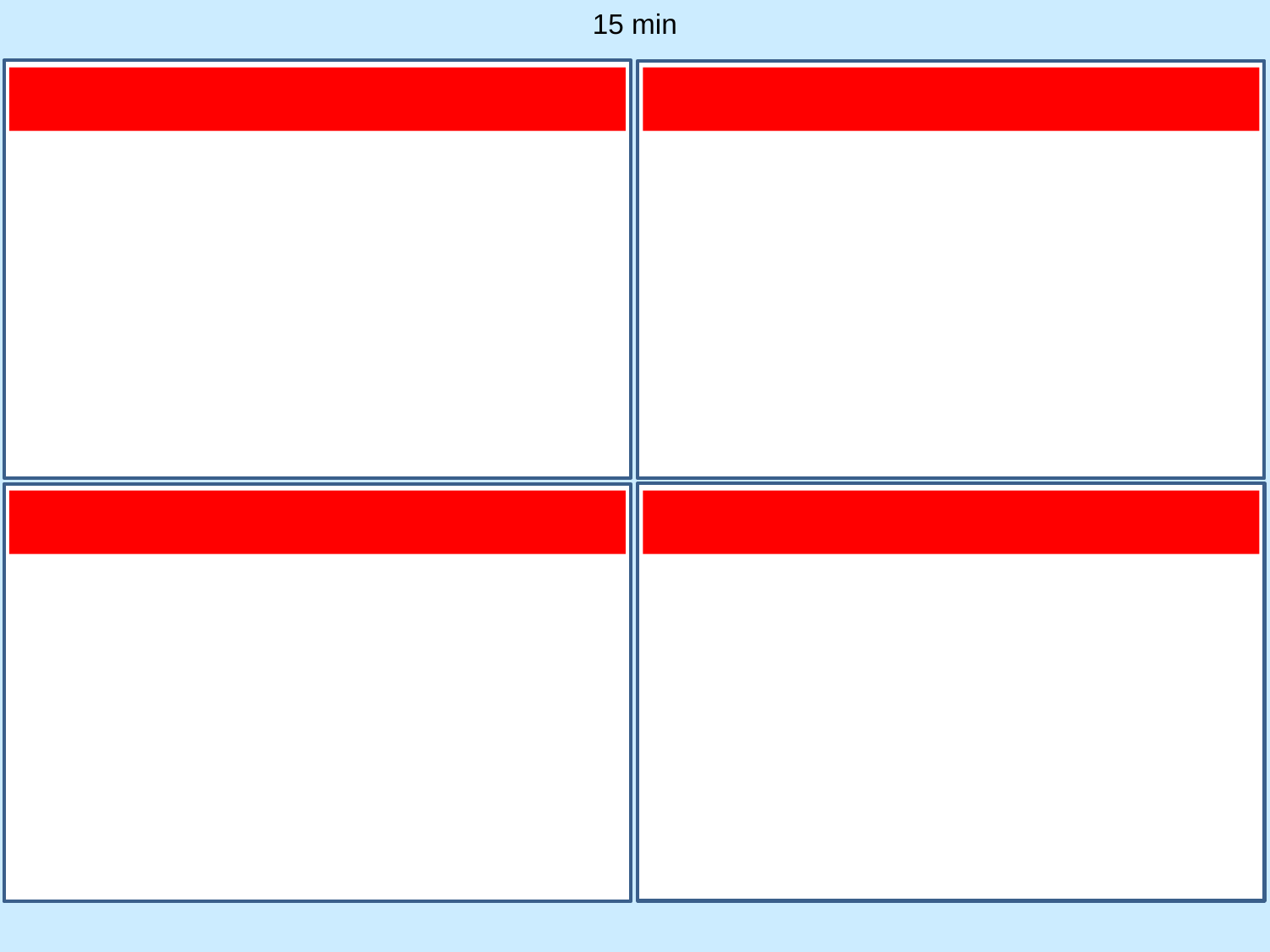

15 min

## Slide 4
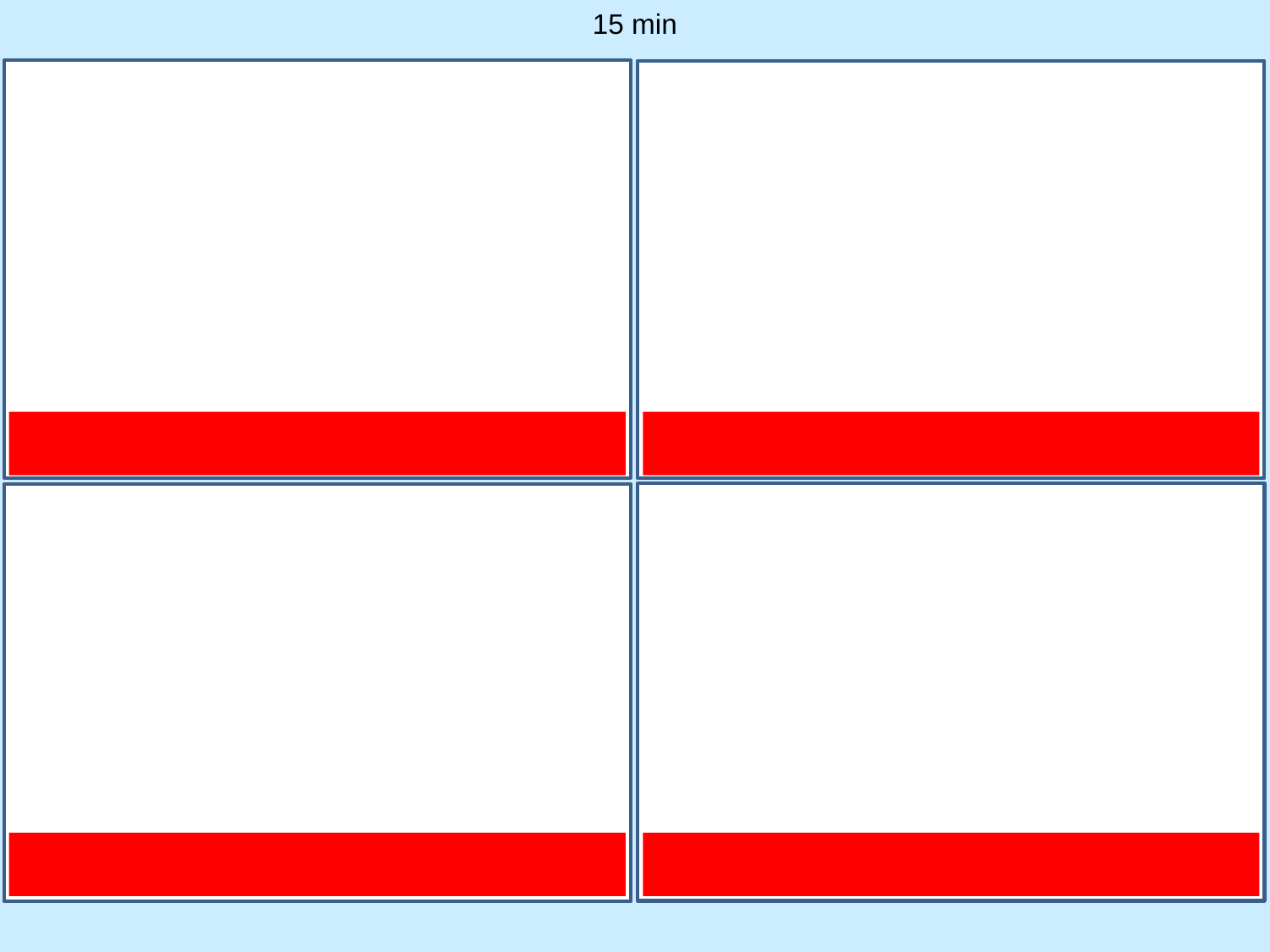

15 min

Supplement: S2 File — (PPTX) [file pone.0183414.s003.pptx]
