# Supplementary material for: The loss and recovery of vertebrate vision examined in microplates
Source: PLoS One. 2017 Aug 17;12(8):e0183414. doi: 10.1371/journal.pone.0183414 (PMC5560659; doi:10.1371/journal.pone.0183414)
Supplement: S3 File — (PPTX) [file pone.0183414.s004.pptx]

## Slide 1
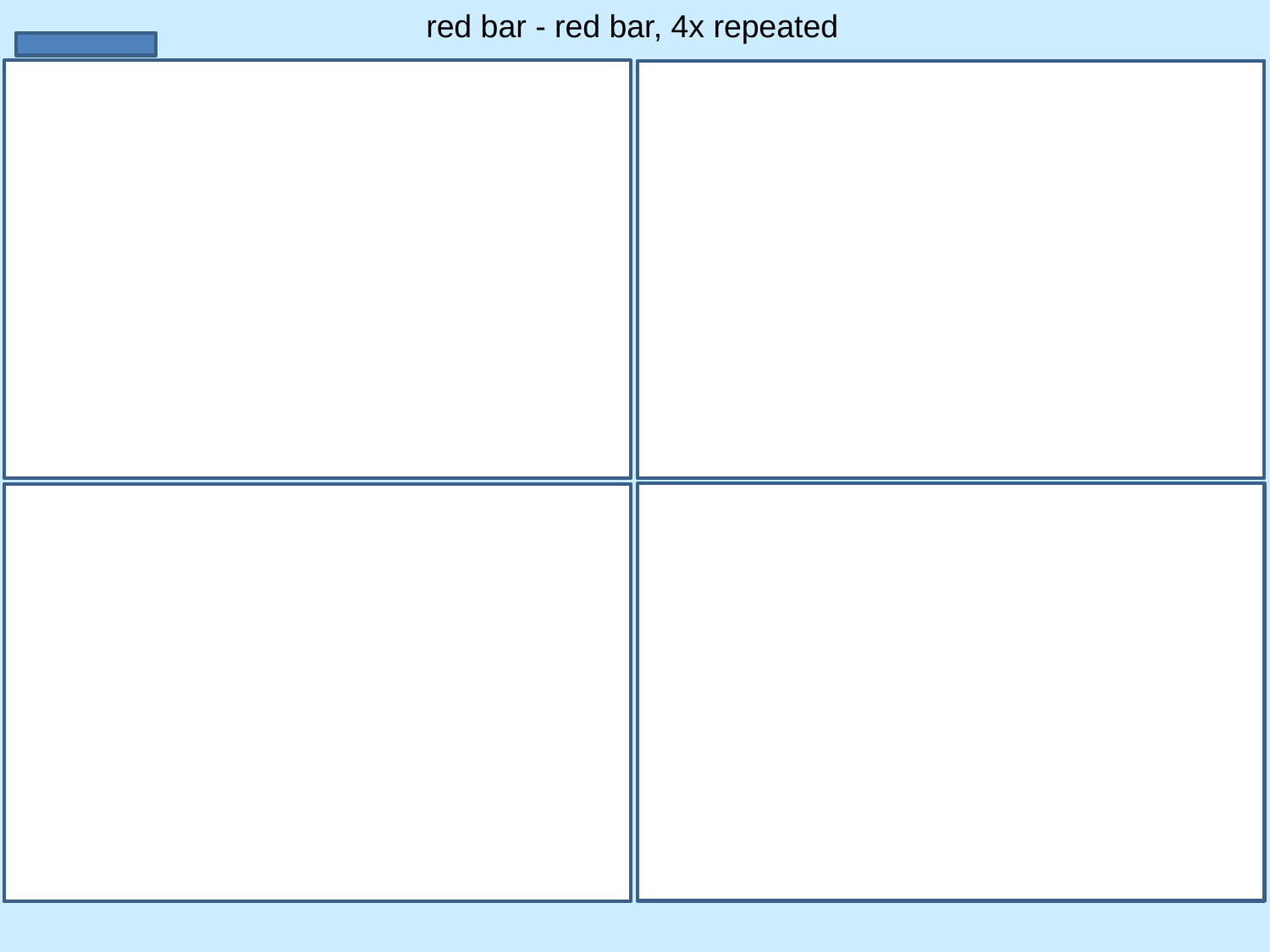

red bar - red bar, 4x repeated

## Slide 2
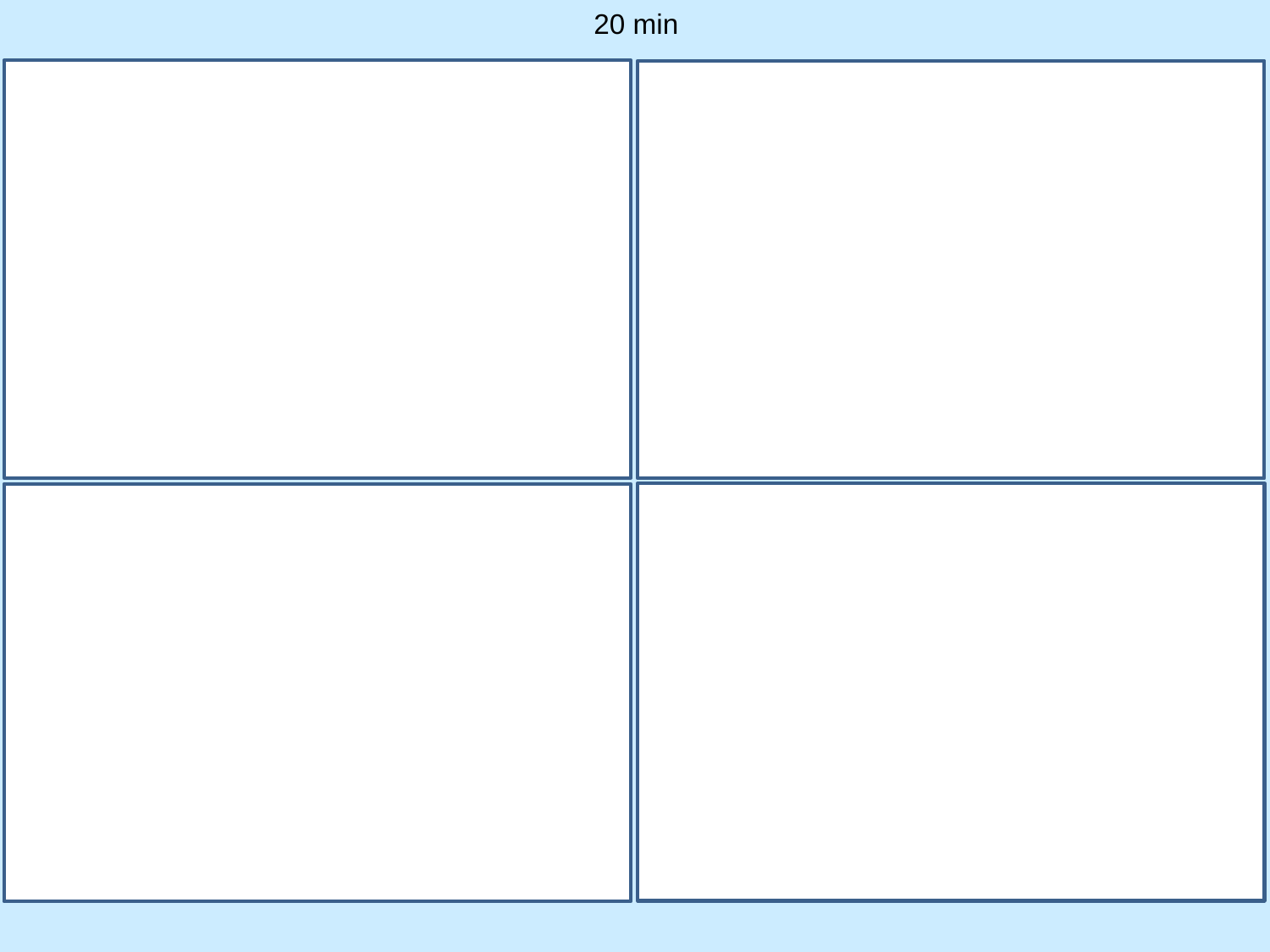

20 min

## Slide 3
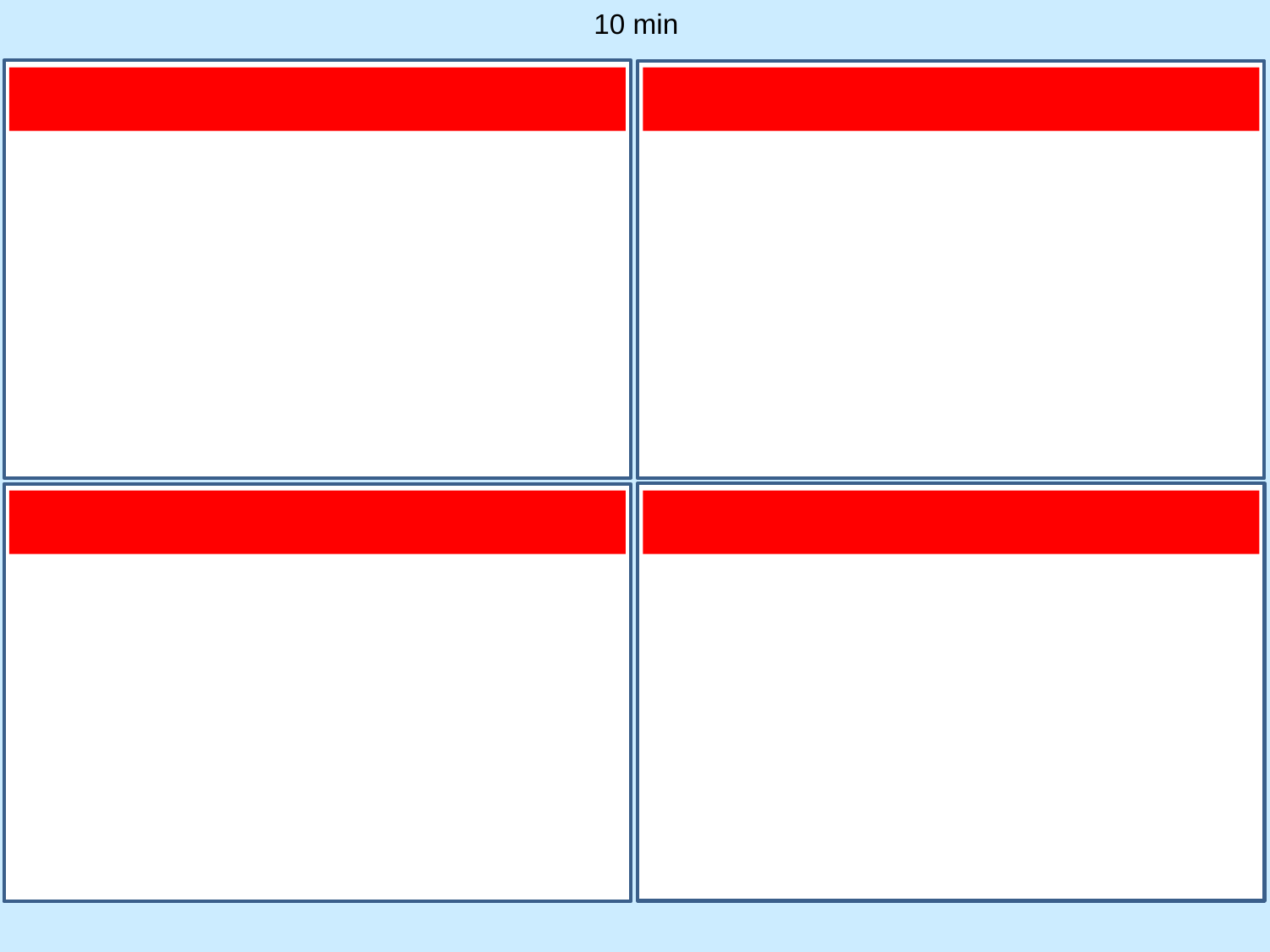

10 min

## Slide 4
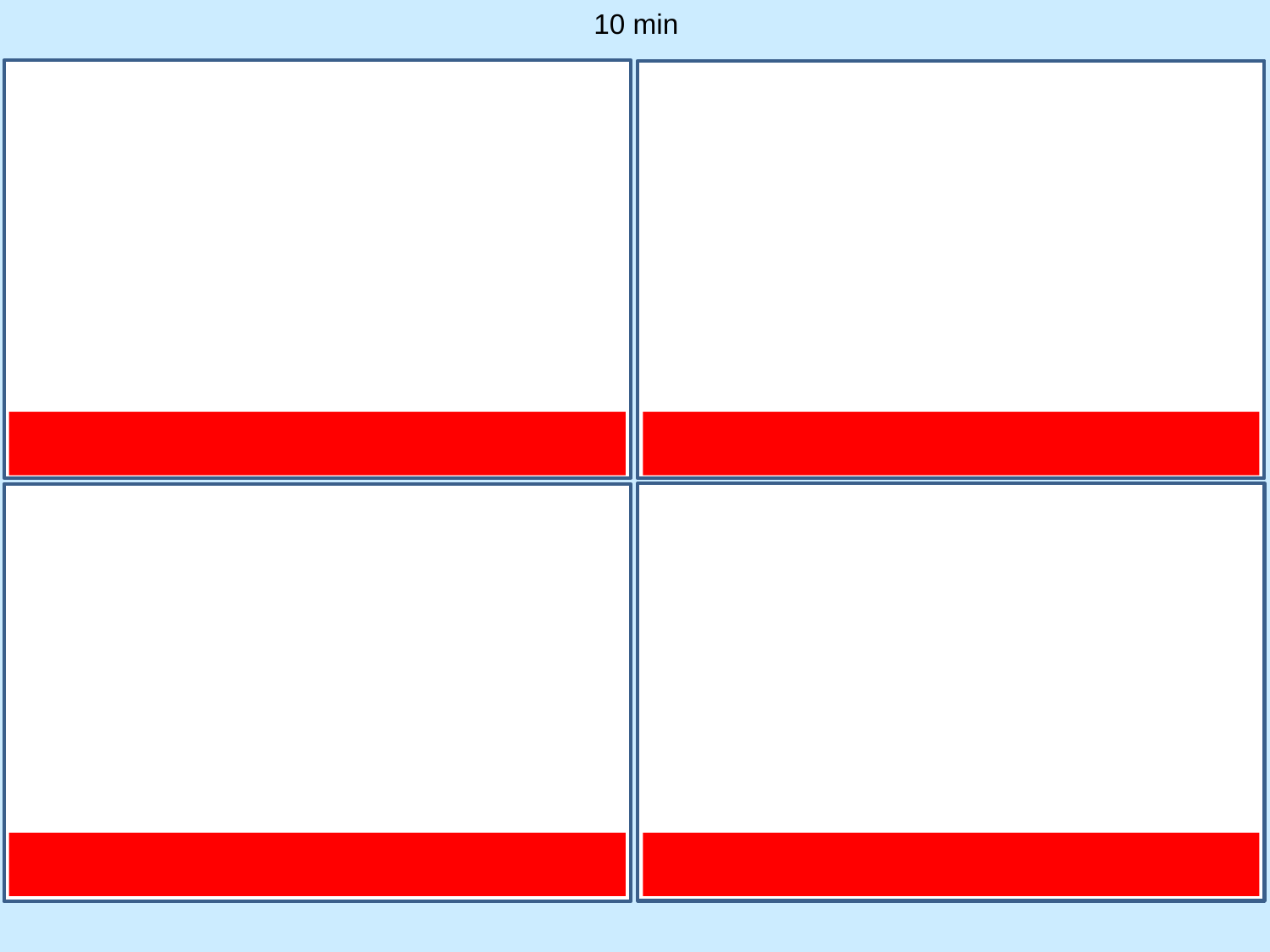

10 min

## Slide 5
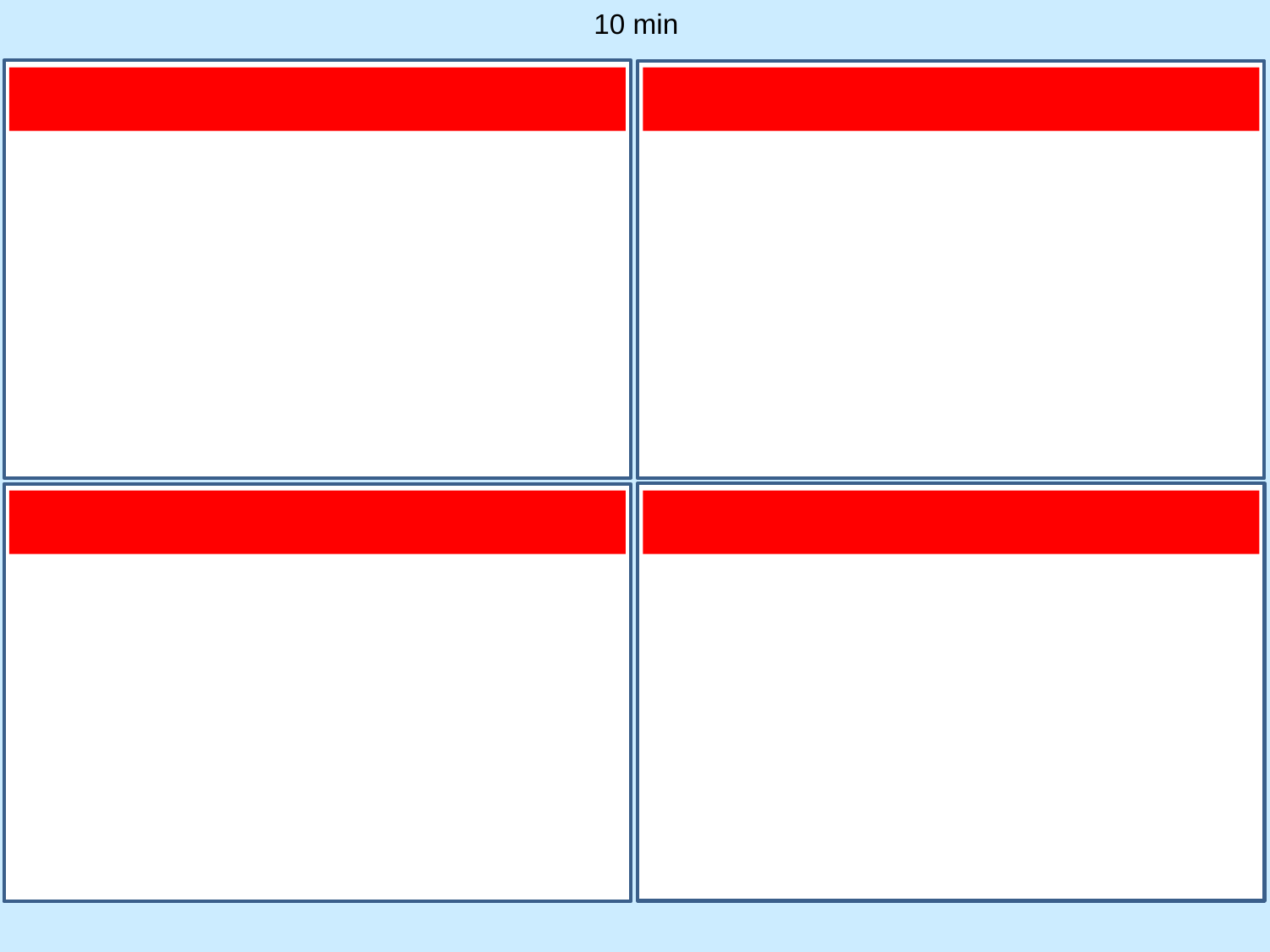

10 min

## Slide 6
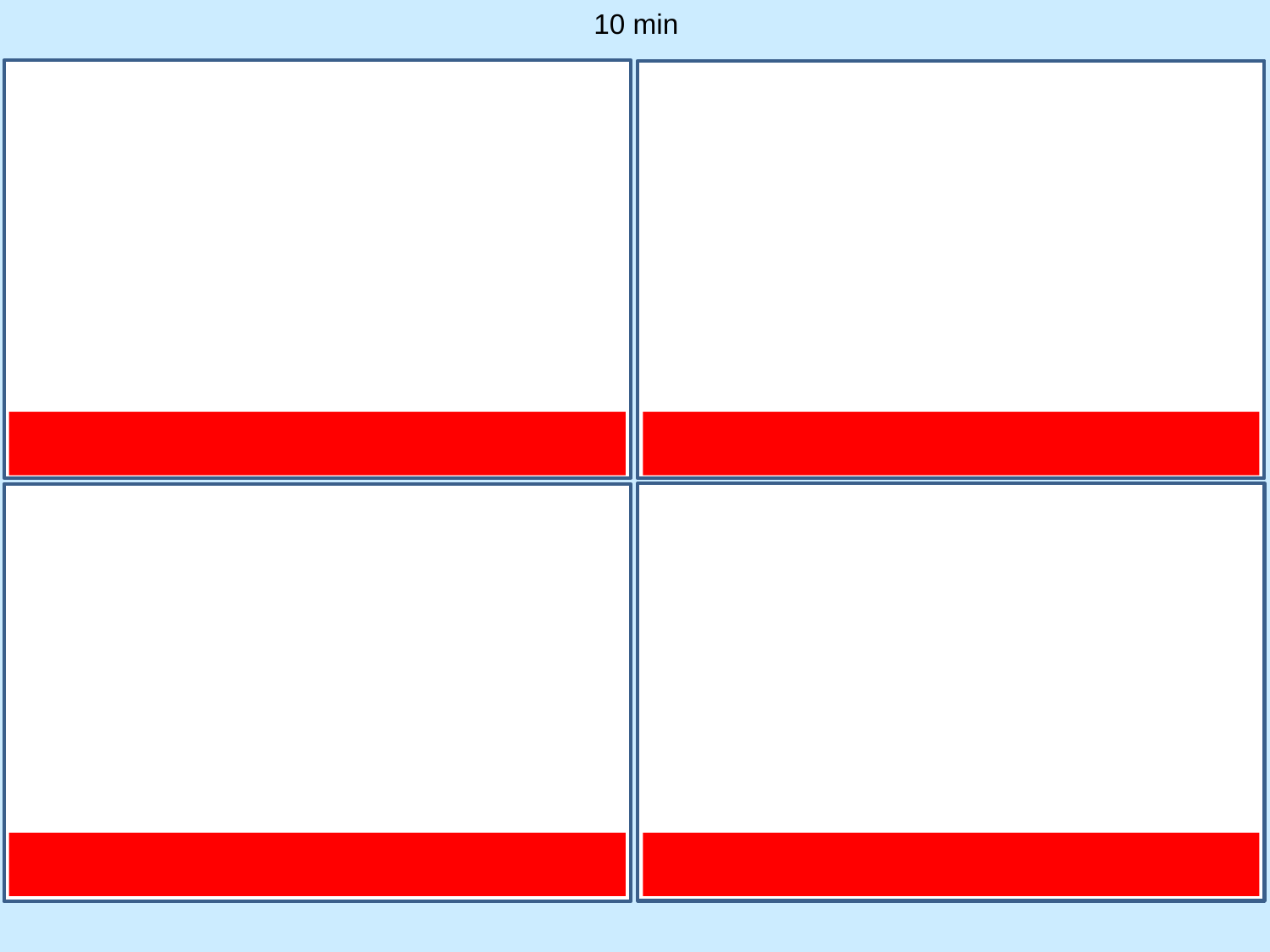

10 min

## Slide 7
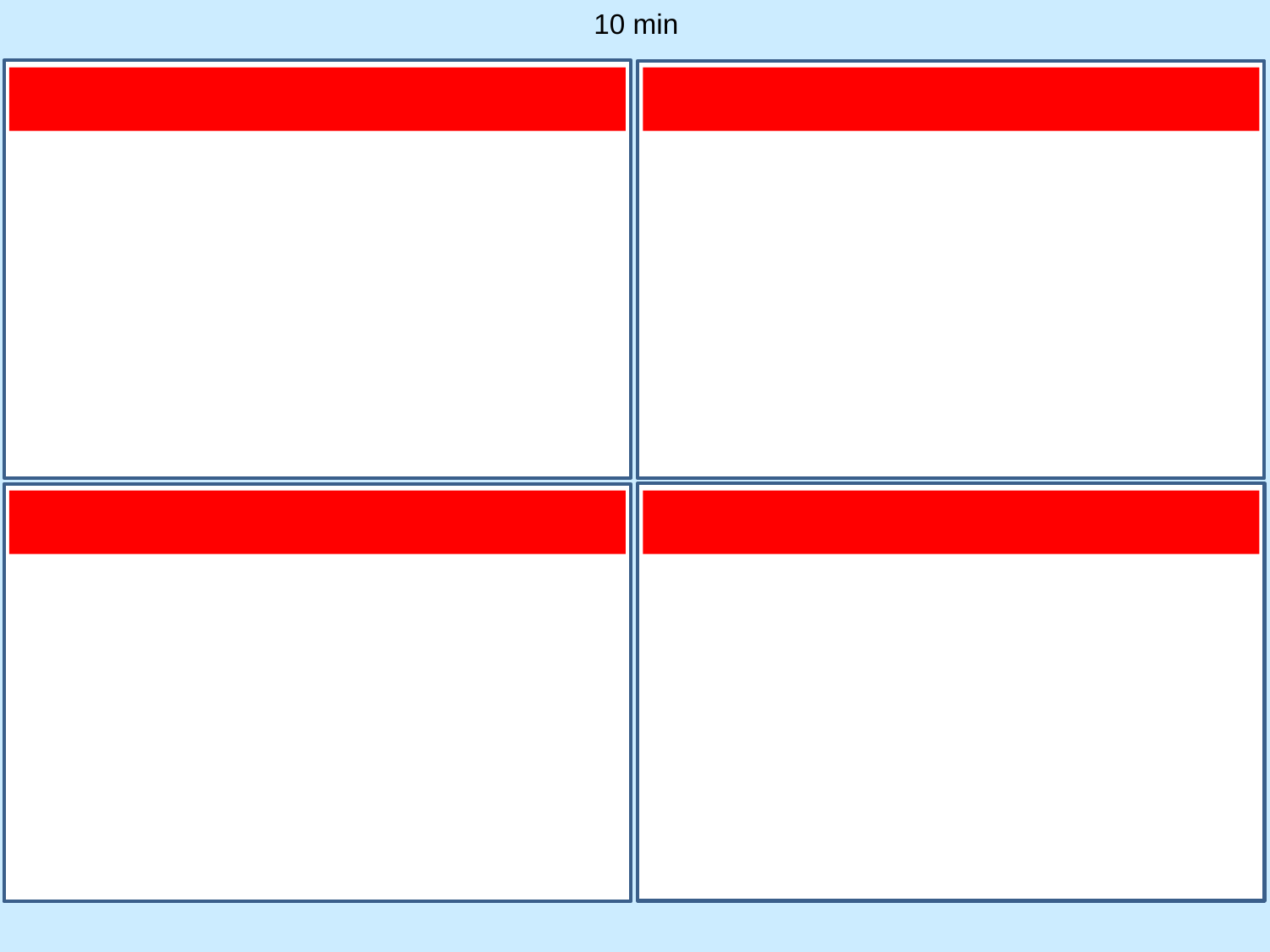

10 min

## Slide 8
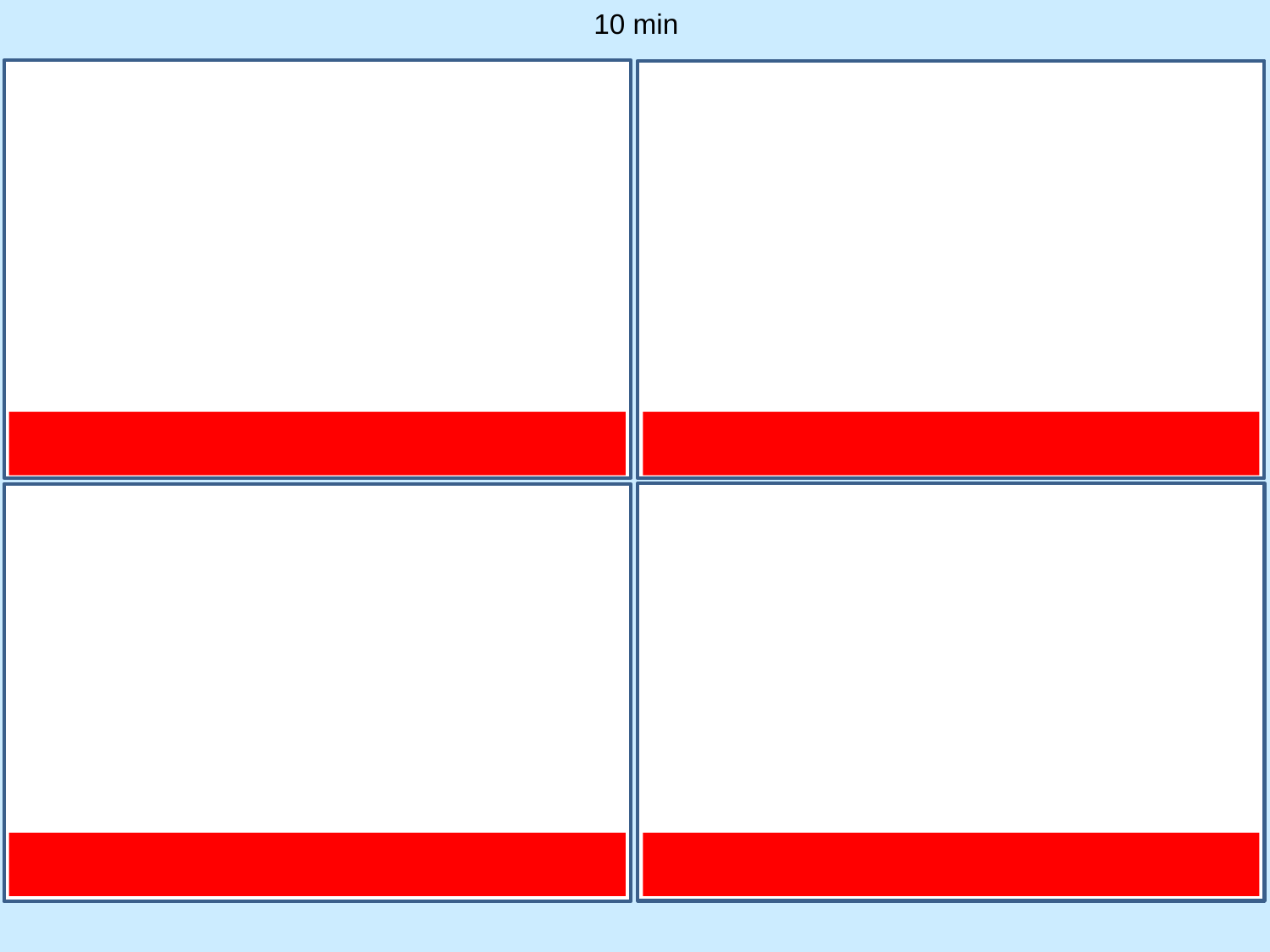

10 min

## Slide 9
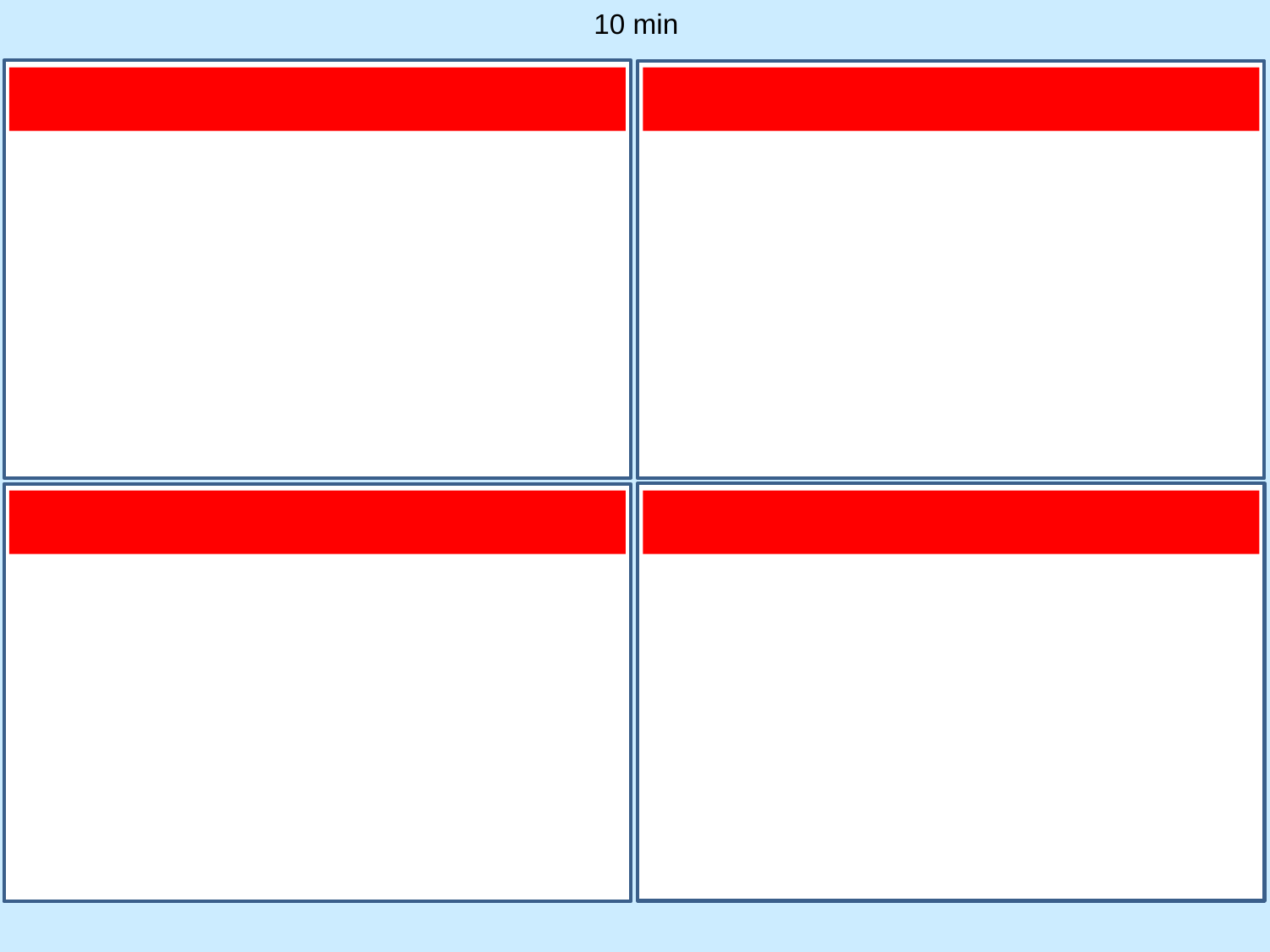

10 min

## Slide 10
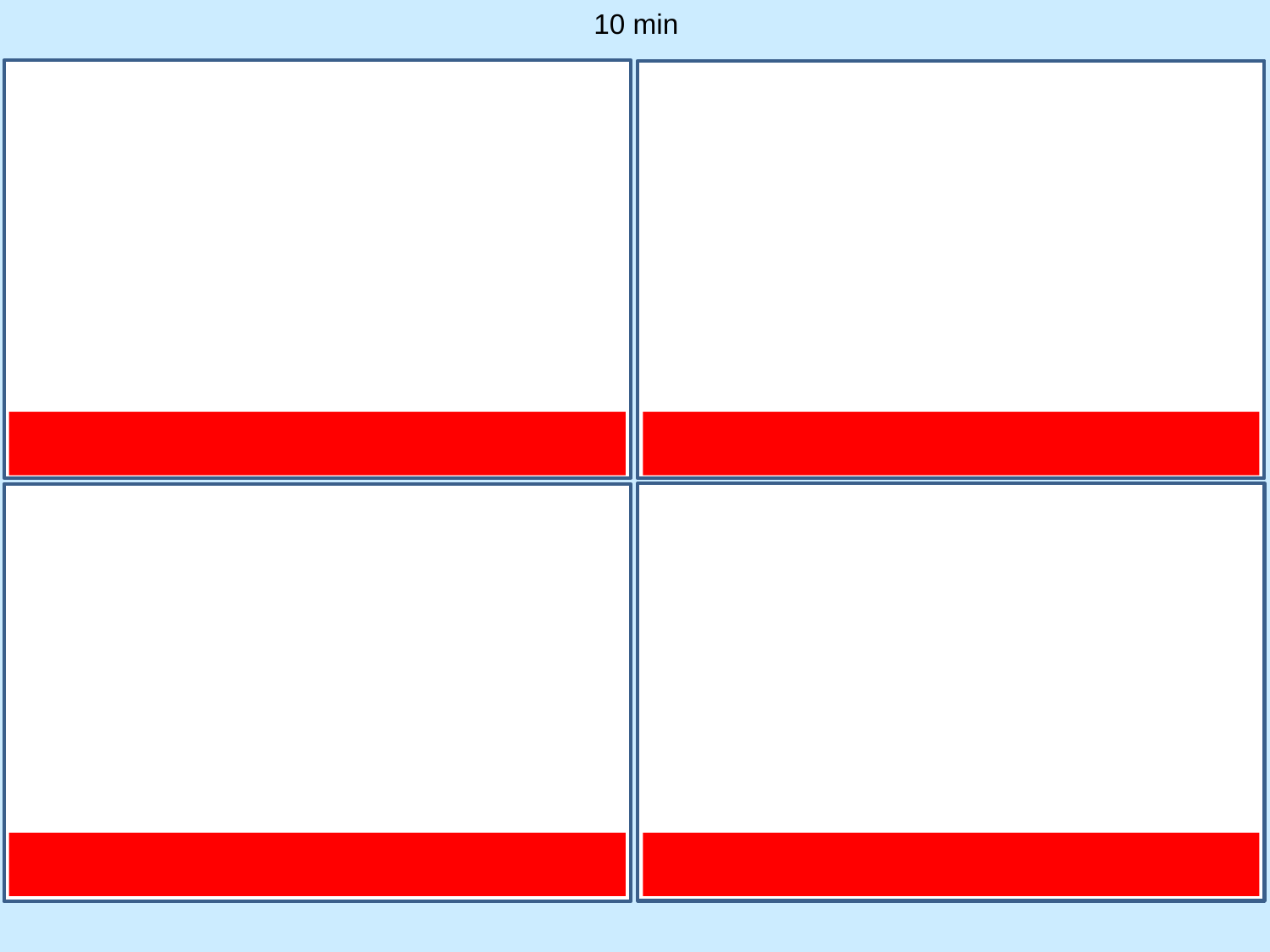

10 min
