# Supplementary material for: The loss and recovery of vertebrate vision examined in microplates
Source: PLoS One. 2017 Aug 17;12(8):e0183414. doi: 10.1371/journal.pone.0183414 (PMC5560659; doi:10.1371/journal.pone.0183414)
Supplement: S4 File — (PPTX) [file pone.0183414.s005.pptx]

## Slide 1
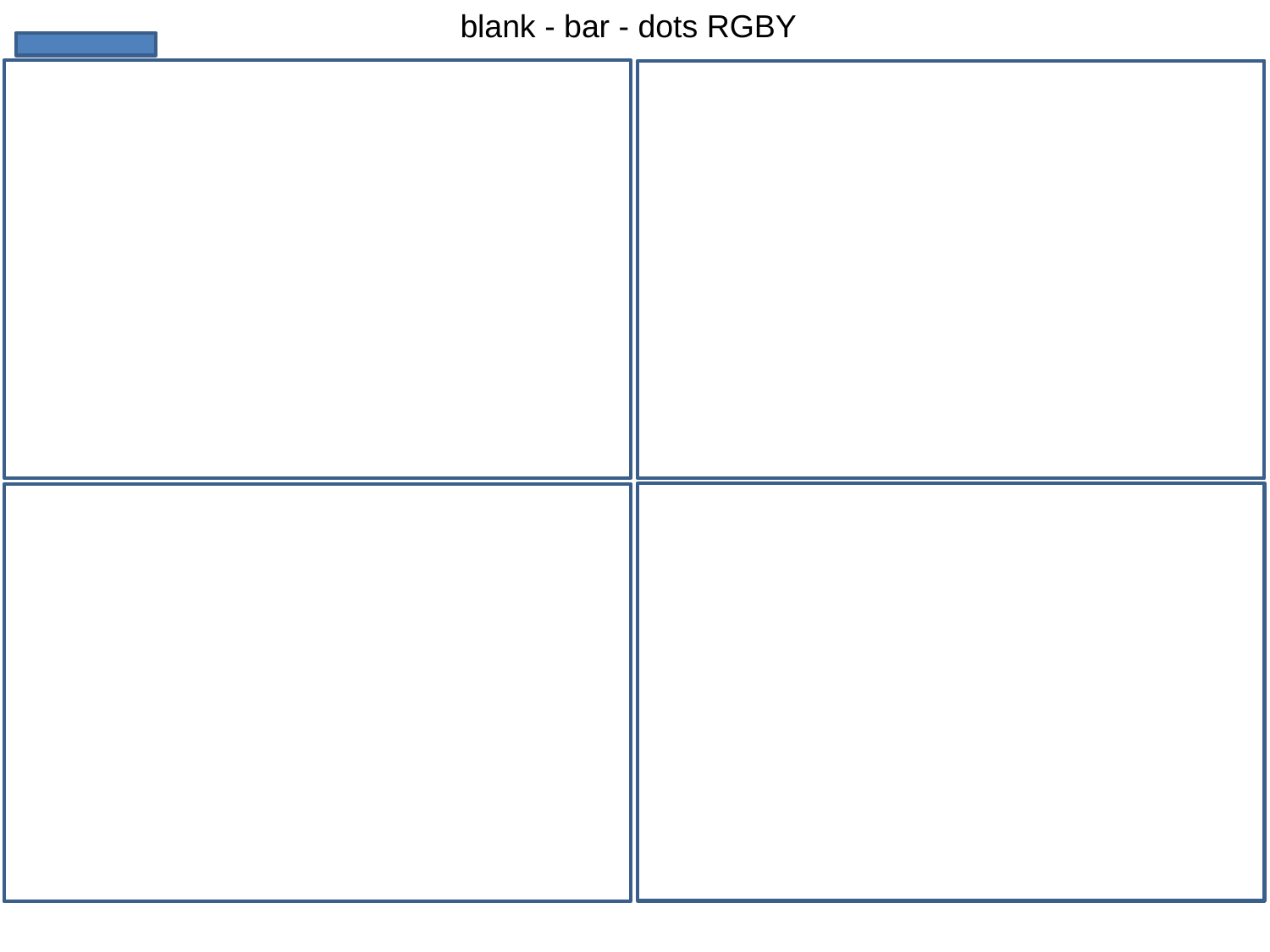

blank - bar - dots RGBY

## Slide 2
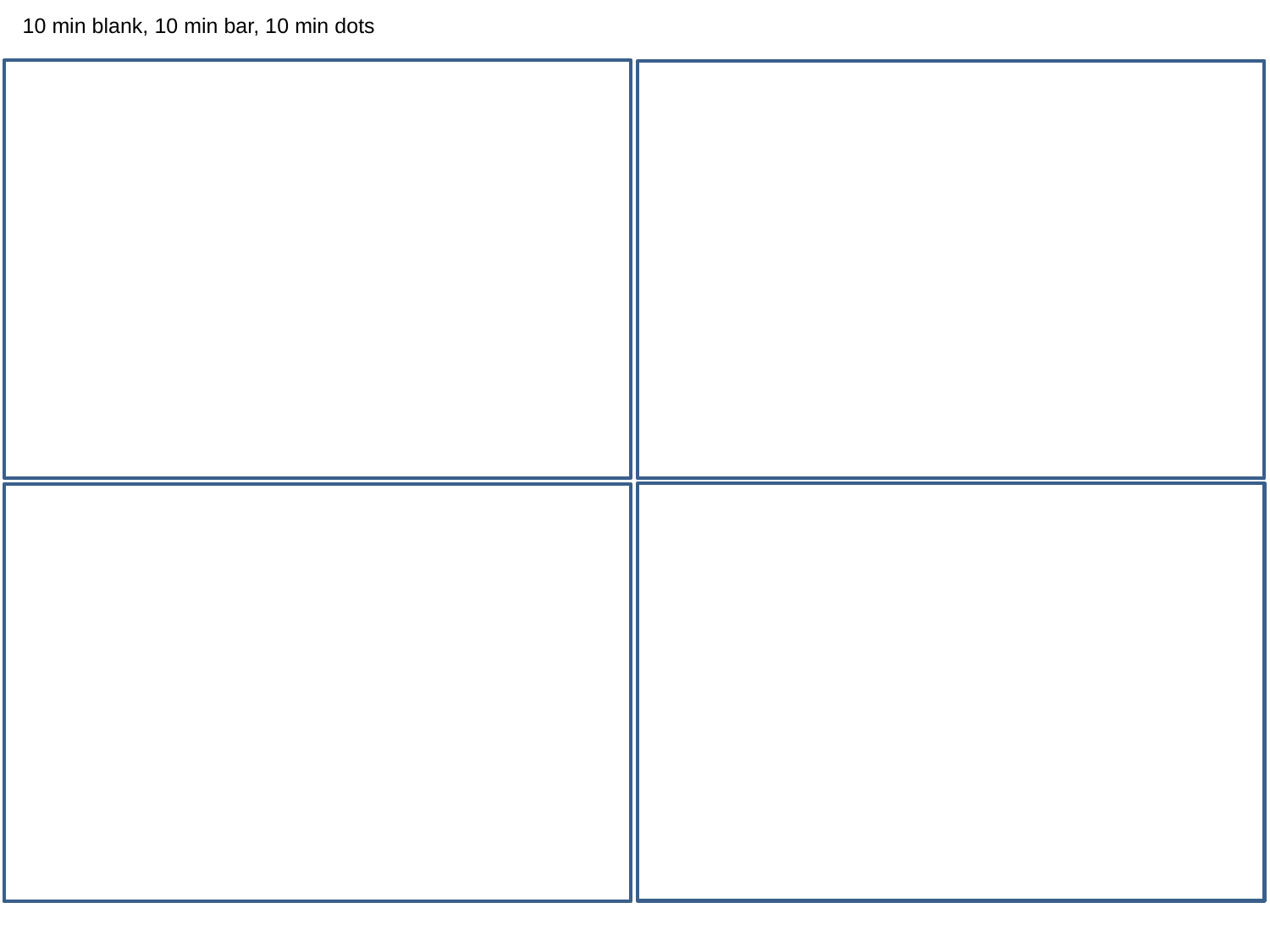

10 min blank, 10 min bar, 10 min dots

## Slide 3
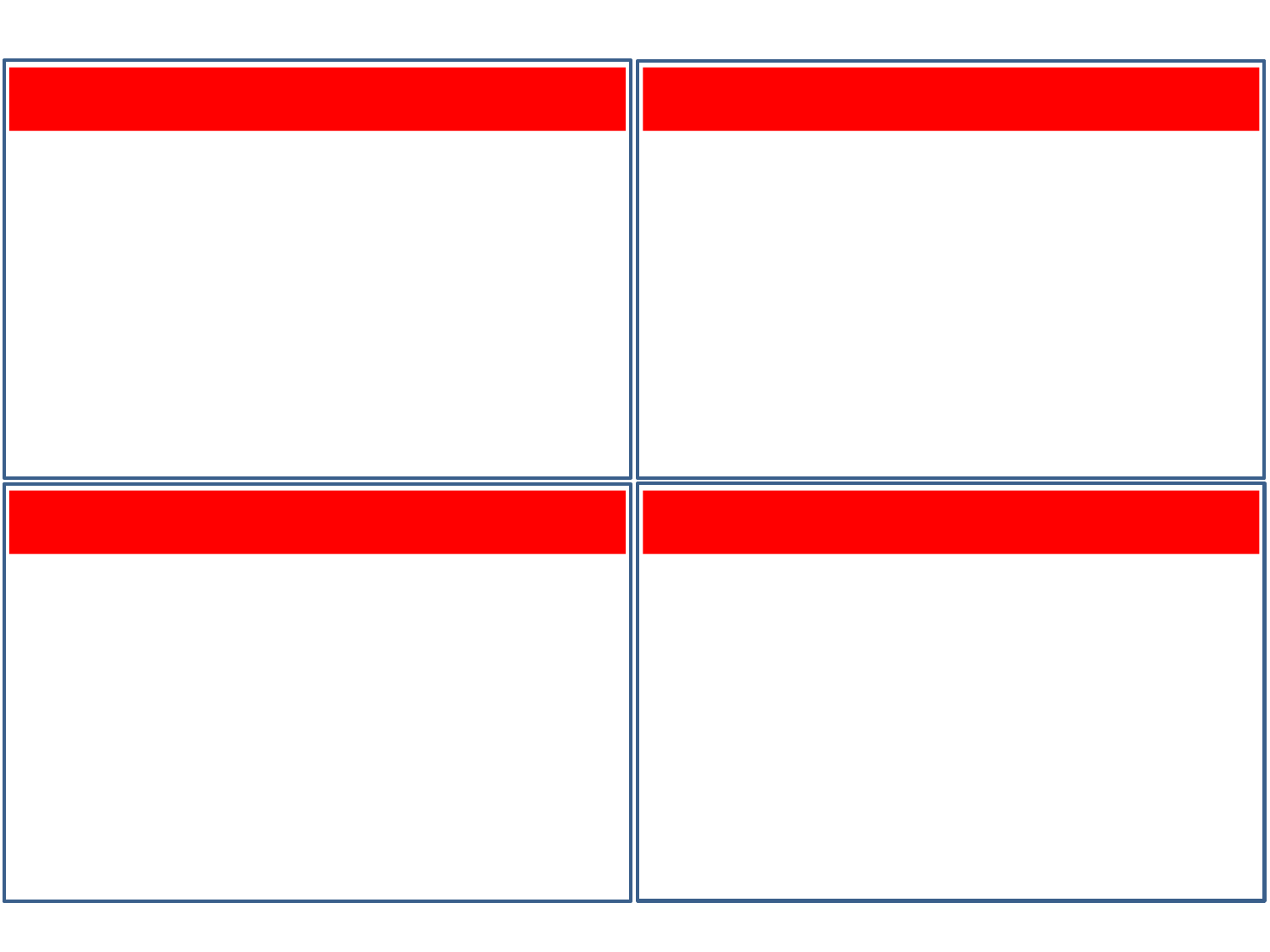

## Slide 4
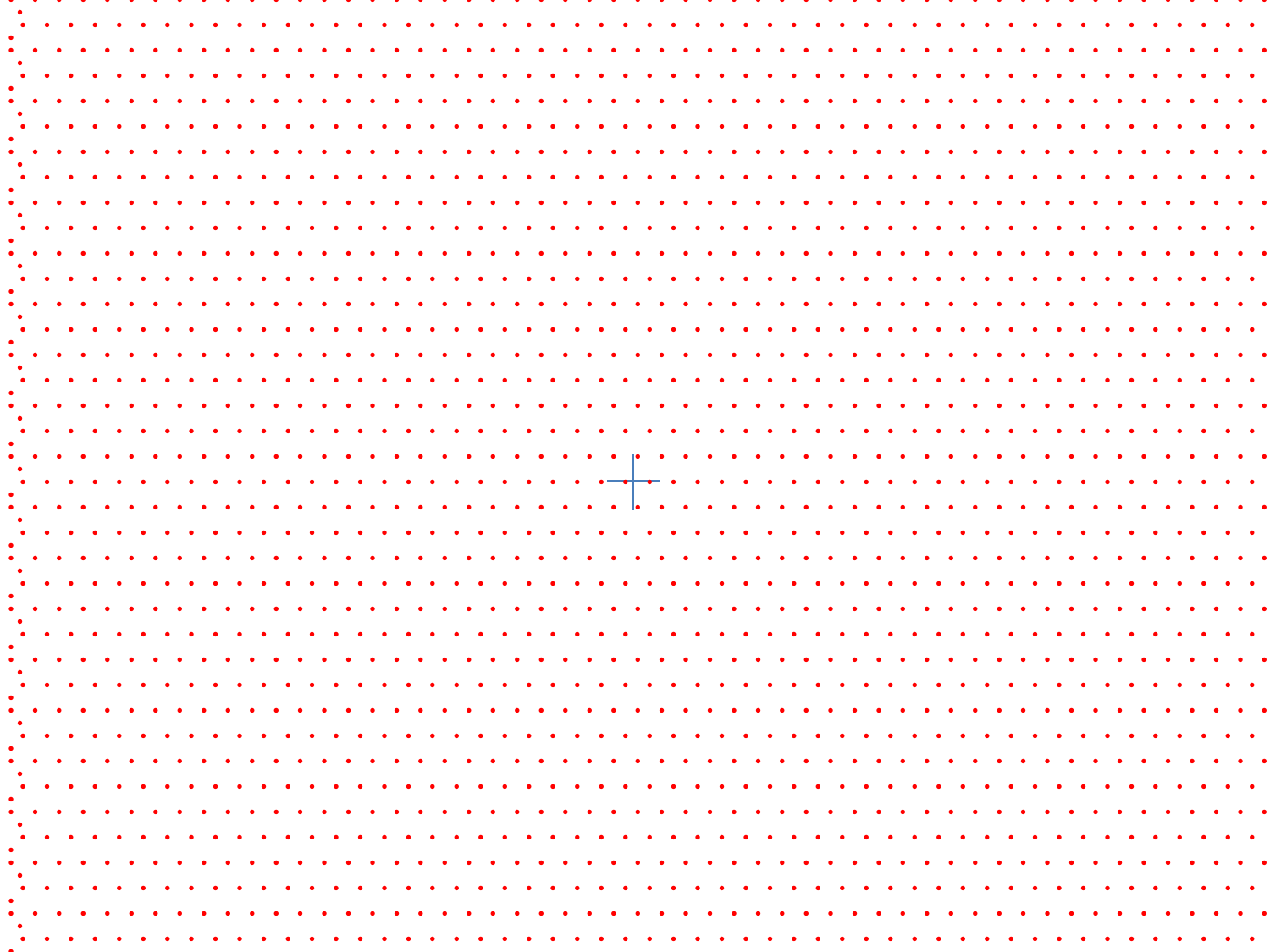

● ● ● ● ● ● ● ● ● ● ● ● ● ● ● ● ● ● ● ● ● ● ● ● ● ● ● ● ● ● ● ● ● ● ● ● ● ● ● ● ● ● ● ● ● ● ● ● ● ● ● ● ● ●
 ● ● ● ● ● ● ● ● ● ● ● ● ● ● ● ● ● ● ● ● ● ● ● ● ● ● ● ● ● ● ● ● ● ● ● ● ● ● ● ● ● ● ● ● ● ● ● ● ● ● ● ● ●
● ● ● ● ● ● ● ● ● ● ● ● ● ● ● ● ● ● ● ● ● ● ● ● ● ● ● ● ● ● ● ● ● ● ● ● ● ● ● ● ● ● ● ● ● ● ● ● ● ● ● ● ● ●
 ● ● ● ● ● ● ● ● ● ● ● ● ● ● ● ● ● ● ● ● ● ● ● ● ● ● ● ● ● ● ● ● ● ● ● ● ● ● ● ● ● ● ● ● ● ● ● ● ● ● ● ● ●
● ● ● ● ● ● ● ● ● ● ● ● ● ● ● ● ● ● ● ● ● ● ● ● ● ● ● ● ● ● ● ● ● ● ● ● ● ● ● ● ● ● ● ● ● ● ● ● ● ● ● ● ● ●
 ● ● ● ● ● ● ● ● ● ● ● ● ● ● ● ● ● ● ● ● ● ● ● ● ● ● ● ● ● ● ● ● ● ● ● ● ● ● ● ● ● ● ● ● ● ● ● ● ● ● ● ● ●
● ● ● ● ● ● ● ● ● ● ● ● ● ● ● ● ● ● ● ● ● ● ● ● ● ● ● ● ● ● ● ● ● ● ● ● ● ● ● ● ● ● ● ● ● ● ● ● ● ● ● ● ● ●
 ● ● ● ● ● ● ● ● ● ● ● ● ● ● ● ● ● ● ● ● ● ● ● ● ● ● ● ● ● ● ● ● ● ● ● ● ● ● ● ● ● ● ● ● ● ● ● ● ● ● ● ● ●
● ● ● ● ● ● ● ● ● ● ● ● ● ● ● ● ● ● ● ● ● ● ● ● ● ● ● ● ● ● ● ● ● ● ● ● ● ● ● ● ● ● ● ● ● ● ● ● ● ● ● ● ● ●
 ● ● ● ● ● ● ● ● ● ● ● ● ● ● ● ● ● ● ● ● ● ● ● ● ● ● ● ● ● ● ● ● ● ● ● ● ● ● ● ● ● ● ● ● ● ● ● ● ● ● ● ● ●
● ● ● ● ● ● ● ● ● ● ● ● ● ● ● ● ● ● ● ● ● ● ● ● ● ● ● ● ● ● ● ● ● ● ● ● ● ● ● ● ● ● ● ● ● ● ● ● ● ● ● ● ● ●
 ● ● ● ● ● ● ● ● ● ● ● ● ● ● ● ● ● ● ● ● ● ● ● ● ● ● ● ● ● ● ● ● ● ● ● ● ● ● ● ● ● ● ● ● ● ● ● ● ● ● ● ● ●
● ● ● ● ● ● ● ● ● ● ● ● ● ● ● ● ● ● ● ● ● ● ● ● ● ● ● ● ● ● ● ● ● ● ● ● ● ● ● ● ● ● ● ● ● ● ● ● ● ● ● ● ● ●
 ● ● ● ● ● ● ● ● ● ● ● ● ● ● ● ● ● ● ● ● ● ● ● ● ● ● ● ● ● ● ● ● ● ● ● ● ● ● ● ● ● ● ● ● ● ● ● ● ● ● ● ● ●
● ● ● ● ● ● ● ● ● ● ● ● ● ● ● ● ● ● ● ● ● ● ● ● ● ● ● ● ● ● ● ● ● ● ● ● ● ● ● ● ● ● ● ● ● ● ● ● ● ● ● ● ● ●
 ● ● ● ● ● ● ● ● ● ● ● ● ● ● ● ● ● ● ● ● ● ● ● ● ● ● ● ● ● ● ● ● ● ● ● ● ● ● ● ● ● ● ● ● ● ● ● ● ● ● ● ● ●
● ● ● ● ● ● ● ● ● ● ● ● ● ● ● ● ● ● ● ● ● ● ● ● ● ● ● ● ● ● ● ● ● ● ● ● ● ● ● ● ● ● ● ● ● ● ● ● ● ● ● ● ● ●
 ● ● ● ● ● ● ● ● ● ● ● ● ● ● ● ● ● ● ● ● ● ● ● ● ● ● ● ● ● ● ● ● ● ● ● ● ● ● ● ● ● ● ● ● ● ● ● ● ● ● ● ● ●
● ● ● ● ● ● ● ● ● ● ● ● ● ● ● ● ● ● ● ● ● ● ● ● ● ● ● ● ● ● ● ● ● ● ● ● ● ● ● ● ● ● ● ● ● ● ● ● ● ● ● ● ● ●
 ● ● ● ● ● ● ● ● ● ● ● ● ● ● ● ● ● ● ● ● ● ● ● ● ● ● ● ● ● ● ● ● ● ● ● ● ● ● ● ● ● ● ● ● ● ● ● ● ● ● ● ● ●
● ● ● ● ● ● ● ● ● ● ● ● ● ● ● ● ● ● ● ● ● ● ● ● ● ● ● ● ● ● ● ● ● ● ● ● ● ● ● ● ● ● ● ● ● ● ● ● ● ● ● ● ● ●
 ● ● ● ● ● ● ● ● ● ● ● ● ● ● ● ● ● ● ● ● ● ● ● ● ● ● ● ● ● ● ● ● ● ● ● ● ● ● ● ● ● ● ● ● ● ● ● ● ● ● ● ● ●
● ● ● ● ● ● ● ● ● ● ● ● ● ● ● ● ● ● ● ● ● ● ● ● ● ● ● ● ● ● ● ● ● ● ● ● ● ● ● ● ● ● ● ● ● ● ● ● ● ● ● ● ● ●
 ● ● ● ● ● ● ● ● ● ● ● ● ● ● ● ● ● ● ● ● ● ● ● ● ● ● ● ● ● ● ● ● ● ● ● ● ● ● ● ● ● ● ● ● ● ● ● ● ● ● ● ● ●
● ● ● ● ● ● ● ● ● ● ● ● ● ● ● ● ● ● ● ● ● ● ● ● ● ● ● ● ● ● ● ● ● ● ● ● ● ● ● ● ● ● ● ● ● ● ● ● ● ● ● ● ● ●
 ● ● ● ● ● ● ● ● ● ● ● ● ● ● ● ● ● ● ● ● ● ● ● ● ● ● ● ● ● ● ● ● ● ● ● ● ● ● ● ● ● ● ● ● ● ● ● ● ● ● ● ● ●
● ● ● ● ● ● ● ● ● ● ● ● ● ● ● ● ● ● ● ● ● ● ● ● ● ● ● ● ● ● ● ● ● ● ● ● ● ● ● ● ● ● ● ● ● ● ● ● ● ● ● ● ● ●
 ● ● ● ● ● ● ● ● ● ● ● ● ● ● ● ● ● ● ● ● ● ● ● ● ● ● ● ● ● ● ● ● ● ● ● ● ● ● ● ● ● ● ● ● ● ● ● ● ● ● ● ● ●
● ● ● ● ● ● ● ● ● ● ● ● ● ● ● ● ● ● ● ● ● ● ● ● ● ● ● ● ● ● ● ● ● ● ● ● ● ● ● ● ● ● ● ● ● ● ● ● ● ● ● ● ● ●
 ● ● ● ● ● ● ● ● ● ● ● ● ● ● ● ● ● ● ● ● ● ● ● ● ● ● ● ● ● ● ● ● ● ● ● ● ● ● ● ● ● ● ● ● ● ● ● ● ● ● ● ● ●
● ● ● ● ● ● ● ● ● ● ● ● ● ● ● ● ● ● ● ● ● ● ● ● ● ● ● ● ● ● ● ● ● ● ● ● ● ● ● ● ● ● ● ● ● ● ● ● ● ● ● ● ● ●
 ● ● ● ● ● ● ● ● ● ● ● ● ● ● ● ● ● ● ● ● ● ● ● ● ● ● ● ● ● ● ● ● ● ● ● ● ● ● ● ● ● ● ● ● ● ● ● ● ● ● ● ● ●
● ● ● ● ● ● ● ● ● ● ● ● ● ● ● ● ● ● ● ● ● ● ● ● ● ● ● ● ● ● ● ● ● ● ● ● ● ● ● ● ● ● ● ● ● ● ● ● ● ● ● ● ● ●
 ● ● ● ● ● ● ● ● ● ● ● ● ● ● ● ● ● ● ● ● ● ● ● ● ● ● ● ● ● ● ● ● ● ● ● ● ● ● ● ● ● ● ● ● ● ● ● ● ● ● ● ● ●
● ● ● ● ● ● ● ● ● ● ● ● ● ● ● ● ● ● ● ● ● ● ● ● ● ● ● ● ● ● ● ● ● ● ● ● ● ● ● ● ● ● ● ● ● ● ● ● ● ● ● ● ● ●
 ● ● ● ● ● ● ● ● ● ● ● ● ● ● ● ● ● ● ● ● ● ● ● ● ● ● ● ● ● ● ● ● ● ● ● ● ● ● ● ● ● ● ● ● ● ● ● ● ● ● ● ● ●
● ● ● ● ● ● ● ● ● ● ● ● ● ● ● ● ● ● ● ● ● ● ● ● ● ● ● ● ● ● ● ● ● ● ● ● ● ● ● ● ● ● ● ● ● ● ● ● ● ● ● ● ● ●
 ● ● ● ● ● ● ● ● ● ● ● ● ● ● ● ● ● ● ● ● ● ● ● ● ● ● ● ● ● ● ● ● ● ● ● ● ● ● ● ● ● ● ● ● ● ● ● ● ● ● ● ● ●
● ● ● ● ● ● ● ● ● ● ● ● ● ● ● ● ● ● ● ● ● ● ● ● ● ● ● ● ● ● ● ● ● ● ● ● ● ● ● ● ● ● ● ● ● ● ● ● ● ● ● ● ● ●
 ● ● ● ● ● ● ● ● ● ● ● ● ● ● ● ● ● ● ● ● ● ● ● ● ● ● ● ● ● ● ● ● ● ● ● ● ● ● ● ● ● ● ● ● ● ● ● ● ● ● ● ● ●
● ● ● ● ● ● ● ● ● ● ● ● ● ● ● ● ● ● ● ● ● ● ● ● ● ● ● ● ● ● ● ● ● ● ● ● ● ● ● ● ● ● ● ● ● ● ● ● ● ● ● ● ● ●
 ● ● ● ● ● ● ● ● ● ● ● ● ● ● ● ● ● ● ● ● ● ● ● ● ● ● ● ● ● ● ● ● ● ● ● ● ● ● ● ● ● ● ● ● ● ● ● ● ● ● ● ● ●
● ● ● ● ● ● ● ● ● ● ● ● ● ● ● ● ● ● ● ● ● ● ● ● ● ● ● ● ● ● ● ● ● ● ● ● ● ● ● ● ● ● ● ● ● ● ● ● ● ● ● ● ● ●
 ● ● ● ● ● ● ● ● ● ● ● ● ● ● ● ● ● ● ● ● ● ● ● ● ● ● ● ● ● ● ● ● ● ● ● ● ● ● ● ● ● ● ● ● ● ● ● ● ● ● ● ● ●
● ● ● ● ● ● ● ● ● ● ● ● ● ● ● ● ● ● ● ● ● ● ● ● ● ● ● ● ● ● ● ● ● ● ● ● ● ● ● ● ● ● ● ● ● ● ● ● ● ● ● ● ● ●
 ● ● ● ● ● ● ● ● ● ● ● ● ● ● ● ● ● ● ● ● ● ● ● ● ● ● ● ● ● ● ● ● ● ● ● ● ● ● ● ● ● ● ● ● ● ● ● ● ● ● ● ● ●
● ● ● ● ● ● ● ● ● ● ● ● ● ● ● ● ● ● ● ● ● ● ● ● ● ● ● ● ● ● ● ● ● ● ● ● ● ● ● ● ● ● ● ● ● ● ● ● ● ● ● ● ● ●
 ● ● ● ● ● ● ● ● ● ● ● ● ● ● ● ● ● ● ● ● ● ● ● ● ● ● ● ● ● ● ● ● ● ● ● ● ● ● ● ● ● ● ● ● ● ● ● ● ● ● ● ● ●
● ● ● ● ● ● ● ● ● ● ● ● ● ● ● ● ● ● ● ● ● ● ● ● ● ● ● ● ● ● ● ● ● ● ● ● ● ● ● ● ● ● ● ● ● ● ● ● ● ● ● ● ● ●
 ● ● ● ● ● ● ● ● ● ● ● ● ● ● ● ● ● ● ● ● ● ● ● ● ● ● ● ● ● ● ● ● ● ● ● ● ● ● ● ● ● ● ● ● ● ● ● ● ● ● ● ● ●
● ● ● ● ● ● ● ● ● ● ● ● ● ● ● ● ● ● ● ● ● ● ● ● ● ● ● ● ● ● ● ● ● ● ● ● ● ● ● ● ● ● ● ● ● ● ● ● ● ● ● ● ● ●
 ● ● ● ● ● ● ● ● ● ● ● ● ● ● ● ● ● ● ● ● ● ● ● ● ● ● ● ● ● ● ● ● ● ● ● ● ● ● ● ● ● ● ● ● ● ● ● ● ● ● ● ● ●
● ● ● ● ● ● ● ● ● ● ● ● ● ● ● ● ● ● ● ● ● ● ● ● ● ● ● ● ● ● ● ● ● ● ● ● ● ● ● ● ● ● ● ● ● ● ● ● ● ● ● ● ● ●
 ● ● ● ● ● ● ● ● ● ● ● ● ● ● ● ● ● ● ● ● ● ● ● ● ● ● ● ● ● ● ● ● ● ● ● ● ● ● ● ● ● ● ● ● ● ● ● ● ● ● ● ● ●
● ● ● ● ● ● ● ● ● ● ● ● ● ● ● ● ● ● ● ● ● ● ● ● ● ● ● ● ● ● ● ● ● ● ● ● ● ● ● ● ● ● ● ● ● ● ● ● ● ● ● ● ● ●
 ● ● ● ● ● ● ● ● ● ● ● ● ● ● ● ● ● ● ● ● ● ● ● ● ● ● ● ● ● ● ● ● ● ● ● ● ● ● ● ● ● ● ● ● ● ● ● ● ● ● ● ● ●
● ● ● ● ● ● ● ● ● ● ● ● ● ● ● ● ● ● ● ● ● ● ● ● ● ● ● ● ● ● ● ● ● ● ● ● ● ● ● ● ● ● ● ● ● ● ● ● ● ● ● ● ● ●
 ● ● ● ● ● ● ● ● ● ● ● ● ● ● ● ● ● ● ● ● ● ● ● ● ● ● ● ● ● ● ● ● ● ● ● ● ● ● ● ● ● ● ● ● ● ● ● ● ● ● ● ● ●
● ● ● ● ● ● ● ● ● ● ● ● ● ● ● ● ● ● ● ● ● ● ● ● ● ● ● ● ● ● ● ● ● ● ● ● ● ● ● ● ● ● ● ● ● ● ● ● ● ● ● ● ● ●
 ● ● ● ● ● ● ● ● ● ● ● ● ● ● ● ● ● ● ● ● ● ● ● ● ● ● ● ● ● ● ● ● ● ● ● ● ● ● ● ● ● ● ● ● ● ● ● ● ● ● ● ● ●
● ● ● ● ● ● ● ● ● ● ● ● ● ● ● ● ● ● ● ● ● ● ● ● ● ● ● ● ● ● ● ● ● ● ● ● ● ● ● ● ● ● ● ● ● ● ● ● ● ● ● ● ● ●
 ● ● ● ● ● ● ● ● ● ● ● ● ● ● ● ● ● ● ● ● ● ● ● ● ● ● ● ● ● ● ● ● ● ● ● ● ● ● ● ● ● ● ● ● ● ● ● ● ● ● ● ● ●
● ● ● ● ● ● ● ● ● ● ● ● ● ● ● ● ● ● ● ● ● ● ● ● ● ● ● ● ● ● ● ● ● ● ● ● ● ● ● ● ● ● ● ● ● ● ● ● ● ● ● ● ● ●
 ● ● ● ● ● ● ● ● ● ● ● ● ● ● ● ● ● ● ● ● ● ● ● ● ● ● ● ● ● ● ● ● ● ● ● ● ● ● ● ● ● ● ● ● ● ● ● ● ● ● ● ● ●
● ● ● ● ● ● ● ● ● ● ● ● ● ● ● ● ● ● ● ● ● ● ● ● ● ● ● ● ● ● ● ● ● ● ● ● ● ● ● ● ● ● ● ● ● ● ● ● ● ● ● ● ● ●
 ● ● ● ● ● ● ● ● ● ● ● ● ● ● ● ● ● ● ● ● ● ● ● ● ● ● ● ● ● ● ● ● ● ● ● ● ● ● ● ● ● ● ● ● ● ● ● ● ● ● ● ● ●
● ● ● ● ● ● ● ● ● ● ● ● ● ● ● ● ● ● ● ● ● ● ● ● ● ● ● ● ● ● ● ● ● ● ● ● ● ● ● ● ● ● ● ● ● ● ● ● ● ● ● ● ● ●
 ● ● ● ● ● ● ● ● ● ● ● ● ● ● ● ● ● ● ● ● ● ● ● ● ● ● ● ● ● ● ● ● ● ● ● ● ● ● ● ● ● ● ● ● ● ● ● ● ● ● ● ● ●
● ● ● ● ● ● ● ● ● ● ● ● ● ● ● ● ● ● ● ● ● ● ● ● ● ● ● ● ● ● ● ● ● ● ● ● ● ● ● ● ● ● ● ● ● ● ● ● ● ● ● ● ● ●
 ● ● ● ● ● ● ● ● ● ● ● ● ● ● ● ● ● ● ● ● ● ● ● ● ● ● ● ● ● ● ● ● ● ● ● ● ● ● ● ● ● ● ● ● ● ● ● ● ● ● ● ● ●
● ● ● ● ● ● ● ● ● ● ● ● ● ● ● ● ● ● ● ● ● ● ● ● ● ● ● ● ● ● ● ● ● ● ● ● ● ● ● ● ● ● ● ● ● ● ● ● ● ● ● ● ● ●
 ● ● ● ● ● ● ● ● ● ● ● ● ● ● ● ● ● ● ● ● ● ● ● ● ● ● ● ● ● ● ● ● ● ● ● ● ● ● ● ● ● ● ● ● ● ● ● ● ● ● ● ● ●
● ● ● ● ● ● ● ● ● ● ● ● ● ● ● ● ● ● ● ● ● ● ● ● ● ● ● ● ● ● ● ● ● ● ● ● ● ● ● ● ● ● ● ● ● ● ● ● ● ● ● ● ● ●
 ● ● ● ● ● ● ● ● ● ● ● ● ● ● ● ● ● ● ● ● ● ● ● ● ● ● ● ● ● ● ● ● ● ● ● ● ● ● ● ● ● ● ● ● ● ● ● ● ● ● ● ● ●
● ● ● ● ● ● ● ● ● ● ● ● ● ● ● ● ● ● ● ● ● ● ● ● ● ● ● ● ● ● ● ● ● ● ● ● ● ● ● ● ● ● ● ● ● ● ● ● ● ● ● ● ● ●
 ● ● ● ● ● ● ● ● ● ● ● ● ● ● ● ● ● ● ● ● ● ● ● ● ● ● ● ● ● ● ● ● ● ● ● ● ● ● ● ● ● ● ● ● ● ● ● ● ● ● ● ● ●

## Slide 5
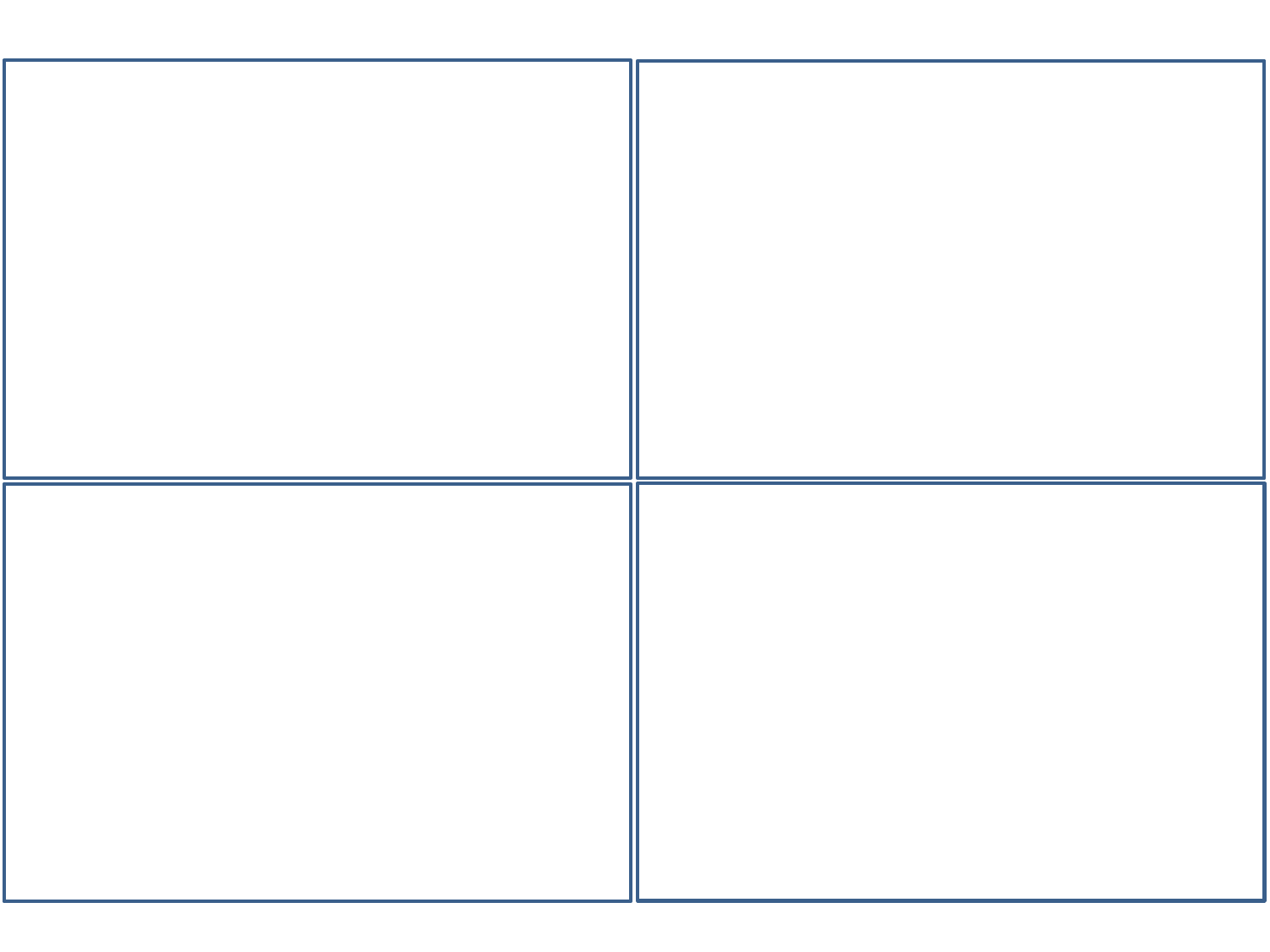

## Slide 6
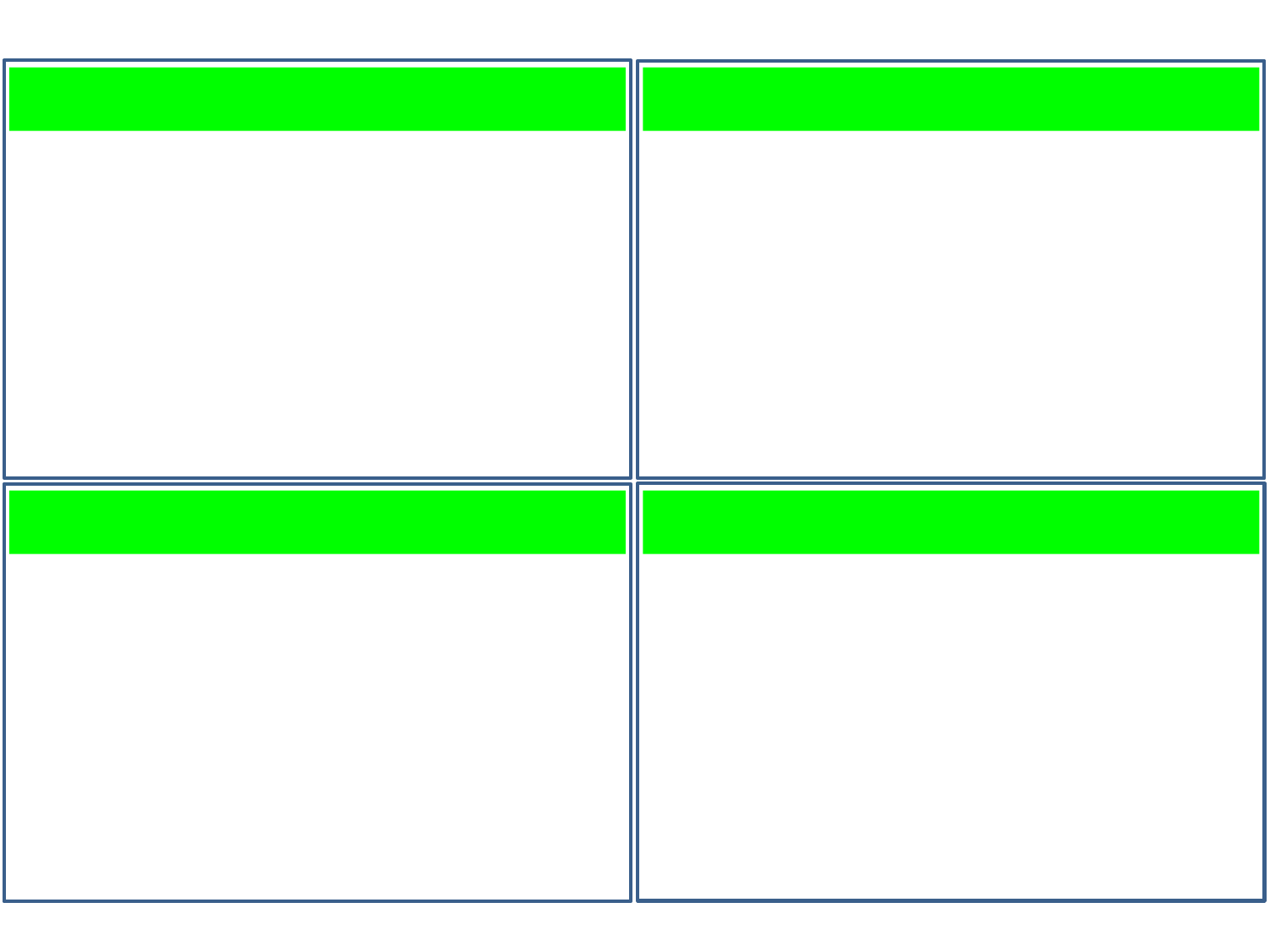

## Slide 7
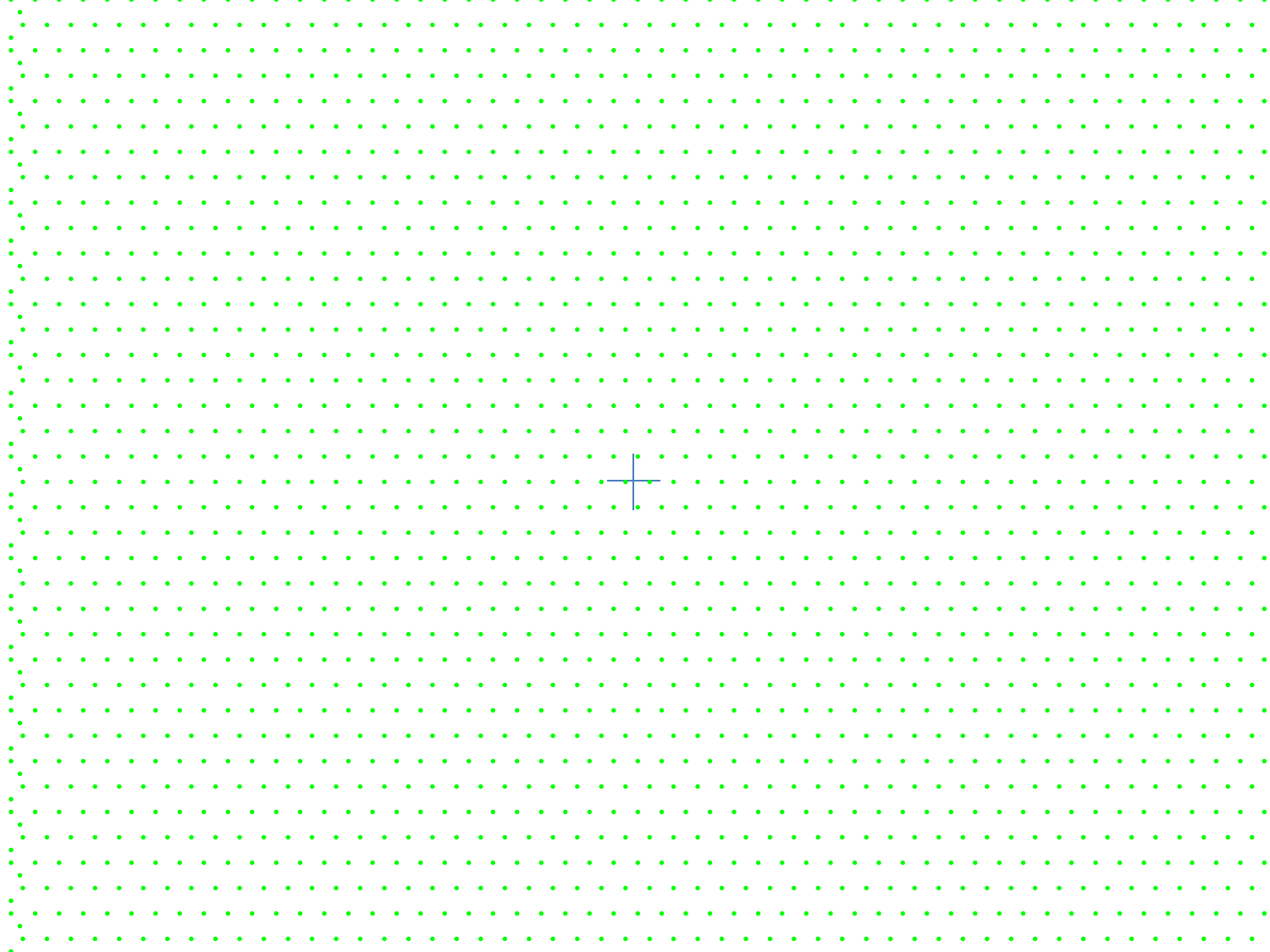

● ● ● ● ● ● ● ● ● ● ● ● ● ● ● ● ● ● ● ● ● ● ● ● ● ● ● ● ● ● ● ● ● ● ● ● ● ● ● ● ● ● ● ● ● ● ● ● ● ● ● ● ● ●
 ● ● ● ● ● ● ● ● ● ● ● ● ● ● ● ● ● ● ● ● ● ● ● ● ● ● ● ● ● ● ● ● ● ● ● ● ● ● ● ● ● ● ● ● ● ● ● ● ● ● ● ● ●
● ● ● ● ● ● ● ● ● ● ● ● ● ● ● ● ● ● ● ● ● ● ● ● ● ● ● ● ● ● ● ● ● ● ● ● ● ● ● ● ● ● ● ● ● ● ● ● ● ● ● ● ● ●
 ● ● ● ● ● ● ● ● ● ● ● ● ● ● ● ● ● ● ● ● ● ● ● ● ● ● ● ● ● ● ● ● ● ● ● ● ● ● ● ● ● ● ● ● ● ● ● ● ● ● ● ● ●
● ● ● ● ● ● ● ● ● ● ● ● ● ● ● ● ● ● ● ● ● ● ● ● ● ● ● ● ● ● ● ● ● ● ● ● ● ● ● ● ● ● ● ● ● ● ● ● ● ● ● ● ● ●
 ● ● ● ● ● ● ● ● ● ● ● ● ● ● ● ● ● ● ● ● ● ● ● ● ● ● ● ● ● ● ● ● ● ● ● ● ● ● ● ● ● ● ● ● ● ● ● ● ● ● ● ● ●
● ● ● ● ● ● ● ● ● ● ● ● ● ● ● ● ● ● ● ● ● ● ● ● ● ● ● ● ● ● ● ● ● ● ● ● ● ● ● ● ● ● ● ● ● ● ● ● ● ● ● ● ● ●
 ● ● ● ● ● ● ● ● ● ● ● ● ● ● ● ● ● ● ● ● ● ● ● ● ● ● ● ● ● ● ● ● ● ● ● ● ● ● ● ● ● ● ● ● ● ● ● ● ● ● ● ● ●
● ● ● ● ● ● ● ● ● ● ● ● ● ● ● ● ● ● ● ● ● ● ● ● ● ● ● ● ● ● ● ● ● ● ● ● ● ● ● ● ● ● ● ● ● ● ● ● ● ● ● ● ● ●
 ● ● ● ● ● ● ● ● ● ● ● ● ● ● ● ● ● ● ● ● ● ● ● ● ● ● ● ● ● ● ● ● ● ● ● ● ● ● ● ● ● ● ● ● ● ● ● ● ● ● ● ● ●
● ● ● ● ● ● ● ● ● ● ● ● ● ● ● ● ● ● ● ● ● ● ● ● ● ● ● ● ● ● ● ● ● ● ● ● ● ● ● ● ● ● ● ● ● ● ● ● ● ● ● ● ● ●
 ● ● ● ● ● ● ● ● ● ● ● ● ● ● ● ● ● ● ● ● ● ● ● ● ● ● ● ● ● ● ● ● ● ● ● ● ● ● ● ● ● ● ● ● ● ● ● ● ● ● ● ● ●
● ● ● ● ● ● ● ● ● ● ● ● ● ● ● ● ● ● ● ● ● ● ● ● ● ● ● ● ● ● ● ● ● ● ● ● ● ● ● ● ● ● ● ● ● ● ● ● ● ● ● ● ● ●
 ● ● ● ● ● ● ● ● ● ● ● ● ● ● ● ● ● ● ● ● ● ● ● ● ● ● ● ● ● ● ● ● ● ● ● ● ● ● ● ● ● ● ● ● ● ● ● ● ● ● ● ● ●
● ● ● ● ● ● ● ● ● ● ● ● ● ● ● ● ● ● ● ● ● ● ● ● ● ● ● ● ● ● ● ● ● ● ● ● ● ● ● ● ● ● ● ● ● ● ● ● ● ● ● ● ● ●
 ● ● ● ● ● ● ● ● ● ● ● ● ● ● ● ● ● ● ● ● ● ● ● ● ● ● ● ● ● ● ● ● ● ● ● ● ● ● ● ● ● ● ● ● ● ● ● ● ● ● ● ● ●
● ● ● ● ● ● ● ● ● ● ● ● ● ● ● ● ● ● ● ● ● ● ● ● ● ● ● ● ● ● ● ● ● ● ● ● ● ● ● ● ● ● ● ● ● ● ● ● ● ● ● ● ● ●
 ● ● ● ● ● ● ● ● ● ● ● ● ● ● ● ● ● ● ● ● ● ● ● ● ● ● ● ● ● ● ● ● ● ● ● ● ● ● ● ● ● ● ● ● ● ● ● ● ● ● ● ● ●
● ● ● ● ● ● ● ● ● ● ● ● ● ● ● ● ● ● ● ● ● ● ● ● ● ● ● ● ● ● ● ● ● ● ● ● ● ● ● ● ● ● ● ● ● ● ● ● ● ● ● ● ● ●
 ● ● ● ● ● ● ● ● ● ● ● ● ● ● ● ● ● ● ● ● ● ● ● ● ● ● ● ● ● ● ● ● ● ● ● ● ● ● ● ● ● ● ● ● ● ● ● ● ● ● ● ● ●
● ● ● ● ● ● ● ● ● ● ● ● ● ● ● ● ● ● ● ● ● ● ● ● ● ● ● ● ● ● ● ● ● ● ● ● ● ● ● ● ● ● ● ● ● ● ● ● ● ● ● ● ● ●
 ● ● ● ● ● ● ● ● ● ● ● ● ● ● ● ● ● ● ● ● ● ● ● ● ● ● ● ● ● ● ● ● ● ● ● ● ● ● ● ● ● ● ● ● ● ● ● ● ● ● ● ● ●
● ● ● ● ● ● ● ● ● ● ● ● ● ● ● ● ● ● ● ● ● ● ● ● ● ● ● ● ● ● ● ● ● ● ● ● ● ● ● ● ● ● ● ● ● ● ● ● ● ● ● ● ● ●
 ● ● ● ● ● ● ● ● ● ● ● ● ● ● ● ● ● ● ● ● ● ● ● ● ● ● ● ● ● ● ● ● ● ● ● ● ● ● ● ● ● ● ● ● ● ● ● ● ● ● ● ● ●
● ● ● ● ● ● ● ● ● ● ● ● ● ● ● ● ● ● ● ● ● ● ● ● ● ● ● ● ● ● ● ● ● ● ● ● ● ● ● ● ● ● ● ● ● ● ● ● ● ● ● ● ● ●
 ● ● ● ● ● ● ● ● ● ● ● ● ● ● ● ● ● ● ● ● ● ● ● ● ● ● ● ● ● ● ● ● ● ● ● ● ● ● ● ● ● ● ● ● ● ● ● ● ● ● ● ● ●
● ● ● ● ● ● ● ● ● ● ● ● ● ● ● ● ● ● ● ● ● ● ● ● ● ● ● ● ● ● ● ● ● ● ● ● ● ● ● ● ● ● ● ● ● ● ● ● ● ● ● ● ● ●
 ● ● ● ● ● ● ● ● ● ● ● ● ● ● ● ● ● ● ● ● ● ● ● ● ● ● ● ● ● ● ● ● ● ● ● ● ● ● ● ● ● ● ● ● ● ● ● ● ● ● ● ● ●
● ● ● ● ● ● ● ● ● ● ● ● ● ● ● ● ● ● ● ● ● ● ● ● ● ● ● ● ● ● ● ● ● ● ● ● ● ● ● ● ● ● ● ● ● ● ● ● ● ● ● ● ● ●
 ● ● ● ● ● ● ● ● ● ● ● ● ● ● ● ● ● ● ● ● ● ● ● ● ● ● ● ● ● ● ● ● ● ● ● ● ● ● ● ● ● ● ● ● ● ● ● ● ● ● ● ● ●
● ● ● ● ● ● ● ● ● ● ● ● ● ● ● ● ● ● ● ● ● ● ● ● ● ● ● ● ● ● ● ● ● ● ● ● ● ● ● ● ● ● ● ● ● ● ● ● ● ● ● ● ● ●
 ● ● ● ● ● ● ● ● ● ● ● ● ● ● ● ● ● ● ● ● ● ● ● ● ● ● ● ● ● ● ● ● ● ● ● ● ● ● ● ● ● ● ● ● ● ● ● ● ● ● ● ● ●
● ● ● ● ● ● ● ● ● ● ● ● ● ● ● ● ● ● ● ● ● ● ● ● ● ● ● ● ● ● ● ● ● ● ● ● ● ● ● ● ● ● ● ● ● ● ● ● ● ● ● ● ● ●
 ● ● ● ● ● ● ● ● ● ● ● ● ● ● ● ● ● ● ● ● ● ● ● ● ● ● ● ● ● ● ● ● ● ● ● ● ● ● ● ● ● ● ● ● ● ● ● ● ● ● ● ● ●
● ● ● ● ● ● ● ● ● ● ● ● ● ● ● ● ● ● ● ● ● ● ● ● ● ● ● ● ● ● ● ● ● ● ● ● ● ● ● ● ● ● ● ● ● ● ● ● ● ● ● ● ● ●
 ● ● ● ● ● ● ● ● ● ● ● ● ● ● ● ● ● ● ● ● ● ● ● ● ● ● ● ● ● ● ● ● ● ● ● ● ● ● ● ● ● ● ● ● ● ● ● ● ● ● ● ● ●
● ● ● ● ● ● ● ● ● ● ● ● ● ● ● ● ● ● ● ● ● ● ● ● ● ● ● ● ● ● ● ● ● ● ● ● ● ● ● ● ● ● ● ● ● ● ● ● ● ● ● ● ● ●
 ● ● ● ● ● ● ● ● ● ● ● ● ● ● ● ● ● ● ● ● ● ● ● ● ● ● ● ● ● ● ● ● ● ● ● ● ● ● ● ● ● ● ● ● ● ● ● ● ● ● ● ● ●
● ● ● ● ● ● ● ● ● ● ● ● ● ● ● ● ● ● ● ● ● ● ● ● ● ● ● ● ● ● ● ● ● ● ● ● ● ● ● ● ● ● ● ● ● ● ● ● ● ● ● ● ● ●
 ● ● ● ● ● ● ● ● ● ● ● ● ● ● ● ● ● ● ● ● ● ● ● ● ● ● ● ● ● ● ● ● ● ● ● ● ● ● ● ● ● ● ● ● ● ● ● ● ● ● ● ● ●
● ● ● ● ● ● ● ● ● ● ● ● ● ● ● ● ● ● ● ● ● ● ● ● ● ● ● ● ● ● ● ● ● ● ● ● ● ● ● ● ● ● ● ● ● ● ● ● ● ● ● ● ● ●
 ● ● ● ● ● ● ● ● ● ● ● ● ● ● ● ● ● ● ● ● ● ● ● ● ● ● ● ● ● ● ● ● ● ● ● ● ● ● ● ● ● ● ● ● ● ● ● ● ● ● ● ● ●
● ● ● ● ● ● ● ● ● ● ● ● ● ● ● ● ● ● ● ● ● ● ● ● ● ● ● ● ● ● ● ● ● ● ● ● ● ● ● ● ● ● ● ● ● ● ● ● ● ● ● ● ● ●
 ● ● ● ● ● ● ● ● ● ● ● ● ● ● ● ● ● ● ● ● ● ● ● ● ● ● ● ● ● ● ● ● ● ● ● ● ● ● ● ● ● ● ● ● ● ● ● ● ● ● ● ● ●
● ● ● ● ● ● ● ● ● ● ● ● ● ● ● ● ● ● ● ● ● ● ● ● ● ● ● ● ● ● ● ● ● ● ● ● ● ● ● ● ● ● ● ● ● ● ● ● ● ● ● ● ● ●
 ● ● ● ● ● ● ● ● ● ● ● ● ● ● ● ● ● ● ● ● ● ● ● ● ● ● ● ● ● ● ● ● ● ● ● ● ● ● ● ● ● ● ● ● ● ● ● ● ● ● ● ● ●
● ● ● ● ● ● ● ● ● ● ● ● ● ● ● ● ● ● ● ● ● ● ● ● ● ● ● ● ● ● ● ● ● ● ● ● ● ● ● ● ● ● ● ● ● ● ● ● ● ● ● ● ● ●
 ● ● ● ● ● ● ● ● ● ● ● ● ● ● ● ● ● ● ● ● ● ● ● ● ● ● ● ● ● ● ● ● ● ● ● ● ● ● ● ● ● ● ● ● ● ● ● ● ● ● ● ● ●
● ● ● ● ● ● ● ● ● ● ● ● ● ● ● ● ● ● ● ● ● ● ● ● ● ● ● ● ● ● ● ● ● ● ● ● ● ● ● ● ● ● ● ● ● ● ● ● ● ● ● ● ● ●
 ● ● ● ● ● ● ● ● ● ● ● ● ● ● ● ● ● ● ● ● ● ● ● ● ● ● ● ● ● ● ● ● ● ● ● ● ● ● ● ● ● ● ● ● ● ● ● ● ● ● ● ● ●
● ● ● ● ● ● ● ● ● ● ● ● ● ● ● ● ● ● ● ● ● ● ● ● ● ● ● ● ● ● ● ● ● ● ● ● ● ● ● ● ● ● ● ● ● ● ● ● ● ● ● ● ● ●
 ● ● ● ● ● ● ● ● ● ● ● ● ● ● ● ● ● ● ● ● ● ● ● ● ● ● ● ● ● ● ● ● ● ● ● ● ● ● ● ● ● ● ● ● ● ● ● ● ● ● ● ● ●
● ● ● ● ● ● ● ● ● ● ● ● ● ● ● ● ● ● ● ● ● ● ● ● ● ● ● ● ● ● ● ● ● ● ● ● ● ● ● ● ● ● ● ● ● ● ● ● ● ● ● ● ● ●
 ● ● ● ● ● ● ● ● ● ● ● ● ● ● ● ● ● ● ● ● ● ● ● ● ● ● ● ● ● ● ● ● ● ● ● ● ● ● ● ● ● ● ● ● ● ● ● ● ● ● ● ● ●
● ● ● ● ● ● ● ● ● ● ● ● ● ● ● ● ● ● ● ● ● ● ● ● ● ● ● ● ● ● ● ● ● ● ● ● ● ● ● ● ● ● ● ● ● ● ● ● ● ● ● ● ● ●
 ● ● ● ● ● ● ● ● ● ● ● ● ● ● ● ● ● ● ● ● ● ● ● ● ● ● ● ● ● ● ● ● ● ● ● ● ● ● ● ● ● ● ● ● ● ● ● ● ● ● ● ● ●
● ● ● ● ● ● ● ● ● ● ● ● ● ● ● ● ● ● ● ● ● ● ● ● ● ● ● ● ● ● ● ● ● ● ● ● ● ● ● ● ● ● ● ● ● ● ● ● ● ● ● ● ● ●
 ● ● ● ● ● ● ● ● ● ● ● ● ● ● ● ● ● ● ● ● ● ● ● ● ● ● ● ● ● ● ● ● ● ● ● ● ● ● ● ● ● ● ● ● ● ● ● ● ● ● ● ● ●
● ● ● ● ● ● ● ● ● ● ● ● ● ● ● ● ● ● ● ● ● ● ● ● ● ● ● ● ● ● ● ● ● ● ● ● ● ● ● ● ● ● ● ● ● ● ● ● ● ● ● ● ● ●
 ● ● ● ● ● ● ● ● ● ● ● ● ● ● ● ● ● ● ● ● ● ● ● ● ● ● ● ● ● ● ● ● ● ● ● ● ● ● ● ● ● ● ● ● ● ● ● ● ● ● ● ● ●
● ● ● ● ● ● ● ● ● ● ● ● ● ● ● ● ● ● ● ● ● ● ● ● ● ● ● ● ● ● ● ● ● ● ● ● ● ● ● ● ● ● ● ● ● ● ● ● ● ● ● ● ● ●
 ● ● ● ● ● ● ● ● ● ● ● ● ● ● ● ● ● ● ● ● ● ● ● ● ● ● ● ● ● ● ● ● ● ● ● ● ● ● ● ● ● ● ● ● ● ● ● ● ● ● ● ● ●
● ● ● ● ● ● ● ● ● ● ● ● ● ● ● ● ● ● ● ● ● ● ● ● ● ● ● ● ● ● ● ● ● ● ● ● ● ● ● ● ● ● ● ● ● ● ● ● ● ● ● ● ● ●
 ● ● ● ● ● ● ● ● ● ● ● ● ● ● ● ● ● ● ● ● ● ● ● ● ● ● ● ● ● ● ● ● ● ● ● ● ● ● ● ● ● ● ● ● ● ● ● ● ● ● ● ● ●
● ● ● ● ● ● ● ● ● ● ● ● ● ● ● ● ● ● ● ● ● ● ● ● ● ● ● ● ● ● ● ● ● ● ● ● ● ● ● ● ● ● ● ● ● ● ● ● ● ● ● ● ● ●
 ● ● ● ● ● ● ● ● ● ● ● ● ● ● ● ● ● ● ● ● ● ● ● ● ● ● ● ● ● ● ● ● ● ● ● ● ● ● ● ● ● ● ● ● ● ● ● ● ● ● ● ● ●
● ● ● ● ● ● ● ● ● ● ● ● ● ● ● ● ● ● ● ● ● ● ● ● ● ● ● ● ● ● ● ● ● ● ● ● ● ● ● ● ● ● ● ● ● ● ● ● ● ● ● ● ● ●
 ● ● ● ● ● ● ● ● ● ● ● ● ● ● ● ● ● ● ● ● ● ● ● ● ● ● ● ● ● ● ● ● ● ● ● ● ● ● ● ● ● ● ● ● ● ● ● ● ● ● ● ● ●
● ● ● ● ● ● ● ● ● ● ● ● ● ● ● ● ● ● ● ● ● ● ● ● ● ● ● ● ● ● ● ● ● ● ● ● ● ● ● ● ● ● ● ● ● ● ● ● ● ● ● ● ● ●
 ● ● ● ● ● ● ● ● ● ● ● ● ● ● ● ● ● ● ● ● ● ● ● ● ● ● ● ● ● ● ● ● ● ● ● ● ● ● ● ● ● ● ● ● ● ● ● ● ● ● ● ● ●
● ● ● ● ● ● ● ● ● ● ● ● ● ● ● ● ● ● ● ● ● ● ● ● ● ● ● ● ● ● ● ● ● ● ● ● ● ● ● ● ● ● ● ● ● ● ● ● ● ● ● ● ● ●
 ● ● ● ● ● ● ● ● ● ● ● ● ● ● ● ● ● ● ● ● ● ● ● ● ● ● ● ● ● ● ● ● ● ● ● ● ● ● ● ● ● ● ● ● ● ● ● ● ● ● ● ● ●
● ● ● ● ● ● ● ● ● ● ● ● ● ● ● ● ● ● ● ● ● ● ● ● ● ● ● ● ● ● ● ● ● ● ● ● ● ● ● ● ● ● ● ● ● ● ● ● ● ● ● ● ● ●
 ● ● ● ● ● ● ● ● ● ● ● ● ● ● ● ● ● ● ● ● ● ● ● ● ● ● ● ● ● ● ● ● ● ● ● ● ● ● ● ● ● ● ● ● ● ● ● ● ● ● ● ● ●
● ● ● ● ● ● ● ● ● ● ● ● ● ● ● ● ● ● ● ● ● ● ● ● ● ● ● ● ● ● ● ● ● ● ● ● ● ● ● ● ● ● ● ● ● ● ● ● ● ● ● ● ● ●
 ● ● ● ● ● ● ● ● ● ● ● ● ● ● ● ● ● ● ● ● ● ● ● ● ● ● ● ● ● ● ● ● ● ● ● ● ● ● ● ● ● ● ● ● ● ● ● ● ● ● ● ● ●

## Slide 8
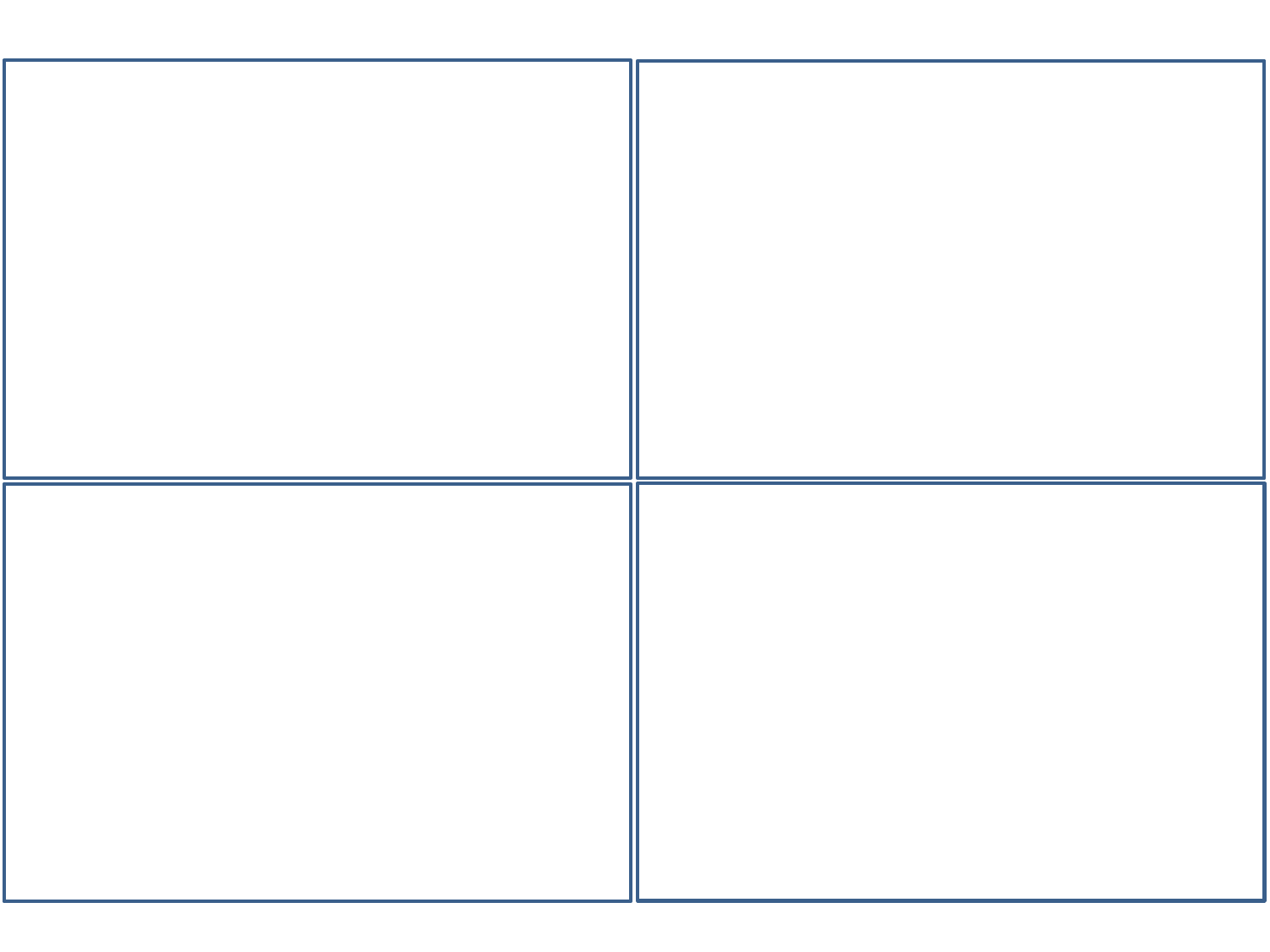

## Slide 9
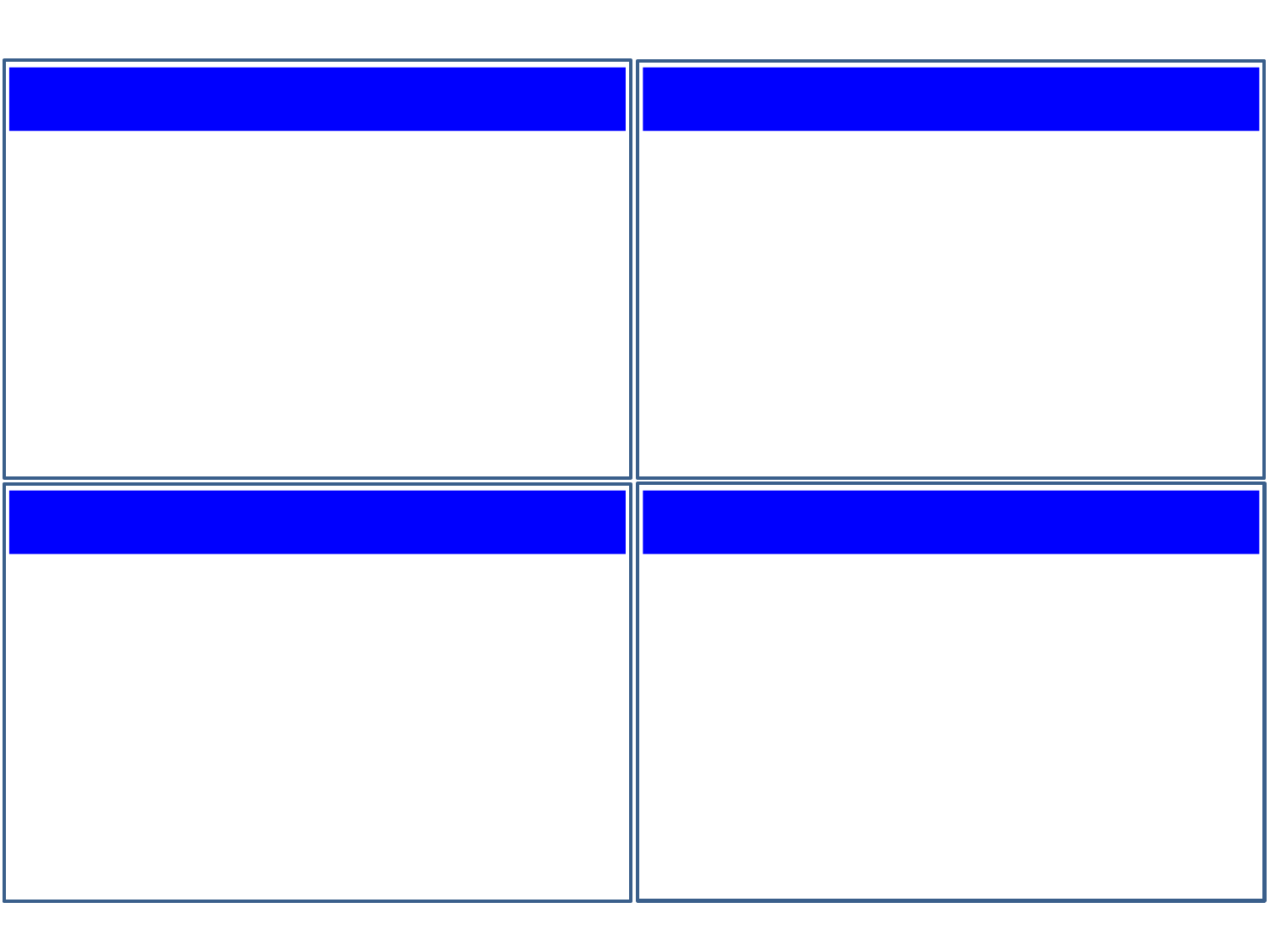

## Slide 10
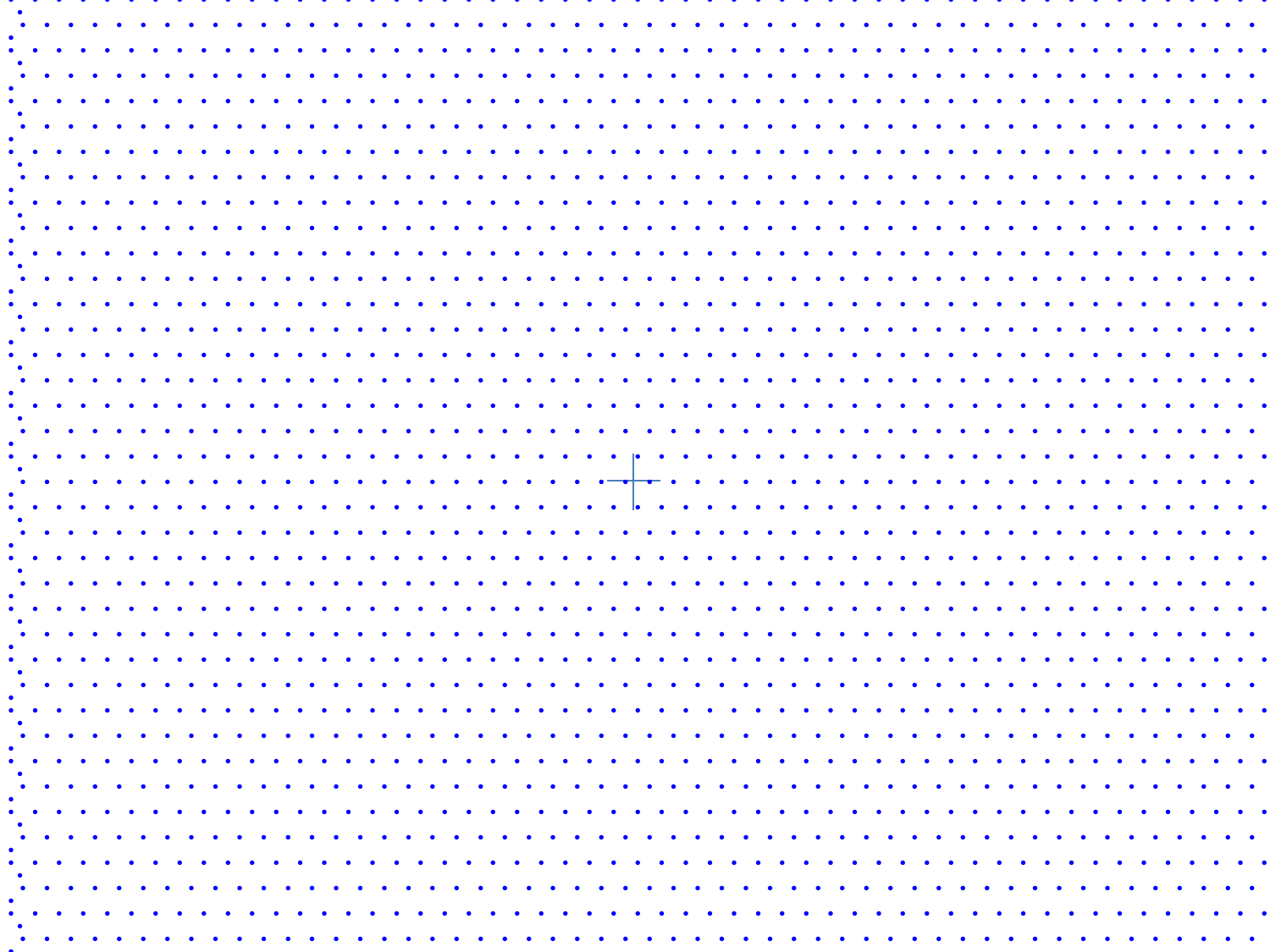

● ● ● ● ● ● ● ● ● ● ● ● ● ● ● ● ● ● ● ● ● ● ● ● ● ● ● ● ● ● ● ● ● ● ● ● ● ● ● ● ● ● ● ● ● ● ● ● ● ● ● ● ● ●
 ● ● ● ● ● ● ● ● ● ● ● ● ● ● ● ● ● ● ● ● ● ● ● ● ● ● ● ● ● ● ● ● ● ● ● ● ● ● ● ● ● ● ● ● ● ● ● ● ● ● ● ● ●
● ● ● ● ● ● ● ● ● ● ● ● ● ● ● ● ● ● ● ● ● ● ● ● ● ● ● ● ● ● ● ● ● ● ● ● ● ● ● ● ● ● ● ● ● ● ● ● ● ● ● ● ● ●
 ● ● ● ● ● ● ● ● ● ● ● ● ● ● ● ● ● ● ● ● ● ● ● ● ● ● ● ● ● ● ● ● ● ● ● ● ● ● ● ● ● ● ● ● ● ● ● ● ● ● ● ● ●
● ● ● ● ● ● ● ● ● ● ● ● ● ● ● ● ● ● ● ● ● ● ● ● ● ● ● ● ● ● ● ● ● ● ● ● ● ● ● ● ● ● ● ● ● ● ● ● ● ● ● ● ● ●
 ● ● ● ● ● ● ● ● ● ● ● ● ● ● ● ● ● ● ● ● ● ● ● ● ● ● ● ● ● ● ● ● ● ● ● ● ● ● ● ● ● ● ● ● ● ● ● ● ● ● ● ● ●
● ● ● ● ● ● ● ● ● ● ● ● ● ● ● ● ● ● ● ● ● ● ● ● ● ● ● ● ● ● ● ● ● ● ● ● ● ● ● ● ● ● ● ● ● ● ● ● ● ● ● ● ● ●
 ● ● ● ● ● ● ● ● ● ● ● ● ● ● ● ● ● ● ● ● ● ● ● ● ● ● ● ● ● ● ● ● ● ● ● ● ● ● ● ● ● ● ● ● ● ● ● ● ● ● ● ● ●
● ● ● ● ● ● ● ● ● ● ● ● ● ● ● ● ● ● ● ● ● ● ● ● ● ● ● ● ● ● ● ● ● ● ● ● ● ● ● ● ● ● ● ● ● ● ● ● ● ● ● ● ● ●
 ● ● ● ● ● ● ● ● ● ● ● ● ● ● ● ● ● ● ● ● ● ● ● ● ● ● ● ● ● ● ● ● ● ● ● ● ● ● ● ● ● ● ● ● ● ● ● ● ● ● ● ● ●
● ● ● ● ● ● ● ● ● ● ● ● ● ● ● ● ● ● ● ● ● ● ● ● ● ● ● ● ● ● ● ● ● ● ● ● ● ● ● ● ● ● ● ● ● ● ● ● ● ● ● ● ● ●
 ● ● ● ● ● ● ● ● ● ● ● ● ● ● ● ● ● ● ● ● ● ● ● ● ● ● ● ● ● ● ● ● ● ● ● ● ● ● ● ● ● ● ● ● ● ● ● ● ● ● ● ● ●
● ● ● ● ● ● ● ● ● ● ● ● ● ● ● ● ● ● ● ● ● ● ● ● ● ● ● ● ● ● ● ● ● ● ● ● ● ● ● ● ● ● ● ● ● ● ● ● ● ● ● ● ● ●
 ● ● ● ● ● ● ● ● ● ● ● ● ● ● ● ● ● ● ● ● ● ● ● ● ● ● ● ● ● ● ● ● ● ● ● ● ● ● ● ● ● ● ● ● ● ● ● ● ● ● ● ● ●
● ● ● ● ● ● ● ● ● ● ● ● ● ● ● ● ● ● ● ● ● ● ● ● ● ● ● ● ● ● ● ● ● ● ● ● ● ● ● ● ● ● ● ● ● ● ● ● ● ● ● ● ● ●
 ● ● ● ● ● ● ● ● ● ● ● ● ● ● ● ● ● ● ● ● ● ● ● ● ● ● ● ● ● ● ● ● ● ● ● ● ● ● ● ● ● ● ● ● ● ● ● ● ● ● ● ● ●
● ● ● ● ● ● ● ● ● ● ● ● ● ● ● ● ● ● ● ● ● ● ● ● ● ● ● ● ● ● ● ● ● ● ● ● ● ● ● ● ● ● ● ● ● ● ● ● ● ● ● ● ● ●
 ● ● ● ● ● ● ● ● ● ● ● ● ● ● ● ● ● ● ● ● ● ● ● ● ● ● ● ● ● ● ● ● ● ● ● ● ● ● ● ● ● ● ● ● ● ● ● ● ● ● ● ● ●
● ● ● ● ● ● ● ● ● ● ● ● ● ● ● ● ● ● ● ● ● ● ● ● ● ● ● ● ● ● ● ● ● ● ● ● ● ● ● ● ● ● ● ● ● ● ● ● ● ● ● ● ● ●
 ● ● ● ● ● ● ● ● ● ● ● ● ● ● ● ● ● ● ● ● ● ● ● ● ● ● ● ● ● ● ● ● ● ● ● ● ● ● ● ● ● ● ● ● ● ● ● ● ● ● ● ● ●
● ● ● ● ● ● ● ● ● ● ● ● ● ● ● ● ● ● ● ● ● ● ● ● ● ● ● ● ● ● ● ● ● ● ● ● ● ● ● ● ● ● ● ● ● ● ● ● ● ● ● ● ● ●
 ● ● ● ● ● ● ● ● ● ● ● ● ● ● ● ● ● ● ● ● ● ● ● ● ● ● ● ● ● ● ● ● ● ● ● ● ● ● ● ● ● ● ● ● ● ● ● ● ● ● ● ● ●
● ● ● ● ● ● ● ● ● ● ● ● ● ● ● ● ● ● ● ● ● ● ● ● ● ● ● ● ● ● ● ● ● ● ● ● ● ● ● ● ● ● ● ● ● ● ● ● ● ● ● ● ● ●
 ● ● ● ● ● ● ● ● ● ● ● ● ● ● ● ● ● ● ● ● ● ● ● ● ● ● ● ● ● ● ● ● ● ● ● ● ● ● ● ● ● ● ● ● ● ● ● ● ● ● ● ● ●
● ● ● ● ● ● ● ● ● ● ● ● ● ● ● ● ● ● ● ● ● ● ● ● ● ● ● ● ● ● ● ● ● ● ● ● ● ● ● ● ● ● ● ● ● ● ● ● ● ● ● ● ● ●
 ● ● ● ● ● ● ● ● ● ● ● ● ● ● ● ● ● ● ● ● ● ● ● ● ● ● ● ● ● ● ● ● ● ● ● ● ● ● ● ● ● ● ● ● ● ● ● ● ● ● ● ● ●
● ● ● ● ● ● ● ● ● ● ● ● ● ● ● ● ● ● ● ● ● ● ● ● ● ● ● ● ● ● ● ● ● ● ● ● ● ● ● ● ● ● ● ● ● ● ● ● ● ● ● ● ● ●
 ● ● ● ● ● ● ● ● ● ● ● ● ● ● ● ● ● ● ● ● ● ● ● ● ● ● ● ● ● ● ● ● ● ● ● ● ● ● ● ● ● ● ● ● ● ● ● ● ● ● ● ● ●
● ● ● ● ● ● ● ● ● ● ● ● ● ● ● ● ● ● ● ● ● ● ● ● ● ● ● ● ● ● ● ● ● ● ● ● ● ● ● ● ● ● ● ● ● ● ● ● ● ● ● ● ● ●
 ● ● ● ● ● ● ● ● ● ● ● ● ● ● ● ● ● ● ● ● ● ● ● ● ● ● ● ● ● ● ● ● ● ● ● ● ● ● ● ● ● ● ● ● ● ● ● ● ● ● ● ● ●
● ● ● ● ● ● ● ● ● ● ● ● ● ● ● ● ● ● ● ● ● ● ● ● ● ● ● ● ● ● ● ● ● ● ● ● ● ● ● ● ● ● ● ● ● ● ● ● ● ● ● ● ● ●
 ● ● ● ● ● ● ● ● ● ● ● ● ● ● ● ● ● ● ● ● ● ● ● ● ● ● ● ● ● ● ● ● ● ● ● ● ● ● ● ● ● ● ● ● ● ● ● ● ● ● ● ● ●
● ● ● ● ● ● ● ● ● ● ● ● ● ● ● ● ● ● ● ● ● ● ● ● ● ● ● ● ● ● ● ● ● ● ● ● ● ● ● ● ● ● ● ● ● ● ● ● ● ● ● ● ● ●
 ● ● ● ● ● ● ● ● ● ● ● ● ● ● ● ● ● ● ● ● ● ● ● ● ● ● ● ● ● ● ● ● ● ● ● ● ● ● ● ● ● ● ● ● ● ● ● ● ● ● ● ● ●
● ● ● ● ● ● ● ● ● ● ● ● ● ● ● ● ● ● ● ● ● ● ● ● ● ● ● ● ● ● ● ● ● ● ● ● ● ● ● ● ● ● ● ● ● ● ● ● ● ● ● ● ● ●
 ● ● ● ● ● ● ● ● ● ● ● ● ● ● ● ● ● ● ● ● ● ● ● ● ● ● ● ● ● ● ● ● ● ● ● ● ● ● ● ● ● ● ● ● ● ● ● ● ● ● ● ● ●
● ● ● ● ● ● ● ● ● ● ● ● ● ● ● ● ● ● ● ● ● ● ● ● ● ● ● ● ● ● ● ● ● ● ● ● ● ● ● ● ● ● ● ● ● ● ● ● ● ● ● ● ● ●
 ● ● ● ● ● ● ● ● ● ● ● ● ● ● ● ● ● ● ● ● ● ● ● ● ● ● ● ● ● ● ● ● ● ● ● ● ● ● ● ● ● ● ● ● ● ● ● ● ● ● ● ● ●
● ● ● ● ● ● ● ● ● ● ● ● ● ● ● ● ● ● ● ● ● ● ● ● ● ● ● ● ● ● ● ● ● ● ● ● ● ● ● ● ● ● ● ● ● ● ● ● ● ● ● ● ● ●
 ● ● ● ● ● ● ● ● ● ● ● ● ● ● ● ● ● ● ● ● ● ● ● ● ● ● ● ● ● ● ● ● ● ● ● ● ● ● ● ● ● ● ● ● ● ● ● ● ● ● ● ● ●
● ● ● ● ● ● ● ● ● ● ● ● ● ● ● ● ● ● ● ● ● ● ● ● ● ● ● ● ● ● ● ● ● ● ● ● ● ● ● ● ● ● ● ● ● ● ● ● ● ● ● ● ● ●
 ● ● ● ● ● ● ● ● ● ● ● ● ● ● ● ● ● ● ● ● ● ● ● ● ● ● ● ● ● ● ● ● ● ● ● ● ● ● ● ● ● ● ● ● ● ● ● ● ● ● ● ● ●
● ● ● ● ● ● ● ● ● ● ● ● ● ● ● ● ● ● ● ● ● ● ● ● ● ● ● ● ● ● ● ● ● ● ● ● ● ● ● ● ● ● ● ● ● ● ● ● ● ● ● ● ● ●
 ● ● ● ● ● ● ● ● ● ● ● ● ● ● ● ● ● ● ● ● ● ● ● ● ● ● ● ● ● ● ● ● ● ● ● ● ● ● ● ● ● ● ● ● ● ● ● ● ● ● ● ● ●
● ● ● ● ● ● ● ● ● ● ● ● ● ● ● ● ● ● ● ● ● ● ● ● ● ● ● ● ● ● ● ● ● ● ● ● ● ● ● ● ● ● ● ● ● ● ● ● ● ● ● ● ● ●
 ● ● ● ● ● ● ● ● ● ● ● ● ● ● ● ● ● ● ● ● ● ● ● ● ● ● ● ● ● ● ● ● ● ● ● ● ● ● ● ● ● ● ● ● ● ● ● ● ● ● ● ● ●
● ● ● ● ● ● ● ● ● ● ● ● ● ● ● ● ● ● ● ● ● ● ● ● ● ● ● ● ● ● ● ● ● ● ● ● ● ● ● ● ● ● ● ● ● ● ● ● ● ● ● ● ● ●
 ● ● ● ● ● ● ● ● ● ● ● ● ● ● ● ● ● ● ● ● ● ● ● ● ● ● ● ● ● ● ● ● ● ● ● ● ● ● ● ● ● ● ● ● ● ● ● ● ● ● ● ● ●
● ● ● ● ● ● ● ● ● ● ● ● ● ● ● ● ● ● ● ● ● ● ● ● ● ● ● ● ● ● ● ● ● ● ● ● ● ● ● ● ● ● ● ● ● ● ● ● ● ● ● ● ● ●
 ● ● ● ● ● ● ● ● ● ● ● ● ● ● ● ● ● ● ● ● ● ● ● ● ● ● ● ● ● ● ● ● ● ● ● ● ● ● ● ● ● ● ● ● ● ● ● ● ● ● ● ● ●
● ● ● ● ● ● ● ● ● ● ● ● ● ● ● ● ● ● ● ● ● ● ● ● ● ● ● ● ● ● ● ● ● ● ● ● ● ● ● ● ● ● ● ● ● ● ● ● ● ● ● ● ● ●
 ● ● ● ● ● ● ● ● ● ● ● ● ● ● ● ● ● ● ● ● ● ● ● ● ● ● ● ● ● ● ● ● ● ● ● ● ● ● ● ● ● ● ● ● ● ● ● ● ● ● ● ● ●
● ● ● ● ● ● ● ● ● ● ● ● ● ● ● ● ● ● ● ● ● ● ● ● ● ● ● ● ● ● ● ● ● ● ● ● ● ● ● ● ● ● ● ● ● ● ● ● ● ● ● ● ● ●
 ● ● ● ● ● ● ● ● ● ● ● ● ● ● ● ● ● ● ● ● ● ● ● ● ● ● ● ● ● ● ● ● ● ● ● ● ● ● ● ● ● ● ● ● ● ● ● ● ● ● ● ● ●
● ● ● ● ● ● ● ● ● ● ● ● ● ● ● ● ● ● ● ● ● ● ● ● ● ● ● ● ● ● ● ● ● ● ● ● ● ● ● ● ● ● ● ● ● ● ● ● ● ● ● ● ● ●
 ● ● ● ● ● ● ● ● ● ● ● ● ● ● ● ● ● ● ● ● ● ● ● ● ● ● ● ● ● ● ● ● ● ● ● ● ● ● ● ● ● ● ● ● ● ● ● ● ● ● ● ● ●
● ● ● ● ● ● ● ● ● ● ● ● ● ● ● ● ● ● ● ● ● ● ● ● ● ● ● ● ● ● ● ● ● ● ● ● ● ● ● ● ● ● ● ● ● ● ● ● ● ● ● ● ● ●
 ● ● ● ● ● ● ● ● ● ● ● ● ● ● ● ● ● ● ● ● ● ● ● ● ● ● ● ● ● ● ● ● ● ● ● ● ● ● ● ● ● ● ● ● ● ● ● ● ● ● ● ● ●
● ● ● ● ● ● ● ● ● ● ● ● ● ● ● ● ● ● ● ● ● ● ● ● ● ● ● ● ● ● ● ● ● ● ● ● ● ● ● ● ● ● ● ● ● ● ● ● ● ● ● ● ● ●
 ● ● ● ● ● ● ● ● ● ● ● ● ● ● ● ● ● ● ● ● ● ● ● ● ● ● ● ● ● ● ● ● ● ● ● ● ● ● ● ● ● ● ● ● ● ● ● ● ● ● ● ● ●
● ● ● ● ● ● ● ● ● ● ● ● ● ● ● ● ● ● ● ● ● ● ● ● ● ● ● ● ● ● ● ● ● ● ● ● ● ● ● ● ● ● ● ● ● ● ● ● ● ● ● ● ● ●
 ● ● ● ● ● ● ● ● ● ● ● ● ● ● ● ● ● ● ● ● ● ● ● ● ● ● ● ● ● ● ● ● ● ● ● ● ● ● ● ● ● ● ● ● ● ● ● ● ● ● ● ● ●
● ● ● ● ● ● ● ● ● ● ● ● ● ● ● ● ● ● ● ● ● ● ● ● ● ● ● ● ● ● ● ● ● ● ● ● ● ● ● ● ● ● ● ● ● ● ● ● ● ● ● ● ● ●
 ● ● ● ● ● ● ● ● ● ● ● ● ● ● ● ● ● ● ● ● ● ● ● ● ● ● ● ● ● ● ● ● ● ● ● ● ● ● ● ● ● ● ● ● ● ● ● ● ● ● ● ● ●
● ● ● ● ● ● ● ● ● ● ● ● ● ● ● ● ● ● ● ● ● ● ● ● ● ● ● ● ● ● ● ● ● ● ● ● ● ● ● ● ● ● ● ● ● ● ● ● ● ● ● ● ● ●
 ● ● ● ● ● ● ● ● ● ● ● ● ● ● ● ● ● ● ● ● ● ● ● ● ● ● ● ● ● ● ● ● ● ● ● ● ● ● ● ● ● ● ● ● ● ● ● ● ● ● ● ● ●
● ● ● ● ● ● ● ● ● ● ● ● ● ● ● ● ● ● ● ● ● ● ● ● ● ● ● ● ● ● ● ● ● ● ● ● ● ● ● ● ● ● ● ● ● ● ● ● ● ● ● ● ● ●
 ● ● ● ● ● ● ● ● ● ● ● ● ● ● ● ● ● ● ● ● ● ● ● ● ● ● ● ● ● ● ● ● ● ● ● ● ● ● ● ● ● ● ● ● ● ● ● ● ● ● ● ● ●
● ● ● ● ● ● ● ● ● ● ● ● ● ● ● ● ● ● ● ● ● ● ● ● ● ● ● ● ● ● ● ● ● ● ● ● ● ● ● ● ● ● ● ● ● ● ● ● ● ● ● ● ● ●
 ● ● ● ● ● ● ● ● ● ● ● ● ● ● ● ● ● ● ● ● ● ● ● ● ● ● ● ● ● ● ● ● ● ● ● ● ● ● ● ● ● ● ● ● ● ● ● ● ● ● ● ● ●
● ● ● ● ● ● ● ● ● ● ● ● ● ● ● ● ● ● ● ● ● ● ● ● ● ● ● ● ● ● ● ● ● ● ● ● ● ● ● ● ● ● ● ● ● ● ● ● ● ● ● ● ● ●
 ● ● ● ● ● ● ● ● ● ● ● ● ● ● ● ● ● ● ● ● ● ● ● ● ● ● ● ● ● ● ● ● ● ● ● ● ● ● ● ● ● ● ● ● ● ● ● ● ● ● ● ● ●
● ● ● ● ● ● ● ● ● ● ● ● ● ● ● ● ● ● ● ● ● ● ● ● ● ● ● ● ● ● ● ● ● ● ● ● ● ● ● ● ● ● ● ● ● ● ● ● ● ● ● ● ● ●
 ● ● ● ● ● ● ● ● ● ● ● ● ● ● ● ● ● ● ● ● ● ● ● ● ● ● ● ● ● ● ● ● ● ● ● ● ● ● ● ● ● ● ● ● ● ● ● ● ● ● ● ● ●
● ● ● ● ● ● ● ● ● ● ● ● ● ● ● ● ● ● ● ● ● ● ● ● ● ● ● ● ● ● ● ● ● ● ● ● ● ● ● ● ● ● ● ● ● ● ● ● ● ● ● ● ● ●
 ● ● ● ● ● ● ● ● ● ● ● ● ● ● ● ● ● ● ● ● ● ● ● ● ● ● ● ● ● ● ● ● ● ● ● ● ● ● ● ● ● ● ● ● ● ● ● ● ● ● ● ● ●

## Slide 11
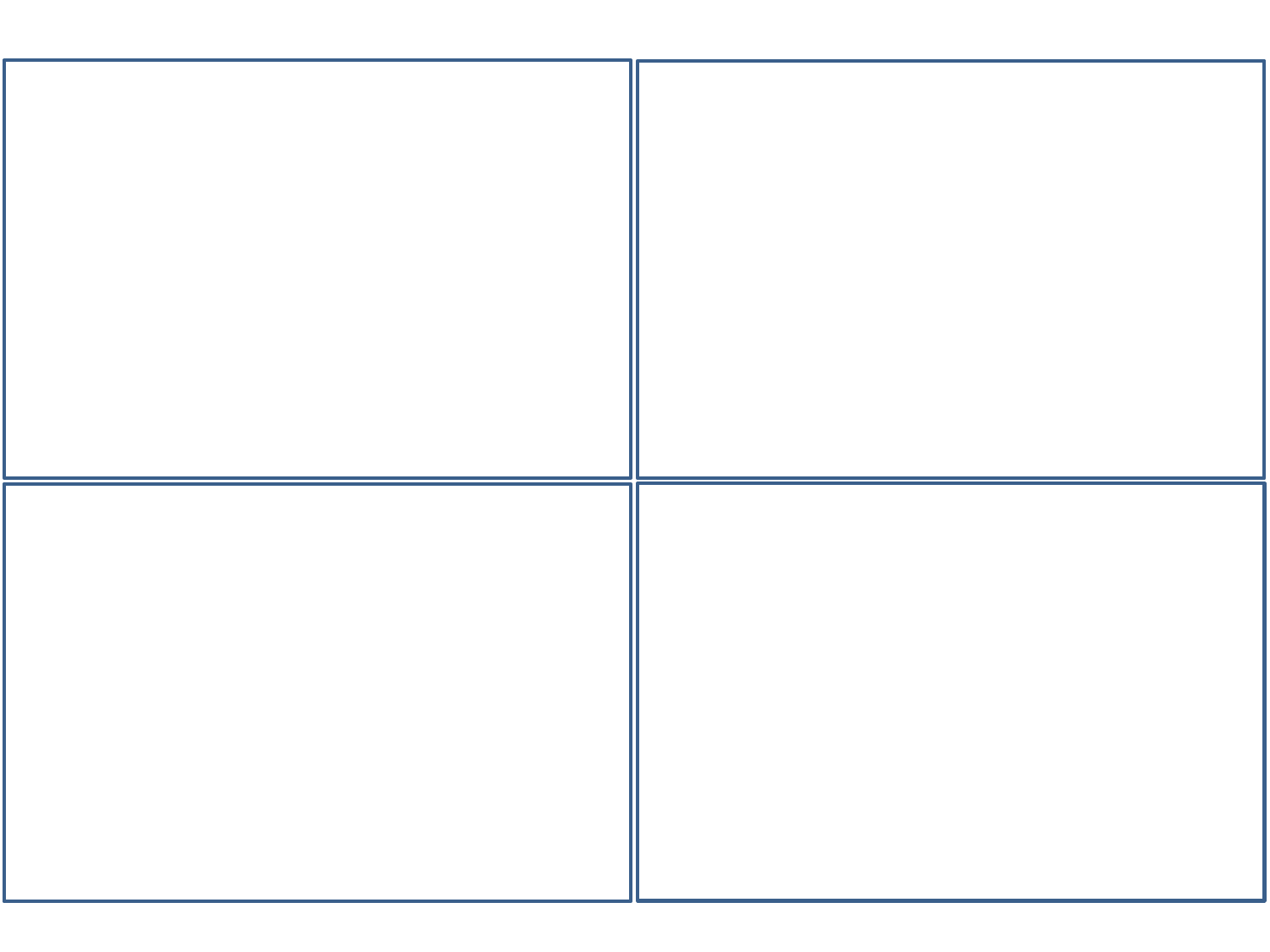

## Slide 12
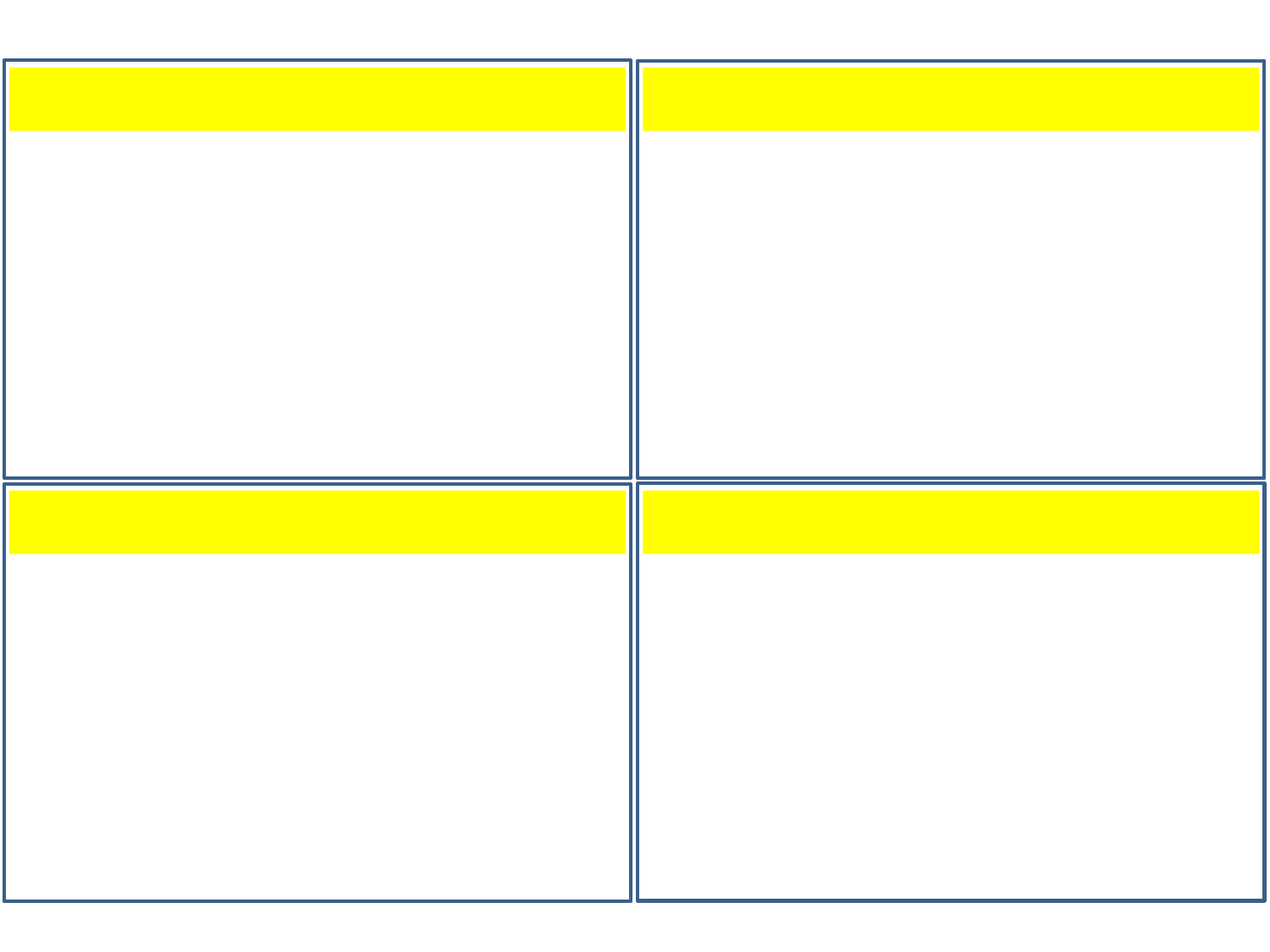

## Slide 13
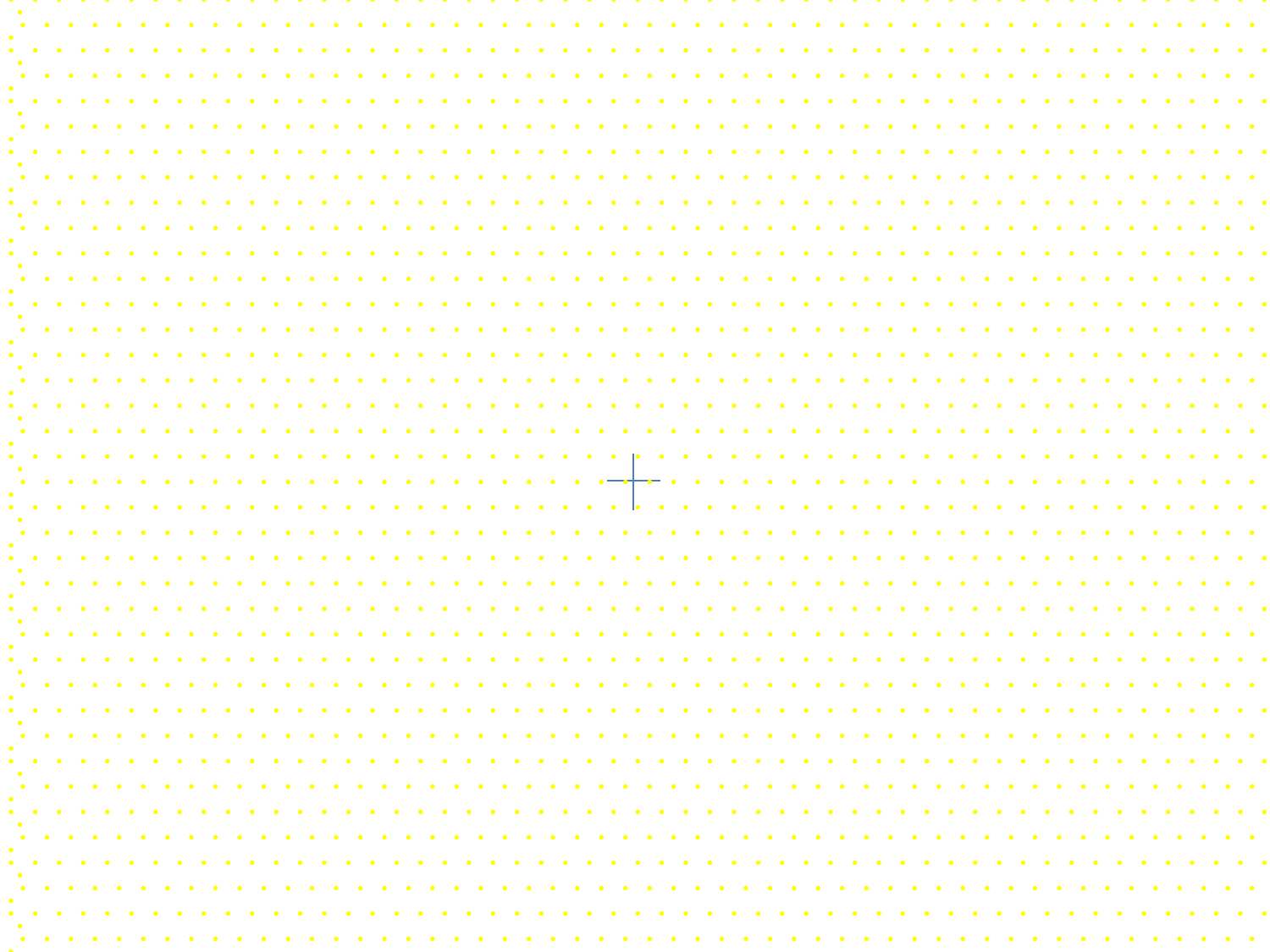

● ● ● ● ● ● ● ● ● ● ● ● ● ● ● ● ● ● ● ● ● ● ● ● ● ● ● ● ● ● ● ● ● ● ● ● ● ● ● ● ● ● ● ● ● ● ● ● ● ● ● ● ● ●
 ● ● ● ● ● ● ● ● ● ● ● ● ● ● ● ● ● ● ● ● ● ● ● ● ● ● ● ● ● ● ● ● ● ● ● ● ● ● ● ● ● ● ● ● ● ● ● ● ● ● ● ● ●
● ● ● ● ● ● ● ● ● ● ● ● ● ● ● ● ● ● ● ● ● ● ● ● ● ● ● ● ● ● ● ● ● ● ● ● ● ● ● ● ● ● ● ● ● ● ● ● ● ● ● ● ● ●
 ● ● ● ● ● ● ● ● ● ● ● ● ● ● ● ● ● ● ● ● ● ● ● ● ● ● ● ● ● ● ● ● ● ● ● ● ● ● ● ● ● ● ● ● ● ● ● ● ● ● ● ● ●
● ● ● ● ● ● ● ● ● ● ● ● ● ● ● ● ● ● ● ● ● ● ● ● ● ● ● ● ● ● ● ● ● ● ● ● ● ● ● ● ● ● ● ● ● ● ● ● ● ● ● ● ● ●
 ● ● ● ● ● ● ● ● ● ● ● ● ● ● ● ● ● ● ● ● ● ● ● ● ● ● ● ● ● ● ● ● ● ● ● ● ● ● ● ● ● ● ● ● ● ● ● ● ● ● ● ● ●
● ● ● ● ● ● ● ● ● ● ● ● ● ● ● ● ● ● ● ● ● ● ● ● ● ● ● ● ● ● ● ● ● ● ● ● ● ● ● ● ● ● ● ● ● ● ● ● ● ● ● ● ● ●
 ● ● ● ● ● ● ● ● ● ● ● ● ● ● ● ● ● ● ● ● ● ● ● ● ● ● ● ● ● ● ● ● ● ● ● ● ● ● ● ● ● ● ● ● ● ● ● ● ● ● ● ● ●
● ● ● ● ● ● ● ● ● ● ● ● ● ● ● ● ● ● ● ● ● ● ● ● ● ● ● ● ● ● ● ● ● ● ● ● ● ● ● ● ● ● ● ● ● ● ● ● ● ● ● ● ● ●
 ● ● ● ● ● ● ● ● ● ● ● ● ● ● ● ● ● ● ● ● ● ● ● ● ● ● ● ● ● ● ● ● ● ● ● ● ● ● ● ● ● ● ● ● ● ● ● ● ● ● ● ● ●
● ● ● ● ● ● ● ● ● ● ● ● ● ● ● ● ● ● ● ● ● ● ● ● ● ● ● ● ● ● ● ● ● ● ● ● ● ● ● ● ● ● ● ● ● ● ● ● ● ● ● ● ● ●
 ● ● ● ● ● ● ● ● ● ● ● ● ● ● ● ● ● ● ● ● ● ● ● ● ● ● ● ● ● ● ● ● ● ● ● ● ● ● ● ● ● ● ● ● ● ● ● ● ● ● ● ● ●
● ● ● ● ● ● ● ● ● ● ● ● ● ● ● ● ● ● ● ● ● ● ● ● ● ● ● ● ● ● ● ● ● ● ● ● ● ● ● ● ● ● ● ● ● ● ● ● ● ● ● ● ● ●
 ● ● ● ● ● ● ● ● ● ● ● ● ● ● ● ● ● ● ● ● ● ● ● ● ● ● ● ● ● ● ● ● ● ● ● ● ● ● ● ● ● ● ● ● ● ● ● ● ● ● ● ● ●
● ● ● ● ● ● ● ● ● ● ● ● ● ● ● ● ● ● ● ● ● ● ● ● ● ● ● ● ● ● ● ● ● ● ● ● ● ● ● ● ● ● ● ● ● ● ● ● ● ● ● ● ● ●
 ● ● ● ● ● ● ● ● ● ● ● ● ● ● ● ● ● ● ● ● ● ● ● ● ● ● ● ● ● ● ● ● ● ● ● ● ● ● ● ● ● ● ● ● ● ● ● ● ● ● ● ● ●
● ● ● ● ● ● ● ● ● ● ● ● ● ● ● ● ● ● ● ● ● ● ● ● ● ● ● ● ● ● ● ● ● ● ● ● ● ● ● ● ● ● ● ● ● ● ● ● ● ● ● ● ● ●
 ● ● ● ● ● ● ● ● ● ● ● ● ● ● ● ● ● ● ● ● ● ● ● ● ● ● ● ● ● ● ● ● ● ● ● ● ● ● ● ● ● ● ● ● ● ● ● ● ● ● ● ● ●
● ● ● ● ● ● ● ● ● ● ● ● ● ● ● ● ● ● ● ● ● ● ● ● ● ● ● ● ● ● ● ● ● ● ● ● ● ● ● ● ● ● ● ● ● ● ● ● ● ● ● ● ● ●
 ● ● ● ● ● ● ● ● ● ● ● ● ● ● ● ● ● ● ● ● ● ● ● ● ● ● ● ● ● ● ● ● ● ● ● ● ● ● ● ● ● ● ● ● ● ● ● ● ● ● ● ● ●
● ● ● ● ● ● ● ● ● ● ● ● ● ● ● ● ● ● ● ● ● ● ● ● ● ● ● ● ● ● ● ● ● ● ● ● ● ● ● ● ● ● ● ● ● ● ● ● ● ● ● ● ● ●
 ● ● ● ● ● ● ● ● ● ● ● ● ● ● ● ● ● ● ● ● ● ● ● ● ● ● ● ● ● ● ● ● ● ● ● ● ● ● ● ● ● ● ● ● ● ● ● ● ● ● ● ● ●
● ● ● ● ● ● ● ● ● ● ● ● ● ● ● ● ● ● ● ● ● ● ● ● ● ● ● ● ● ● ● ● ● ● ● ● ● ● ● ● ● ● ● ● ● ● ● ● ● ● ● ● ● ●
 ● ● ● ● ● ● ● ● ● ● ● ● ● ● ● ● ● ● ● ● ● ● ● ● ● ● ● ● ● ● ● ● ● ● ● ● ● ● ● ● ● ● ● ● ● ● ● ● ● ● ● ● ●
● ● ● ● ● ● ● ● ● ● ● ● ● ● ● ● ● ● ● ● ● ● ● ● ● ● ● ● ● ● ● ● ● ● ● ● ● ● ● ● ● ● ● ● ● ● ● ● ● ● ● ● ● ●
 ● ● ● ● ● ● ● ● ● ● ● ● ● ● ● ● ● ● ● ● ● ● ● ● ● ● ● ● ● ● ● ● ● ● ● ● ● ● ● ● ● ● ● ● ● ● ● ● ● ● ● ● ●
● ● ● ● ● ● ● ● ● ● ● ● ● ● ● ● ● ● ● ● ● ● ● ● ● ● ● ● ● ● ● ● ● ● ● ● ● ● ● ● ● ● ● ● ● ● ● ● ● ● ● ● ● ●
 ● ● ● ● ● ● ● ● ● ● ● ● ● ● ● ● ● ● ● ● ● ● ● ● ● ● ● ● ● ● ● ● ● ● ● ● ● ● ● ● ● ● ● ● ● ● ● ● ● ● ● ● ●
● ● ● ● ● ● ● ● ● ● ● ● ● ● ● ● ● ● ● ● ● ● ● ● ● ● ● ● ● ● ● ● ● ● ● ● ● ● ● ● ● ● ● ● ● ● ● ● ● ● ● ● ● ●
 ● ● ● ● ● ● ● ● ● ● ● ● ● ● ● ● ● ● ● ● ● ● ● ● ● ● ● ● ● ● ● ● ● ● ● ● ● ● ● ● ● ● ● ● ● ● ● ● ● ● ● ● ●
● ● ● ● ● ● ● ● ● ● ● ● ● ● ● ● ● ● ● ● ● ● ● ● ● ● ● ● ● ● ● ● ● ● ● ● ● ● ● ● ● ● ● ● ● ● ● ● ● ● ● ● ● ●
 ● ● ● ● ● ● ● ● ● ● ● ● ● ● ● ● ● ● ● ● ● ● ● ● ● ● ● ● ● ● ● ● ● ● ● ● ● ● ● ● ● ● ● ● ● ● ● ● ● ● ● ● ●
● ● ● ● ● ● ● ● ● ● ● ● ● ● ● ● ● ● ● ● ● ● ● ● ● ● ● ● ● ● ● ● ● ● ● ● ● ● ● ● ● ● ● ● ● ● ● ● ● ● ● ● ● ●
 ● ● ● ● ● ● ● ● ● ● ● ● ● ● ● ● ● ● ● ● ● ● ● ● ● ● ● ● ● ● ● ● ● ● ● ● ● ● ● ● ● ● ● ● ● ● ● ● ● ● ● ● ●
● ● ● ● ● ● ● ● ● ● ● ● ● ● ● ● ● ● ● ● ● ● ● ● ● ● ● ● ● ● ● ● ● ● ● ● ● ● ● ● ● ● ● ● ● ● ● ● ● ● ● ● ● ●
 ● ● ● ● ● ● ● ● ● ● ● ● ● ● ● ● ● ● ● ● ● ● ● ● ● ● ● ● ● ● ● ● ● ● ● ● ● ● ● ● ● ● ● ● ● ● ● ● ● ● ● ● ●
● ● ● ● ● ● ● ● ● ● ● ● ● ● ● ● ● ● ● ● ● ● ● ● ● ● ● ● ● ● ● ● ● ● ● ● ● ● ● ● ● ● ● ● ● ● ● ● ● ● ● ● ● ●
 ● ● ● ● ● ● ● ● ● ● ● ● ● ● ● ● ● ● ● ● ● ● ● ● ● ● ● ● ● ● ● ● ● ● ● ● ● ● ● ● ● ● ● ● ● ● ● ● ● ● ● ● ●
● ● ● ● ● ● ● ● ● ● ● ● ● ● ● ● ● ● ● ● ● ● ● ● ● ● ● ● ● ● ● ● ● ● ● ● ● ● ● ● ● ● ● ● ● ● ● ● ● ● ● ● ● ●
 ● ● ● ● ● ● ● ● ● ● ● ● ● ● ● ● ● ● ● ● ● ● ● ● ● ● ● ● ● ● ● ● ● ● ● ● ● ● ● ● ● ● ● ● ● ● ● ● ● ● ● ● ●
● ● ● ● ● ● ● ● ● ● ● ● ● ● ● ● ● ● ● ● ● ● ● ● ● ● ● ● ● ● ● ● ● ● ● ● ● ● ● ● ● ● ● ● ● ● ● ● ● ● ● ● ● ●
 ● ● ● ● ● ● ● ● ● ● ● ● ● ● ● ● ● ● ● ● ● ● ● ● ● ● ● ● ● ● ● ● ● ● ● ● ● ● ● ● ● ● ● ● ● ● ● ● ● ● ● ● ●
● ● ● ● ● ● ● ● ● ● ● ● ● ● ● ● ● ● ● ● ● ● ● ● ● ● ● ● ● ● ● ● ● ● ● ● ● ● ● ● ● ● ● ● ● ● ● ● ● ● ● ● ● ●
 ● ● ● ● ● ● ● ● ● ● ● ● ● ● ● ● ● ● ● ● ● ● ● ● ● ● ● ● ● ● ● ● ● ● ● ● ● ● ● ● ● ● ● ● ● ● ● ● ● ● ● ● ●
● ● ● ● ● ● ● ● ● ● ● ● ● ● ● ● ● ● ● ● ● ● ● ● ● ● ● ● ● ● ● ● ● ● ● ● ● ● ● ● ● ● ● ● ● ● ● ● ● ● ● ● ● ●
 ● ● ● ● ● ● ● ● ● ● ● ● ● ● ● ● ● ● ● ● ● ● ● ● ● ● ● ● ● ● ● ● ● ● ● ● ● ● ● ● ● ● ● ● ● ● ● ● ● ● ● ● ●
● ● ● ● ● ● ● ● ● ● ● ● ● ● ● ● ● ● ● ● ● ● ● ● ● ● ● ● ● ● ● ● ● ● ● ● ● ● ● ● ● ● ● ● ● ● ● ● ● ● ● ● ● ●
 ● ● ● ● ● ● ● ● ● ● ● ● ● ● ● ● ● ● ● ● ● ● ● ● ● ● ● ● ● ● ● ● ● ● ● ● ● ● ● ● ● ● ● ● ● ● ● ● ● ● ● ● ●
● ● ● ● ● ● ● ● ● ● ● ● ● ● ● ● ● ● ● ● ● ● ● ● ● ● ● ● ● ● ● ● ● ● ● ● ● ● ● ● ● ● ● ● ● ● ● ● ● ● ● ● ● ●
 ● ● ● ● ● ● ● ● ● ● ● ● ● ● ● ● ● ● ● ● ● ● ● ● ● ● ● ● ● ● ● ● ● ● ● ● ● ● ● ● ● ● ● ● ● ● ● ● ● ● ● ● ●
● ● ● ● ● ● ● ● ● ● ● ● ● ● ● ● ● ● ● ● ● ● ● ● ● ● ● ● ● ● ● ● ● ● ● ● ● ● ● ● ● ● ● ● ● ● ● ● ● ● ● ● ● ●
 ● ● ● ● ● ● ● ● ● ● ● ● ● ● ● ● ● ● ● ● ● ● ● ● ● ● ● ● ● ● ● ● ● ● ● ● ● ● ● ● ● ● ● ● ● ● ● ● ● ● ● ● ●
● ● ● ● ● ● ● ● ● ● ● ● ● ● ● ● ● ● ● ● ● ● ● ● ● ● ● ● ● ● ● ● ● ● ● ● ● ● ● ● ● ● ● ● ● ● ● ● ● ● ● ● ● ●
 ● ● ● ● ● ● ● ● ● ● ● ● ● ● ● ● ● ● ● ● ● ● ● ● ● ● ● ● ● ● ● ● ● ● ● ● ● ● ● ● ● ● ● ● ● ● ● ● ● ● ● ● ●
● ● ● ● ● ● ● ● ● ● ● ● ● ● ● ● ● ● ● ● ● ● ● ● ● ● ● ● ● ● ● ● ● ● ● ● ● ● ● ● ● ● ● ● ● ● ● ● ● ● ● ● ● ●
 ● ● ● ● ● ● ● ● ● ● ● ● ● ● ● ● ● ● ● ● ● ● ● ● ● ● ● ● ● ● ● ● ● ● ● ● ● ● ● ● ● ● ● ● ● ● ● ● ● ● ● ● ●
● ● ● ● ● ● ● ● ● ● ● ● ● ● ● ● ● ● ● ● ● ● ● ● ● ● ● ● ● ● ● ● ● ● ● ● ● ● ● ● ● ● ● ● ● ● ● ● ● ● ● ● ● ●
 ● ● ● ● ● ● ● ● ● ● ● ● ● ● ● ● ● ● ● ● ● ● ● ● ● ● ● ● ● ● ● ● ● ● ● ● ● ● ● ● ● ● ● ● ● ● ● ● ● ● ● ● ●
● ● ● ● ● ● ● ● ● ● ● ● ● ● ● ● ● ● ● ● ● ● ● ● ● ● ● ● ● ● ● ● ● ● ● ● ● ● ● ● ● ● ● ● ● ● ● ● ● ● ● ● ● ●
 ● ● ● ● ● ● ● ● ● ● ● ● ● ● ● ● ● ● ● ● ● ● ● ● ● ● ● ● ● ● ● ● ● ● ● ● ● ● ● ● ● ● ● ● ● ● ● ● ● ● ● ● ●
● ● ● ● ● ● ● ● ● ● ● ● ● ● ● ● ● ● ● ● ● ● ● ● ● ● ● ● ● ● ● ● ● ● ● ● ● ● ● ● ● ● ● ● ● ● ● ● ● ● ● ● ● ●
 ● ● ● ● ● ● ● ● ● ● ● ● ● ● ● ● ● ● ● ● ● ● ● ● ● ● ● ● ● ● ● ● ● ● ● ● ● ● ● ● ● ● ● ● ● ● ● ● ● ● ● ● ●
● ● ● ● ● ● ● ● ● ● ● ● ● ● ● ● ● ● ● ● ● ● ● ● ● ● ● ● ● ● ● ● ● ● ● ● ● ● ● ● ● ● ● ● ● ● ● ● ● ● ● ● ● ●
 ● ● ● ● ● ● ● ● ● ● ● ● ● ● ● ● ● ● ● ● ● ● ● ● ● ● ● ● ● ● ● ● ● ● ● ● ● ● ● ● ● ● ● ● ● ● ● ● ● ● ● ● ●
● ● ● ● ● ● ● ● ● ● ● ● ● ● ● ● ● ● ● ● ● ● ● ● ● ● ● ● ● ● ● ● ● ● ● ● ● ● ● ● ● ● ● ● ● ● ● ● ● ● ● ● ● ●
 ● ● ● ● ● ● ● ● ● ● ● ● ● ● ● ● ● ● ● ● ● ● ● ● ● ● ● ● ● ● ● ● ● ● ● ● ● ● ● ● ● ● ● ● ● ● ● ● ● ● ● ● ●
● ● ● ● ● ● ● ● ● ● ● ● ● ● ● ● ● ● ● ● ● ● ● ● ● ● ● ● ● ● ● ● ● ● ● ● ● ● ● ● ● ● ● ● ● ● ● ● ● ● ● ● ● ●
 ● ● ● ● ● ● ● ● ● ● ● ● ● ● ● ● ● ● ● ● ● ● ● ● ● ● ● ● ● ● ● ● ● ● ● ● ● ● ● ● ● ● ● ● ● ● ● ● ● ● ● ● ●
● ● ● ● ● ● ● ● ● ● ● ● ● ● ● ● ● ● ● ● ● ● ● ● ● ● ● ● ● ● ● ● ● ● ● ● ● ● ● ● ● ● ● ● ● ● ● ● ● ● ● ● ● ●
 ● ● ● ● ● ● ● ● ● ● ● ● ● ● ● ● ● ● ● ● ● ● ● ● ● ● ● ● ● ● ● ● ● ● ● ● ● ● ● ● ● ● ● ● ● ● ● ● ● ● ● ● ●
● ● ● ● ● ● ● ● ● ● ● ● ● ● ● ● ● ● ● ● ● ● ● ● ● ● ● ● ● ● ● ● ● ● ● ● ● ● ● ● ● ● ● ● ● ● ● ● ● ● ● ● ● ●
 ● ● ● ● ● ● ● ● ● ● ● ● ● ● ● ● ● ● ● ● ● ● ● ● ● ● ● ● ● ● ● ● ● ● ● ● ● ● ● ● ● ● ● ● ● ● ● ● ● ● ● ● ●
● ● ● ● ● ● ● ● ● ● ● ● ● ● ● ● ● ● ● ● ● ● ● ● ● ● ● ● ● ● ● ● ● ● ● ● ● ● ● ● ● ● ● ● ● ● ● ● ● ● ● ● ● ●
 ● ● ● ● ● ● ● ● ● ● ● ● ● ● ● ● ● ● ● ● ● ● ● ● ● ● ● ● ● ● ● ● ● ● ● ● ● ● ● ● ● ● ● ● ● ● ● ● ● ● ● ● ●
● ● ● ● ● ● ● ● ● ● ● ● ● ● ● ● ● ● ● ● ● ● ● ● ● ● ● ● ● ● ● ● ● ● ● ● ● ● ● ● ● ● ● ● ● ● ● ● ● ● ● ● ● ●
 ● ● ● ● ● ● ● ● ● ● ● ● ● ● ● ● ● ● ● ● ● ● ● ● ● ● ● ● ● ● ● ● ● ● ● ● ● ● ● ● ● ● ● ● ● ● ● ● ● ● ● ● ●
