# Supplementary material for: The loss and recovery of vertebrate vision examined in microplates
Source: PLoS One. 2017 Aug 17;12(8):e0183414. doi: 10.1371/journal.pone.0183414 (PMC5560659; doi:10.1371/journal.pone.0183414)
Supplement: S5 File — (PPTX) [file pone.0183414.s006.pptx]

## Slide 1
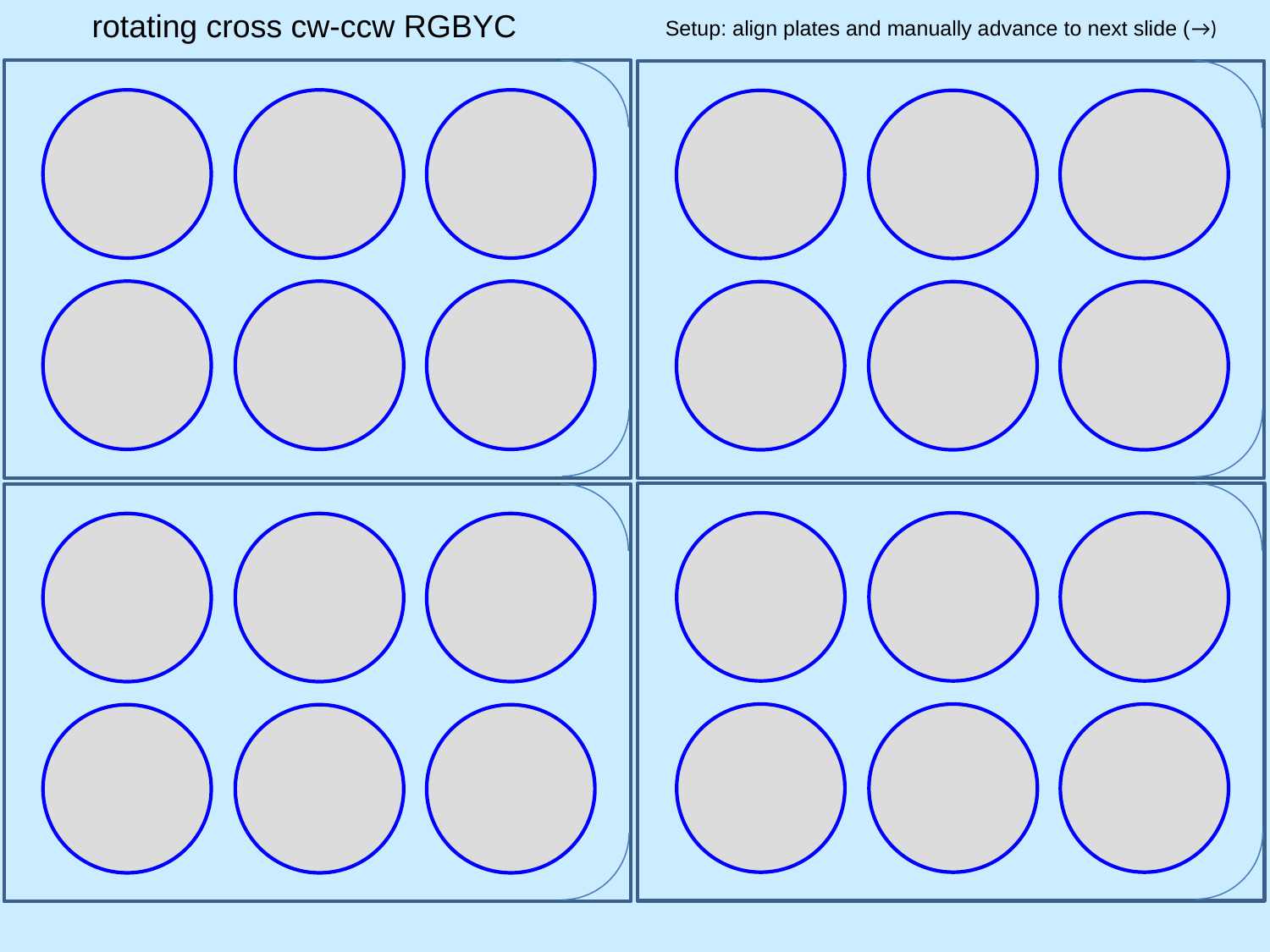

rotating cross cw-ccw RGBYC
Setup: align plates and manually advance to next slide (→)

## Slide 2
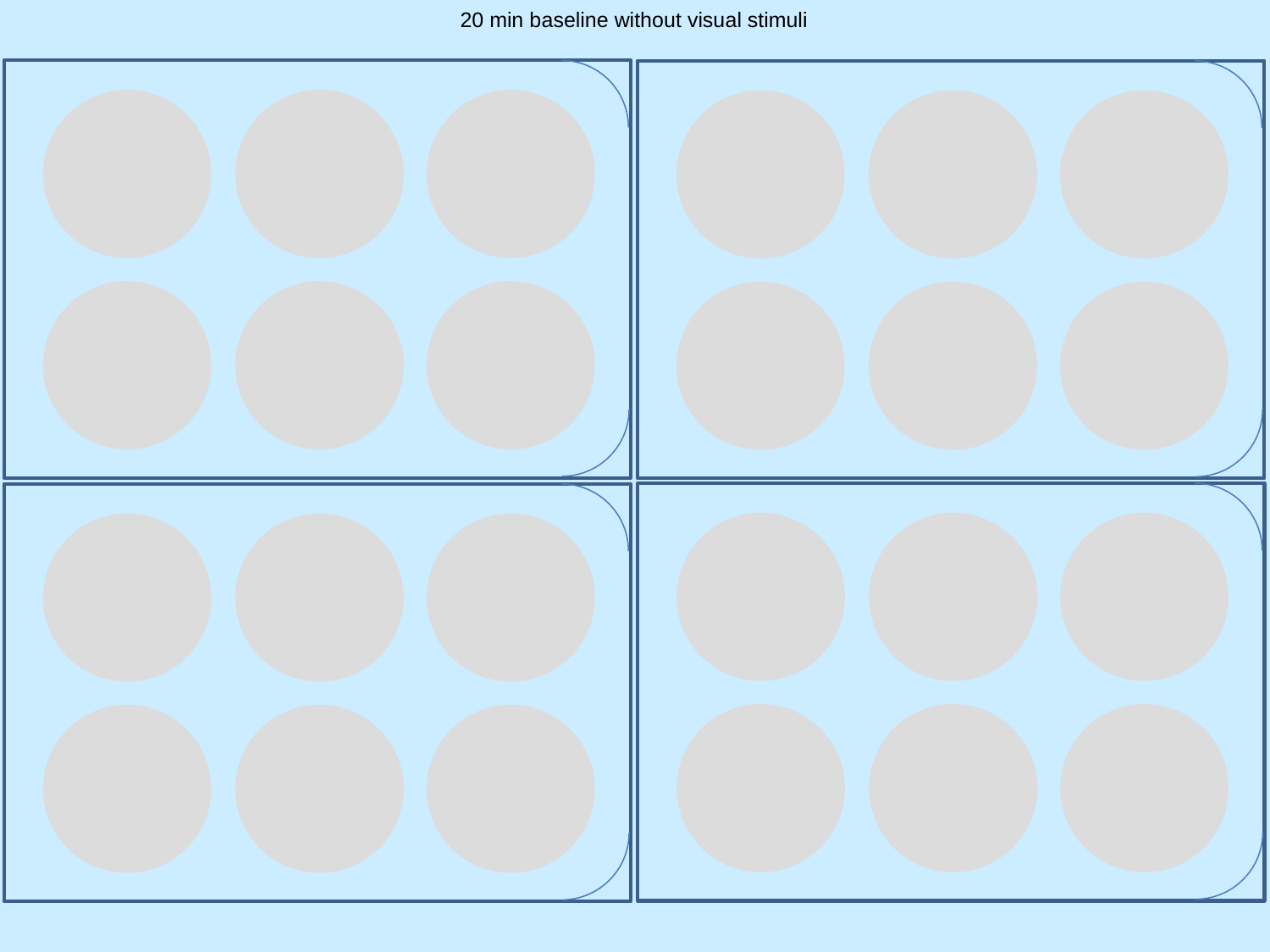

20 min baseline without visual stimuli

## Slide 3
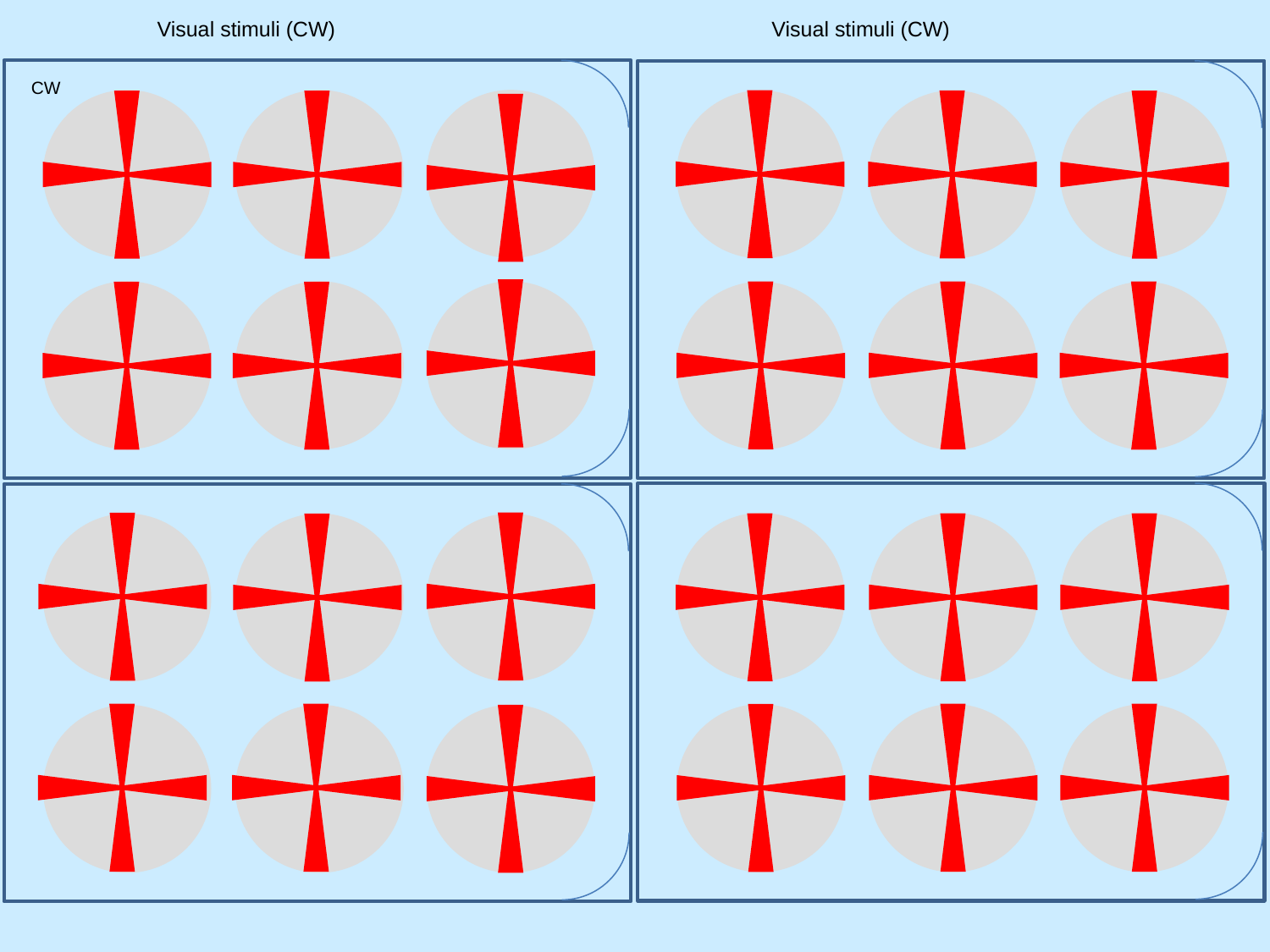

Visual stimuli (CW)
Visual stimuli (CW)
CW

## Slide 4
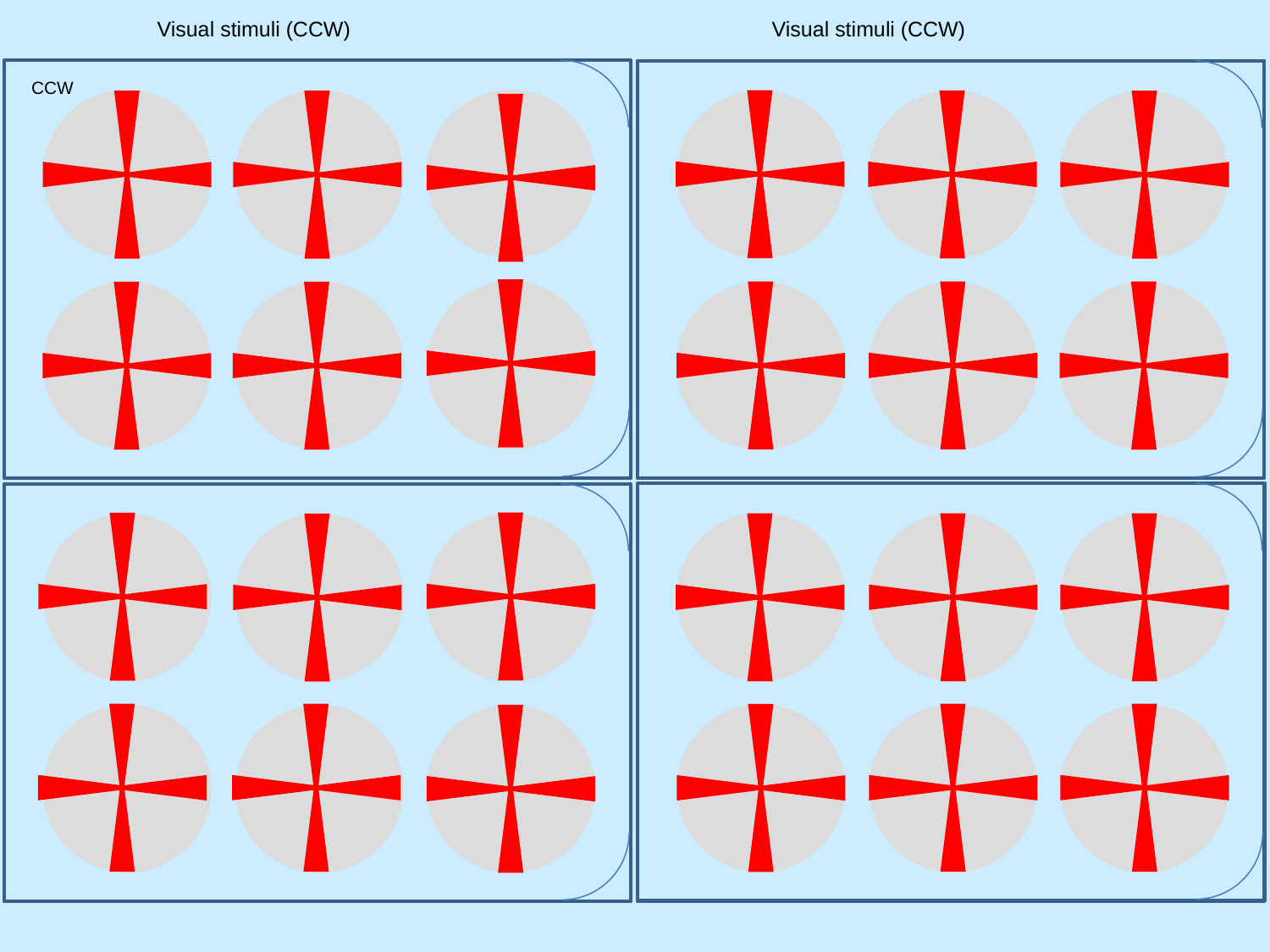

Visual stimuli (CCW)
Visual stimuli (CCW)
CCW

## Slide 5
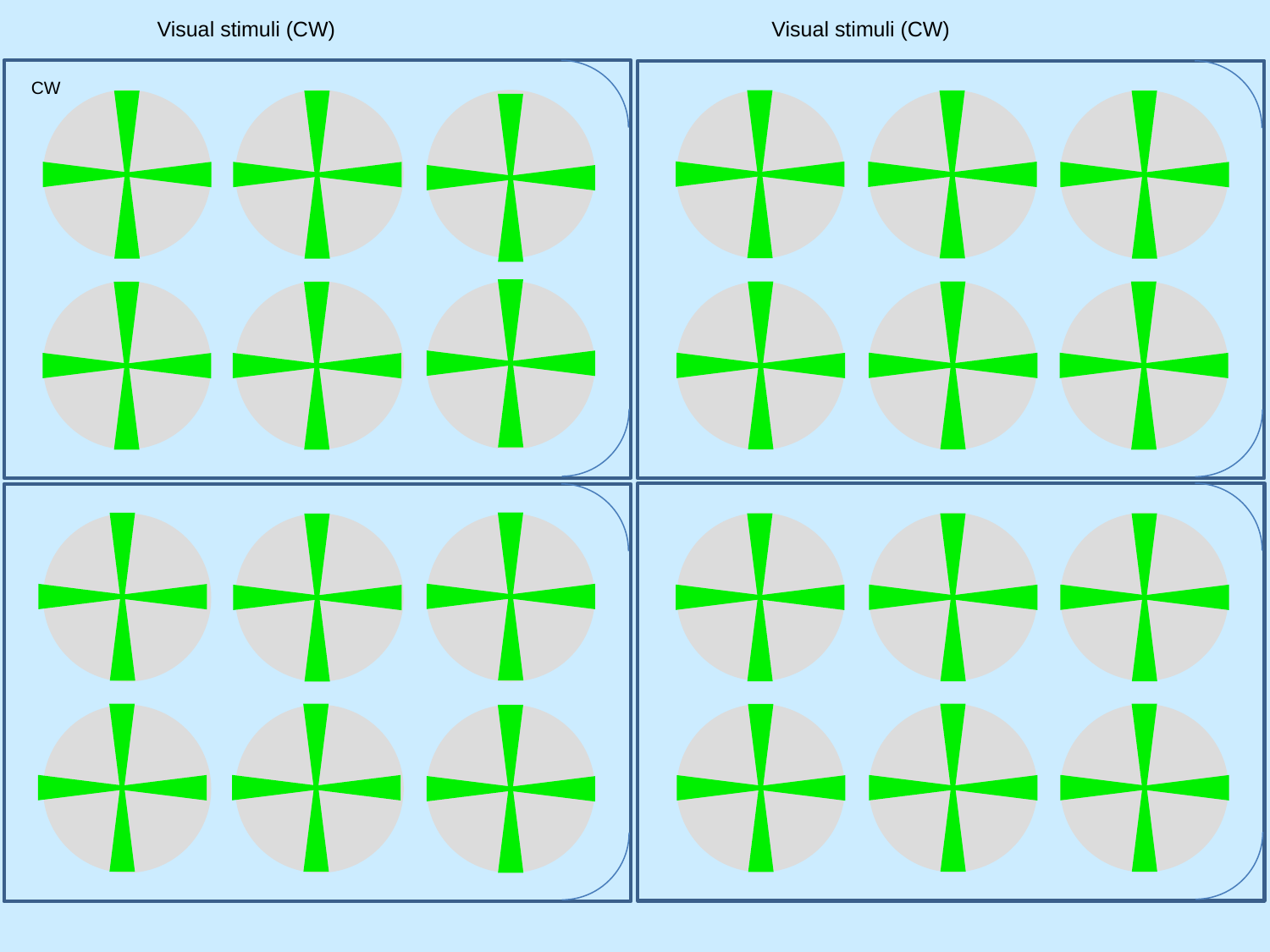

Visual stimuli (CW)
Visual stimuli (CW)
CW

## Slide 6
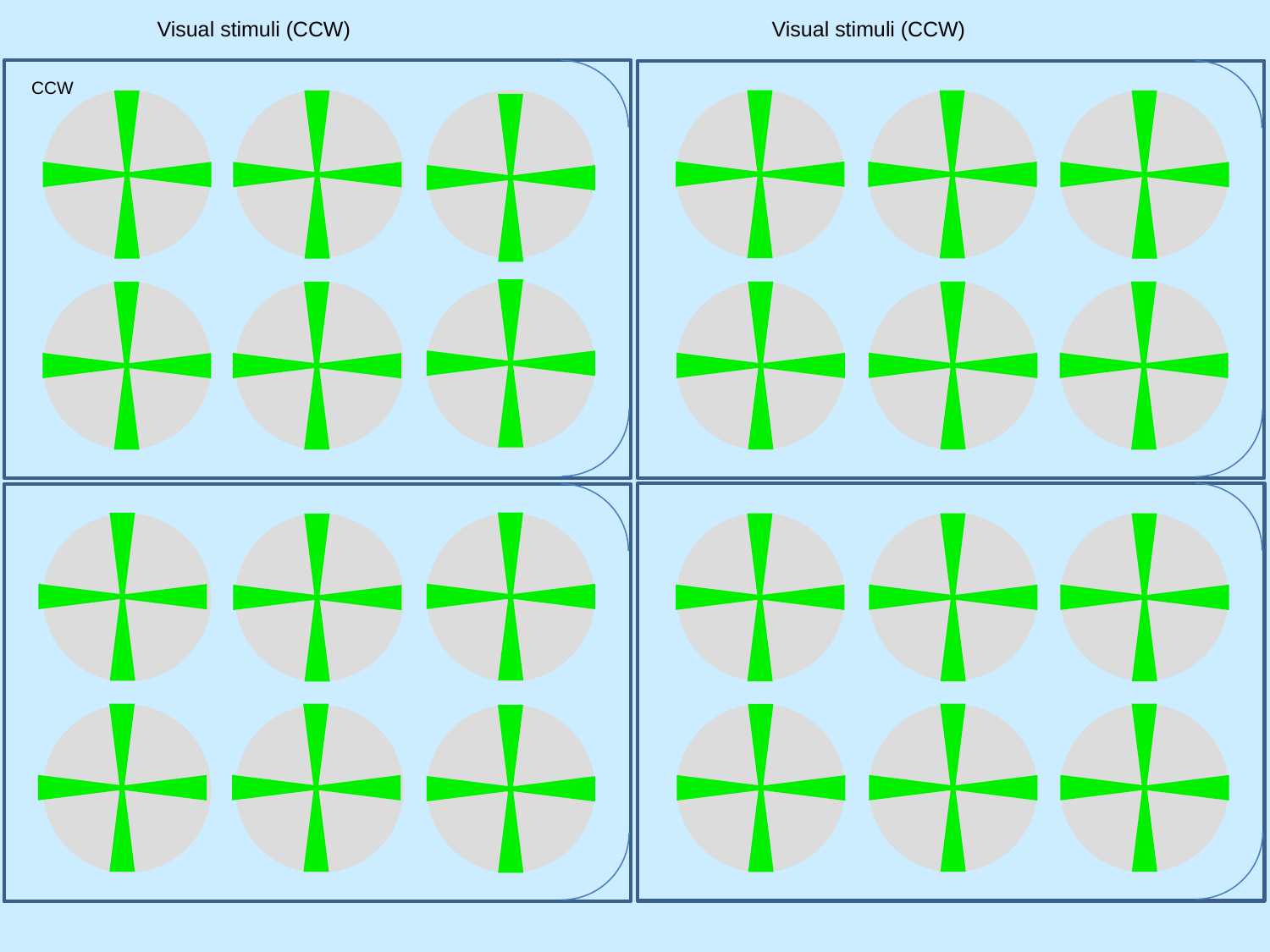

Visual stimuli (CCW)
Visual stimuli (CCW)
CCW

## Slide 7
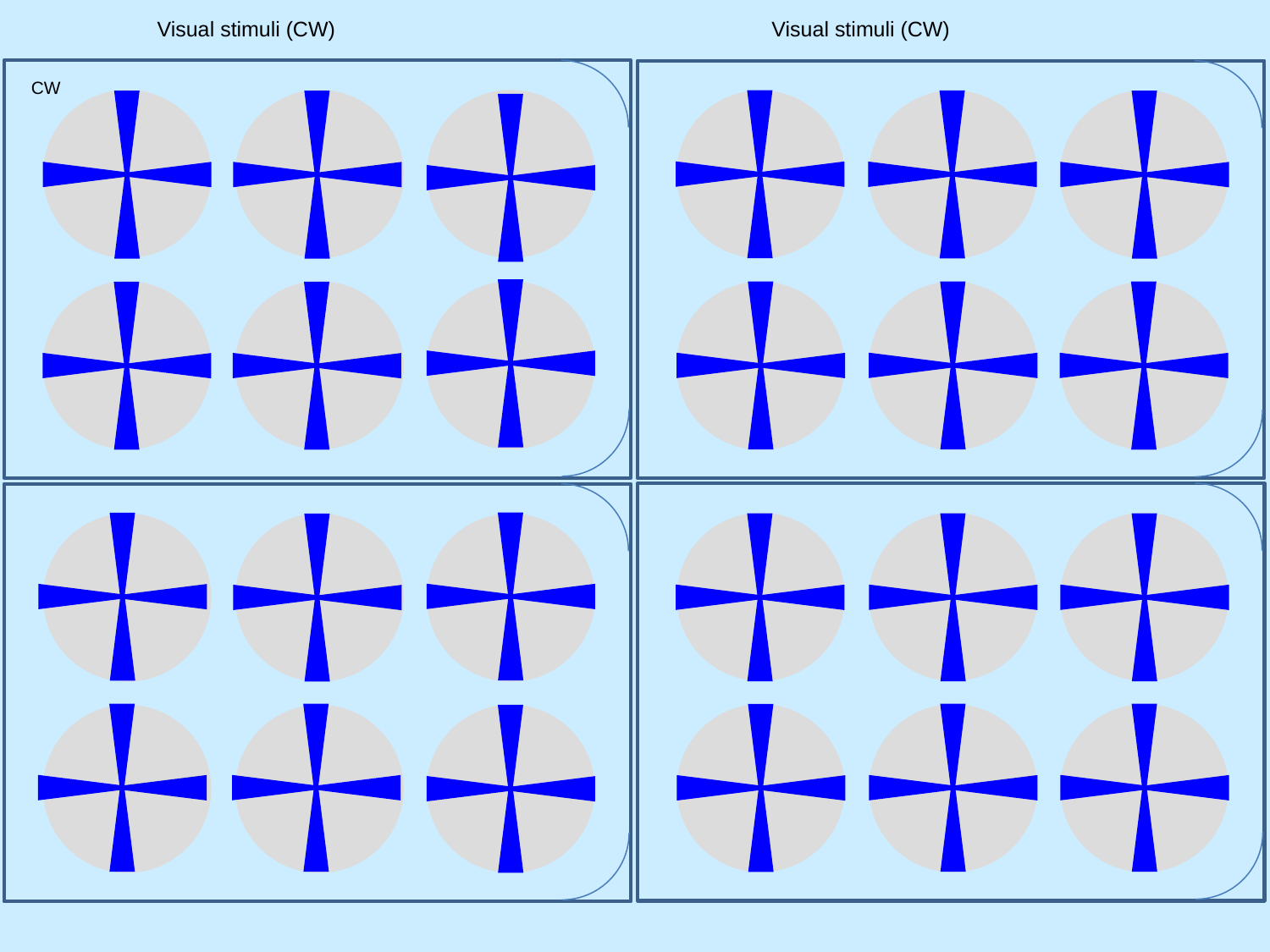

Visual stimuli (CW)
Visual stimuli (CW)
CW

## Slide 8
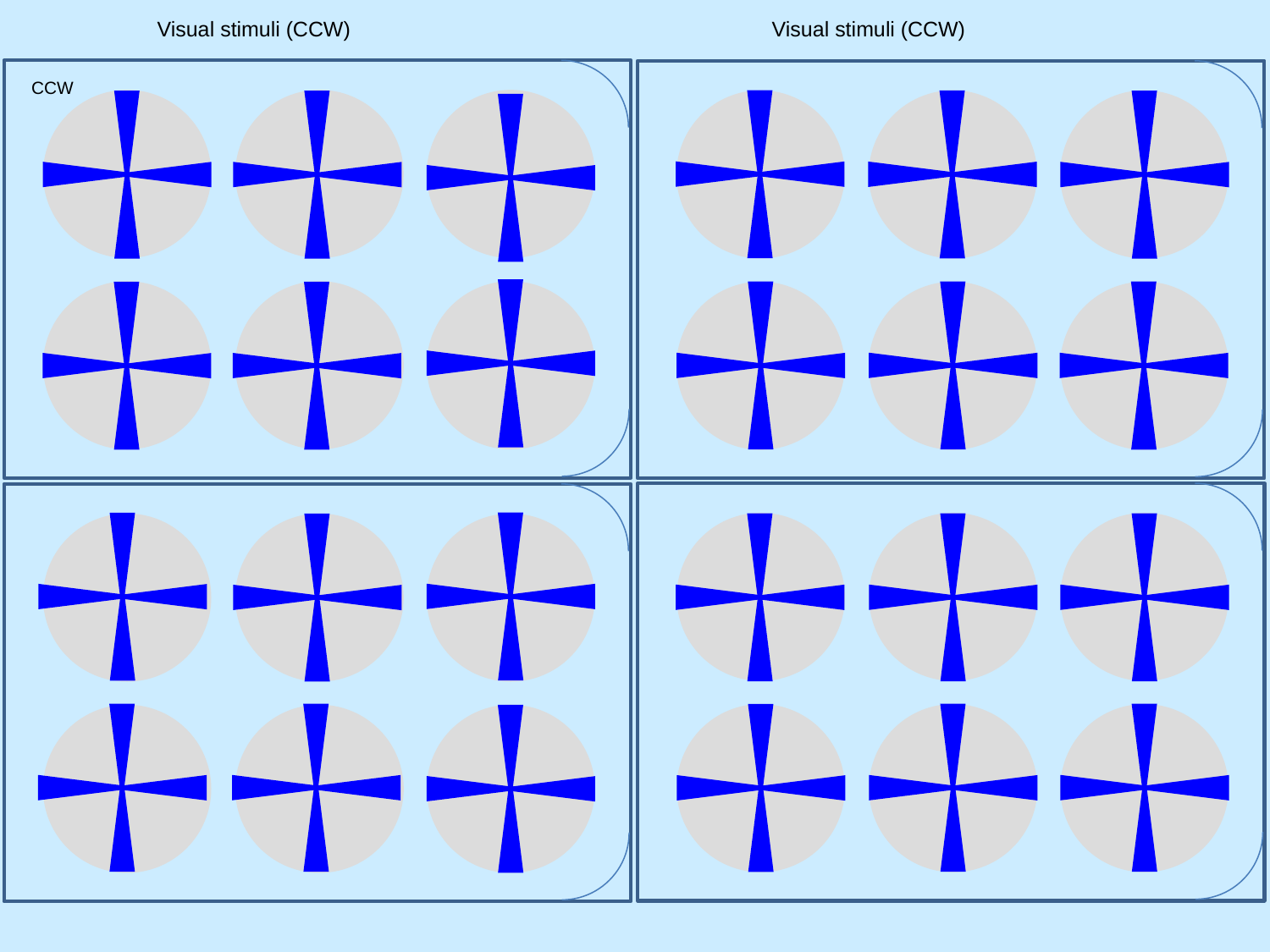

Visual stimuli (CCW)
Visual stimuli (CCW)
CCW

## Slide 9
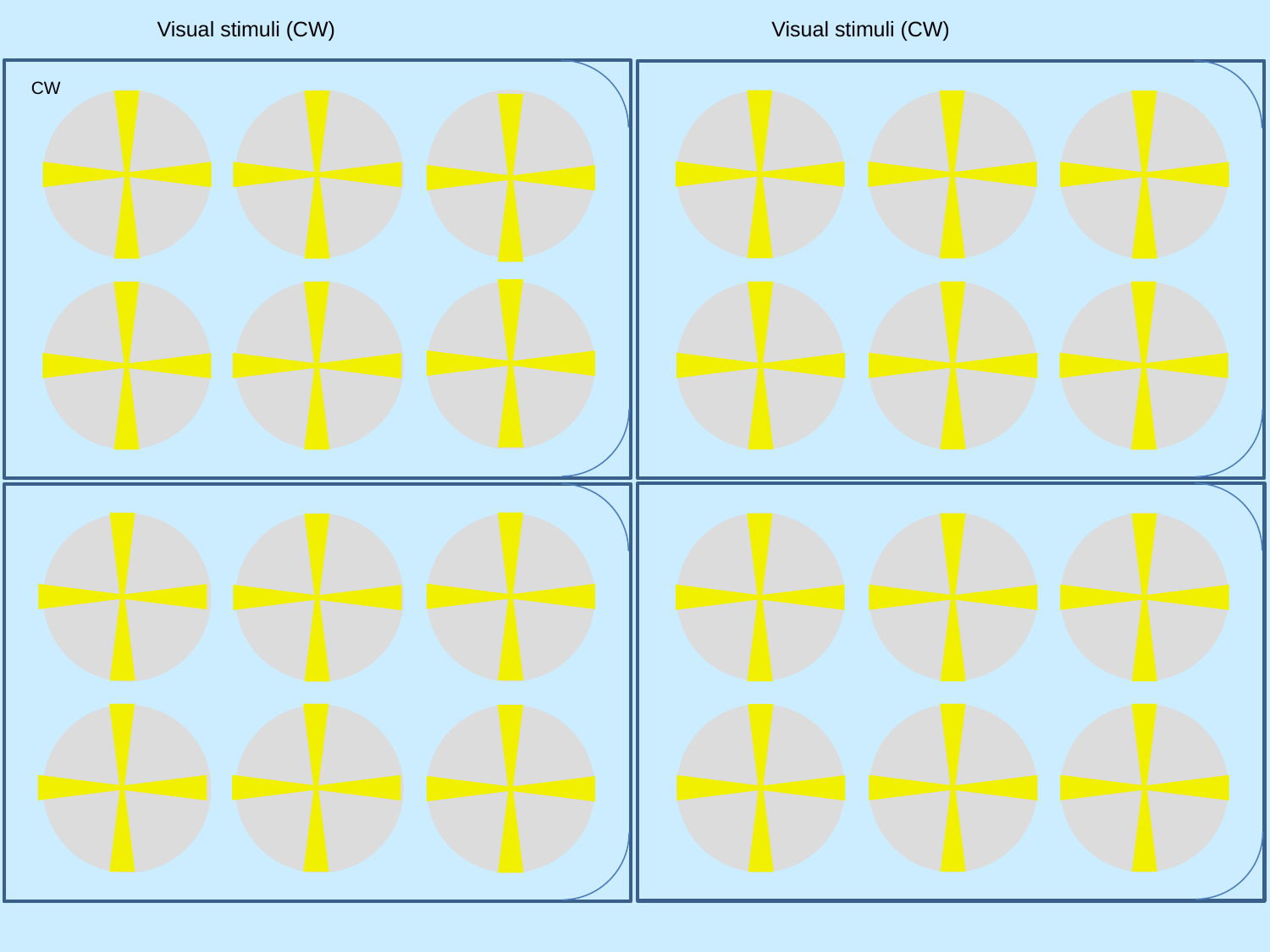

Visual stimuli (CW)
Visual stimuli (CW)
CW

## Slide 10
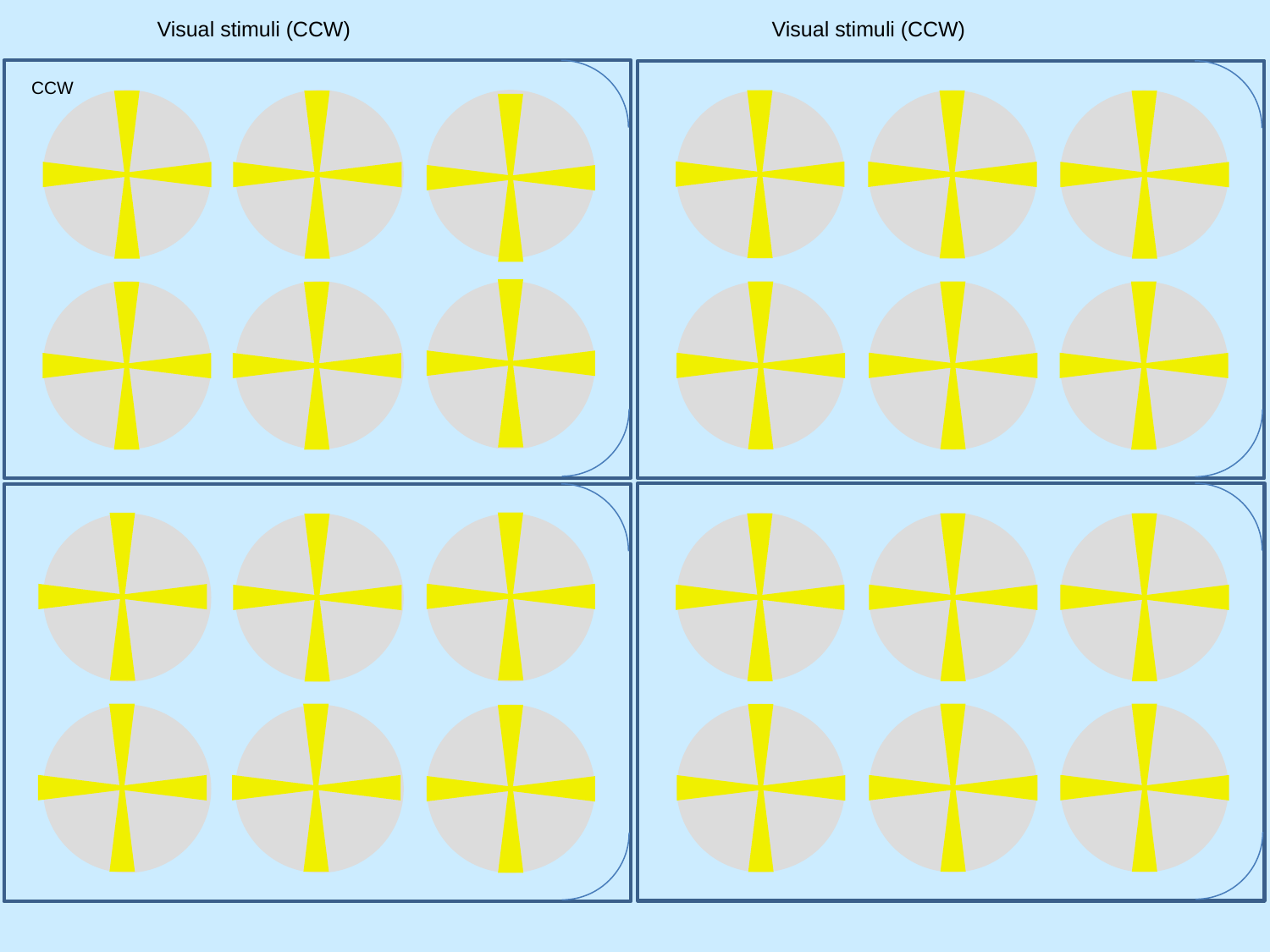

Visual stimuli (CCW)
Visual stimuli (CCW)
CCW

## Slide 11
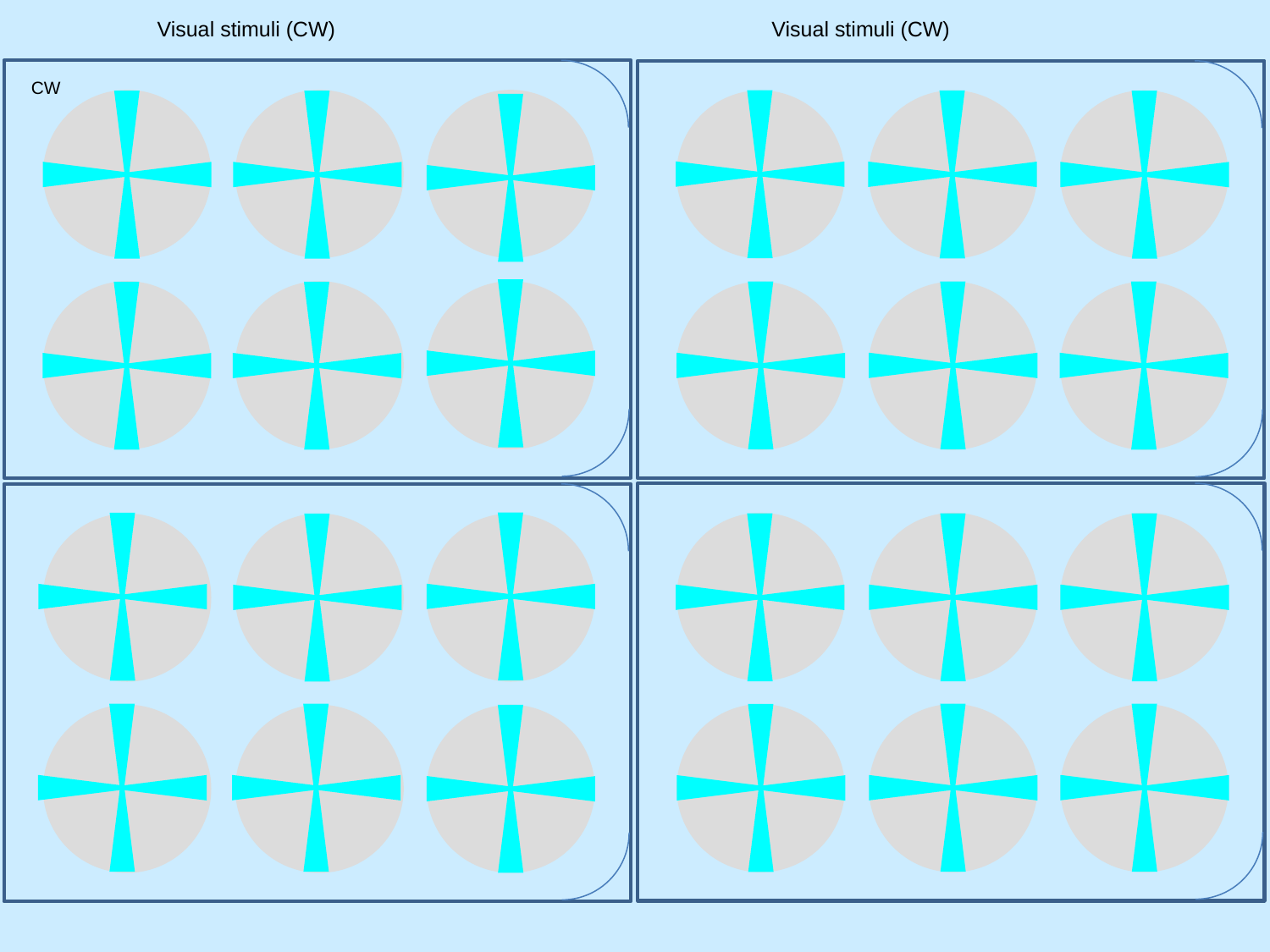

Visual stimuli (CW)
Visual stimuli (CW)
CW

## Slide 12
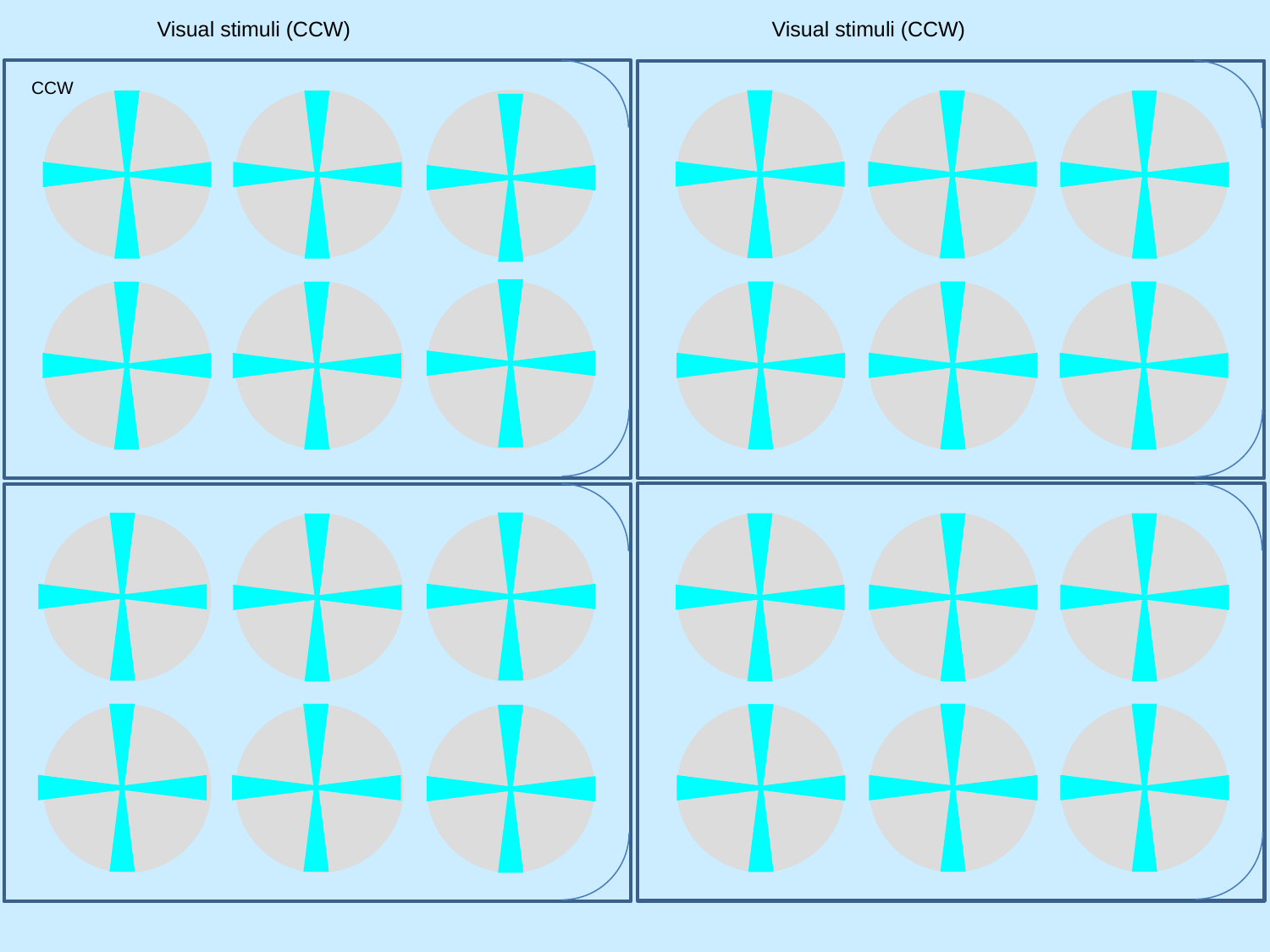

Visual stimuli (CCW)
Visual stimuli (CCW)
CCW
